# Transforming L1000 profiles to RNA-seq-like profiles with deep learning

**DOI:** 10.1186/s12859-022-04895-5

**Published:** 2022-09-13

**Authors:** Minji Jeon, Zhuorui Xie, John E. Evangelista, Megan L. Wojciechowicz, Daniel J. B. Clarke, Avi Ma’ayan

**Affiliations:** 1 Department of Pharmacological Sciences, Mount Sinai Center for Bioinformatics, Icahn School of Medicine at Mount Sinai, One Gustave L. Levy Place, Box 1603, New York, NY 10029 USA; 2 Department of Medicine, Korea University College of Medicine, Seoul, Republic of Korea

**Keywords:** L1000, RNA-seq, Gene expression translation, Generative adversarial networks

## Abstract

The L1000 technology, a cost-effective high-throughput transcriptomics technology, has been applied to profile a collection of human cell lines for their gene expression response to > 30,000 chemical and genetic perturbations. In total, there are currently over 3 million available L1000 profiles. Such a dataset is invaluable for the discovery of drug and target candidates and for inferring mechanisms of action for small molecules. The L1000 assay only measures the mRNA expression of 978 landmark genes while 11,350 additional genes are computationally reliably inferred. The lack of full genome coverage limits knowledge discovery for half of the human protein coding genes, and the potential for integration with other transcriptomics profiling data. Here we present a Deep Learning two-step model that transforms L1000 profiles to RNA-seq-like profiles. The input to the model are the measured 978 landmark genes while the output is a vector of 23,614 RNA-seq-like gene expression profiles. The model first transforms the landmark genes into RNA-seq-like 978 gene profiles using a modified CycleGAN model applied to unpaired data. The transformed 978 RNA-seq-like landmark genes are then extrapolated into the full genome space with a fully connected neural network model. The two-step model achieves 0.914 Pearson’s correlation coefficients and 1.167 root mean square errors when tested on a published paired L1000/RNA-seq dataset produced by the LINCS and GTEx programs. The processed RNA-seq-like profiles are made available for download, signature search, and gene centric reverse search with unique case studies.

**Supplementary Information:**

The online version contains supplementary material available at 10.1186/s12859-022-04895-5.

## Background

Transcriptomics profiling is currently the most comprehensive and accurate method to profile the molecular state of cells at the genome-wide scale. The consistent drop in cost and improvements in quality make genome-wide transcriptomics a central method in biomedical and biological research. Typically, transcriptomics profiling is applied to produce gene expression signatures by comparing a control condition to a perturbed condition. The genes that are differentially expressed as the consequence of the perturbation can give clues about the underlying molecular mechanisms at play. The Library of Integrated Network-Based Cellular Signatures (LINCS) NIH Common Fund program was established to accelerate the discovery of small molecule therapeutics by profiling human cell lines with omics technologies before and after these cells are perturbed [1]. The L1000 assay is a low-cost transcriptomics technology that was applied in high throughput to generate millions of gene expression signatures for LINCS [2]. Using the L1000 assay, the LINCS Transcriptomics Data and Signature Generation Center (DSGC) at the Broad Institute produced over 3 million expression profiles measuring gene expression of human cell lines treated with over 30,000 chemical and genetic perturbations. This dataset, which is made publicly available at the clue.io platform, has the potential to facilitate rapid discovery of drug candidates, and significantly accelerate our understanding of the molecular mechanisms induced by small molecules and genetic perturbations. However, although the L1000 assay produced high-quality data, it only measured the mRNA expression of 978 landmark genes. These landmark genes were pre-selected based on their orthogonality with the rest of the transcriptome to maximize the ability to infer the expression of the rest of the genes computationally. Currently, an additional set of 11,350 genes are computationally reliably inferred. These expression profiles are provided to researchers for download and analysis. However, this leaves about half of the protein coding mRNAs expression levels missing. This limits our ability to properly identify differentially expressed pathways, integrate the L1000 data with other transcriptomics data, and study the expression and activity of many unmeasured and un-inferred genes.

To mitigate the deficiency with the currently available L1000 profiles, a computational model that translates data from one format to another might be of utility. In recent years, significant progress was made in computer-aided language and image translation with Deep Learning. For example, unpaired image-to-image translation with CycleGAN [3] was used to learn a mapping between one image domain to another using an unsupervised approach. CycleGAN employs a generative adversarial network (GAN) architecture [4] composed of two generators and two discriminators. The generators take an image from one domain and convert it to make it look like it came from the other domain. The discriminators attempt to predict whether a given image is real or generated. The discriminators and generators work against each other until the discriminators can no longer distinguish the difference between real and generated images. CycleGAN introduces the concept of cycle-consistency. An image output by the first generator is used as the input to the second generator where the output of the second generator should match the original image.

Several studies employed GANs and feed-forward neural networks to transform gene expression profiles for various tasks including for the analysis of L1000 data. For example, GGAN [5] is a conditional generative adversarial network model with one generator and one discriminator that takes gene expression of L1000 landmark genes as input and predicts gene expression for 9520 unmeasured genes. The generator takes as input gene expression profiles of landmark genes and generates the expression of the 9520 genes. The discriminator predicts whether the gene expression profiles of the remaining genes are real or generated. D-GEX [6] is a multi-task multi-layer feedforward neural network that also takes as input the landmark L1000 gene expression profiles and predicts the expression of 11,350 genes. GGAN and D-GEX improve the original inference algorithm developed by the Connectivity Map (CMAP) team at the Broad Institute which uses linear regression, potentially missing non-linear relationships known to exist within gene expression data. In addition, Ghahramani et al. used GANs to reduce the dimensionality of single cell RNA-seq profiles and to predict perturbations [7]. Lee and Ahn used the CycleGAN architecture to transform gene expression patterns from tumors into their corresponding normal-tissue profiles [8], while several other groups developed other applications within this domain. However, these previous methods were applied to generating gene expression profiles for a given input in the same domain, not for converting profiles across experimental platforms. The prior implementations are also applied to a limited set of genes, missing the measurements of many protein-coding and non-coding mRNAs.

**Fig. 1:**
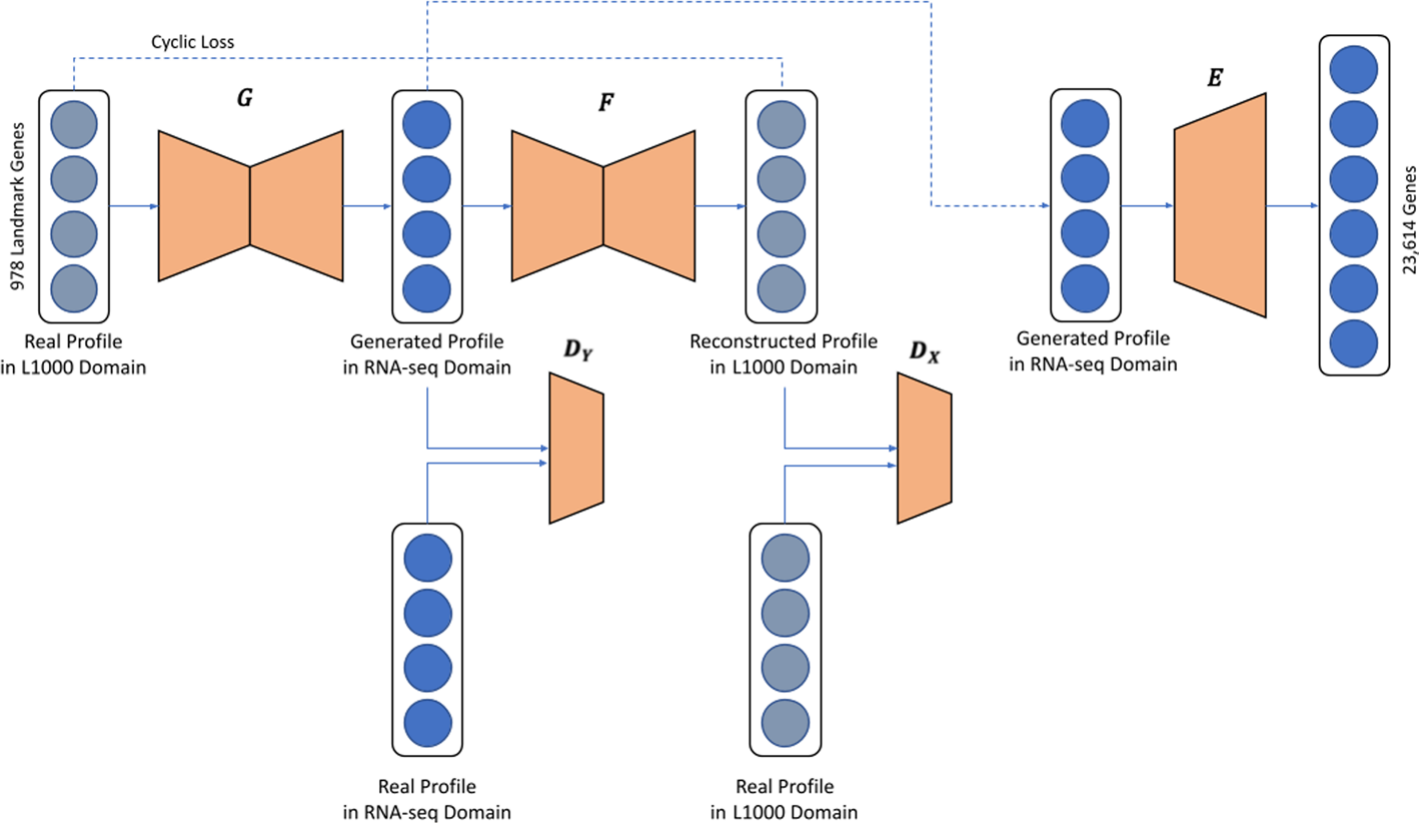
Model architecture**.** *G* and *F* are generators, *D*_*Y*_ and *D*_*X*_ are discriminators, and *E *is a fully connected neural network model for RNA-seq profile extrapolation from landmark genes to the full genome. The pipeline inputs are measured expression of 978 landmark genes from L1000 profiles. The CycleGAN model in the pipeline predicts RNA-seq-like profiles for given L1000 profiles. The extrapolation model inputs are 978-dimensional vectors of predicted RNA-seq-like profiles. The model then predicts 23,614-dimensional vectors of RNA-seq-like profiles

Here we present a two-step Deep Learning model that reliably converts L1000 profiles to RNA-seq-like profiles. The first step of the model takes as input the measured gene expression levels of the 978 landmark genes and converts these vectors into RNA-seq-like 978 gene vectors using a modified CycleGAN model. The second step of the model extrapolates the RNA-seq-like 978 gene vectors into 23,614-dimensional RNA-seq-like whole genome profiles using a fully connected Neural Network (FCNN) model (Fig. 1). To the best of our knowledge, this is the first attempt to transform L1000 profiles into full RNA-seq-like profiles with Deep Learning.

## Results

**Fig. 2:**
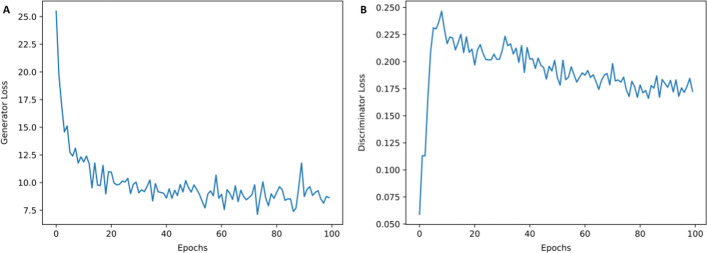
Training progress over epochs. **A** Loss curve during the training of the generator of the cycleGAN. **B** Loss curve during the training of the discriminator of the cycleGAN

The first step of the translator model is to transform L1000 profiles to RNA-seq-like profiles at the landmark gene space using a CycleGAN model. During training, the discriminators and generators compete against each other but over time both discriminator and generator converge (Fig. 2). We see that the loss of the generator improves while the discriminator worsens during the first 10 epochs and then the losses converge after 80 epochs.

### Benchmarking the first step of the model

**Fig. 3:**
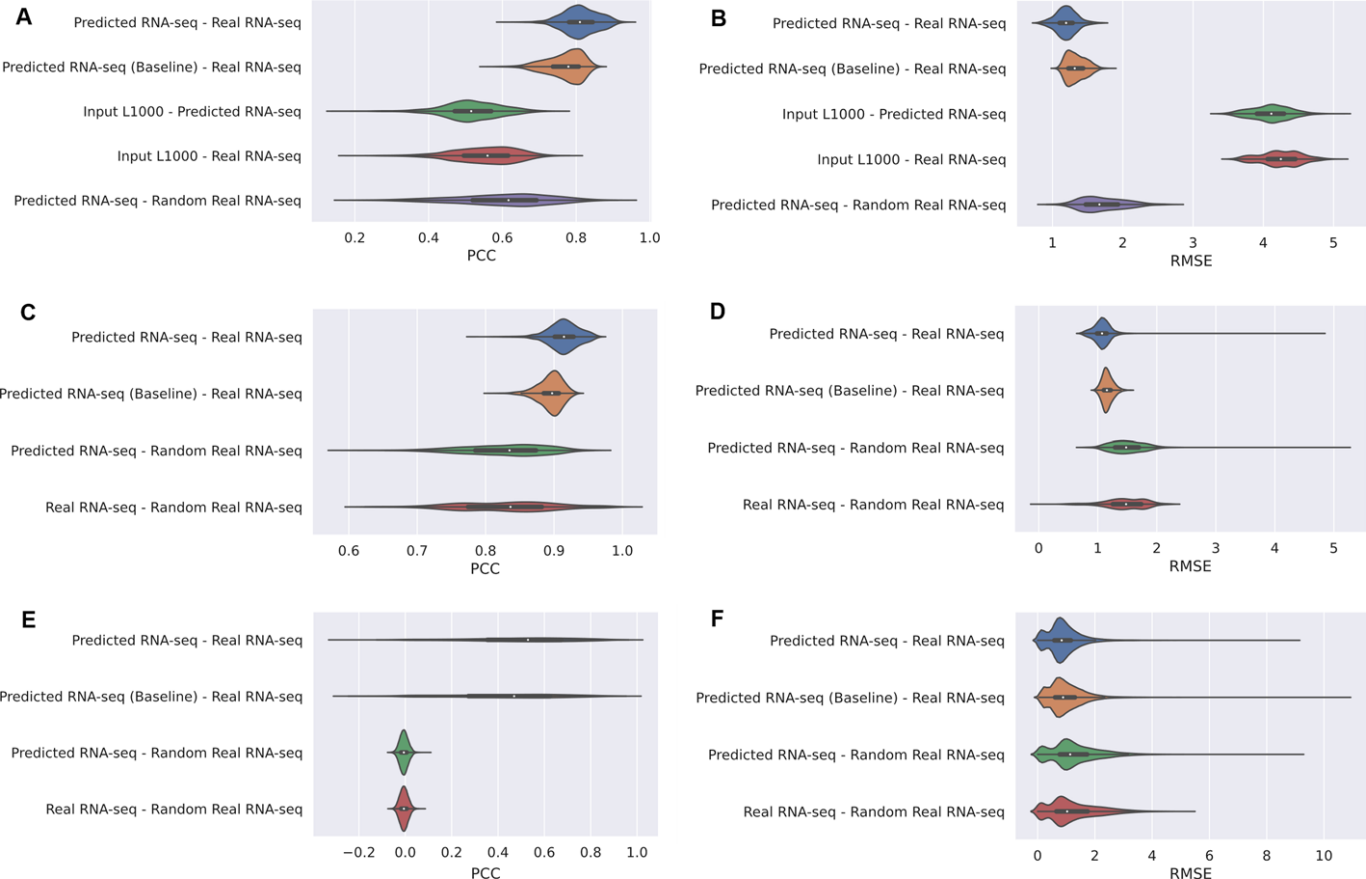
Comparing similarity between predicted and real profiles at the 978-landmark space (**A**, **B**). Violin plots of sample-wise Pearson’s correlation coefficients (PCCs) (**A**) and RSME (**B**) between predicted and real RNA-seq profiles (blue); between predicted by a baseline model and real RNA-seq profiles (orange); input L1000 signatures and predicted RNA-seq-like profiles (green); input L1000 and real RNA-seq profiles (red); and between predicted RNA-seq profiles and randomly paired real RNA-seq profiles (purple). Comparing similarity between predicted and real profiles at the full genome space (**C**, **D**). Violin plots of sample-wise Pearson’s correlation coefficients (**C**) and RSMEs (**D**) between predicted and real RNA-seq profiles (blue), between predicted RNA-seq profiles and randomly paired real RNA-seq profiles (orange), and between real RNA-seq profiles and randomly paired real RNA-seq profiles (green). Comparing similarity between predicted and real profiles at the gene level in the full genome space (**E**, **F**). Violin plots of gene-wise PCCs (**E**) and RSMEs (**F**) between predicted and real RNA-seq profiles (blue), between predicted RNA-seq profiles and randomly paired real RNA-seq profiles (orange), and between real RNA-seq profiles and randomly paired real RNA-seq profiles (green)

The GTEx [9] and LINCS [2] programs collected comprehensive tissue-specific gene expression profiles using paired L1000 and RNA-seq profiles from the same samples (GEO Accession # GSE92743). These 2929 paired samples are used to evaluate the performance of the trained model. The Root Mean Squared Error (RMSE), Pearson’s correlation coefficients (PCCs), and Spearman’s correlation coefficients (SCCs) for each profile are used as the evaluation measures (Fig. 3). To compare the model performance with a baseline, we implemented an algorithm that is based on the ratio between averaged gene expression of L1000 profiles and averaged gene expression of RNA-seq profiles. The ratio is used for scaling the GTEx L1000 profiles, and the scaled profiles are considered as the predicted RNA-seq-like profiles using the conversion baseline model. We measured PCCs between the predicted RNA-seq-like profiles produced by the baseline model and the real RNA-seq profiles, between the input L1000 profiles and the predicted RNA-seq-like profiles with the CycleGAN model, between the input L1000 profiles and the real RNA-seq profiles, and between the predicted RNA-seq-like profiles and randomly paired real RNA-seq profiles. The results from the CycleGAN model achieved an average of 0.812 PCC with a standard deviation of 0.047 when compared with the ground truth. The performance of the baseline model and the other comparisons are AVG = 0.769 (SD = 0.049), AVG = 0.518 (SD = 0.078), AVG = 0.551 (SD = 0.086), and AVG = 0.602 (SD = 0.127), respectively (Fig. 3A). The average RMSE between the CycleGAN RNA-seq-like profiles and the real RNA-seq profiles is 1.200 (0.150), while the average RMSE of the baseline model and other comparisons are AVG = 1.336 (SD = 0.138), AVG = 4.102 (SD = 0.276), AVG = 4.243 (SD = 0.288), and AVG = 1.713 (SD = 0.312), respectively (Fig. 3B). The results measured by SCCs are similar to those computed with PCCs (Additional file 1: Fig. S1). Overall, these results suggest that the modified CycleGAN model reliably translates L1000 profiles to RNA-seq-like profiles at the landmark gene space.

### Benchmarking the second step of the model

The second step of the model takes the predicted RNA-seq-like profiles at the landmark gene space from the first step as input, and then uses these profiles to predict RNA-seq-like profiles at the genome-wide scale. PCCs were calculated for each paired predicted and real full profiles and the average of the coefficients of the 2929 paired GTEx-LINCS samples is computed. To compare the performance with a baseline, we implemented a linear regression model trained on converting RNA-seq gene expression profiles in the landmark gene space into RNA-seq-like gene expression profiles in the full genome space. To use the trained linear regression model as a baseline model, the linear regression model takes predicted GTEx profiles by the conversion method from Step 1 and returns predicted RNA-seq-like profiles at the full genome space. We also measured PCCs between the predicted full-genome RNA-seq-like profiles produced by the baseline model, between predicted full genome-wide RNA-seq-like profiles and randomly paired real full genome RNA-seq profiles. As a result, the final two-step model achieved on average a PCC of 0.914 with a standard deviation of 0.023 (Fig. 3C). The baseline model linear regression model achieves a PCC of 0.895 with a standard deviation of 0.018. The performance of the random baseline was 0.827 with a standard deviation of 0.059. The reason for the relatively high performance of the baseline models is because there are similar patterns between genes in RNA-seq profiles regardless of conditions such as perturbations and tissue types. The average RMSE between the predicted and real profiles is 1.167 with SD of 0.496; and the RMSE between predicted and random samples is 2.320 (SD = 0.933) (Fig. 3D). The results measured by SCC are similar (Additional file 1: Fig. S2). The performance of the model can be computed in two ways, sample-wise and gene-wise. Hence, instead of correlations and distances between samples, we can compute the correlations and distances across samples for each gene. The average PCC of the model across all samples for each gene is AVG = 0.502 (SD = 0.255) and the PCC for the baseline is AVG = 0.442 (SD = 0.236), the PCC for the random baseline is 0.0 (SD = 0.018) (Fig. 3E). The average RMSE across all samples for each gene is 0.910 (SD = 0.582) compared to a baseline of 0.998 (SD = 0.609) (Fig. 3F). The results measured by SCCs are similar (Additional file 1: Fig. S3). Overall, the model achieves lower RMSE for 95.55% of the genes and higher PCC for 98.30% of the genes. These results suggest that the second step of the model reliably predicts full genome-wide RNA-seq-like profiles given L1000 landmark profiles. Supplementary figures of gene-wise PCC, RMSE, and SCC using the landmark gene space, the sample-wise and gene-wise PCC, RMSE and SCC using only newly inferred genes are provided (Additional file 1: Figs. S4–S12).

### Visualization of signatures in 2D space

**Fig. 4:**
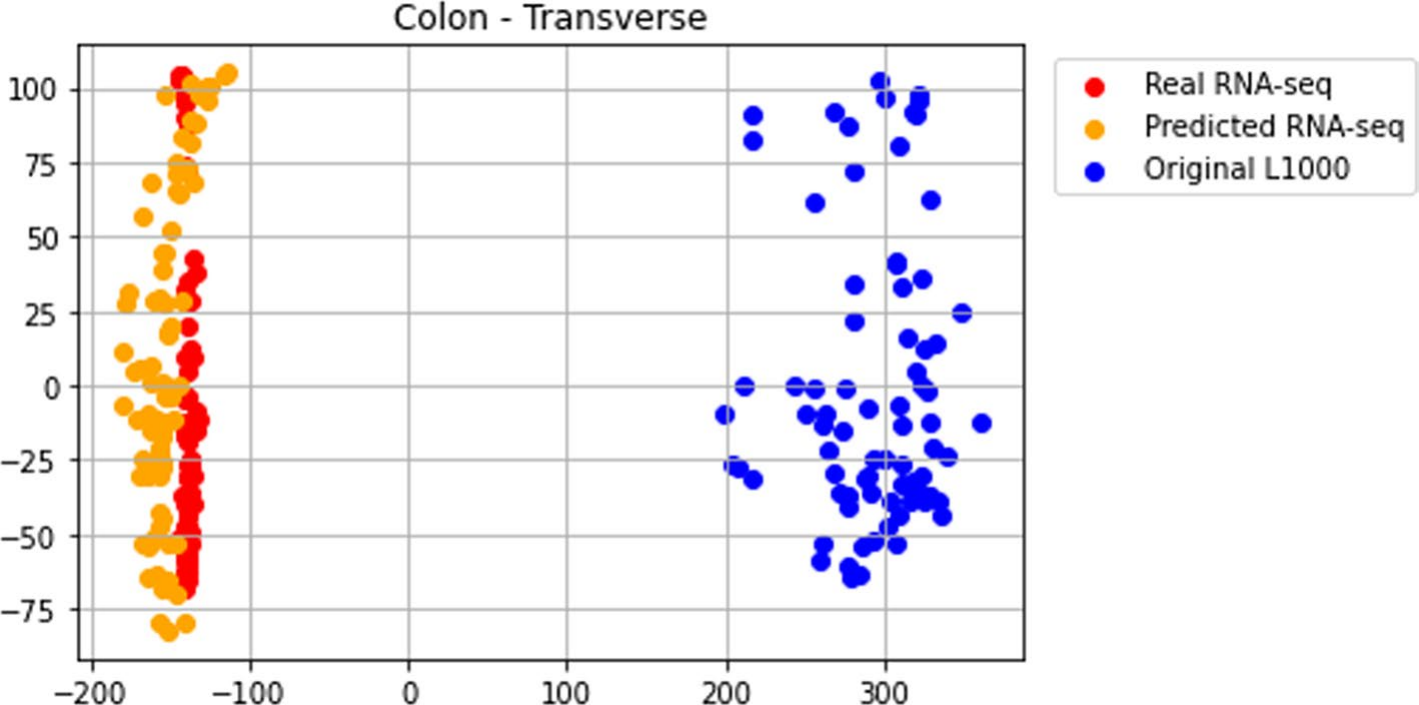
Sample visualization in reduced space. PCA plot of the real GTEx RNA-seq profiles from transverse colon (red), the predicted RNA-seq-like profiles (orange), and the original L1000 profiles (blue). The 84 samples are from post-mortem transverse colon collected for the GTEx program. The gene space is the common 11,780 genes

To demonstrate that the predicted RNA-seq-like profiles are more like the real RNA-seq profiles rather than the L1000 profiles, we visualize each sample in PCA space (Fig. 4, Additional file 1: Figs. S13–S65). These PCA plots display RNA-seq, RNA-seq-like, and L1000 samples as points in PCA space using all the common genes. We observe that the predicted RNA-seq-like profiles are closer in PCA space to the real RNA-seq profiles. Hence, the model appears to visually transform the L1000 samples to the real RNA-seq space. In addition, we divided all the GTEx samples into low, medium, and high correlation groups based on their Pearson correlation coefficients. We randomly selected one sample from the low, medium, and high correlation groups to plot these samples (Additional file 1: Fig. S66–S68). The x-axis and y-axis represent predicted RNA-seq expression values and real RNA-seq expression values, respectively; and each dot represents a gene. These scatter plots show that the predicted RNA-seq samples have high correlation with their matched predicted real values.

### Benchmarking dexamethasone signatures with ChIP-seq and RNA-seq data

**Fig. 5:**
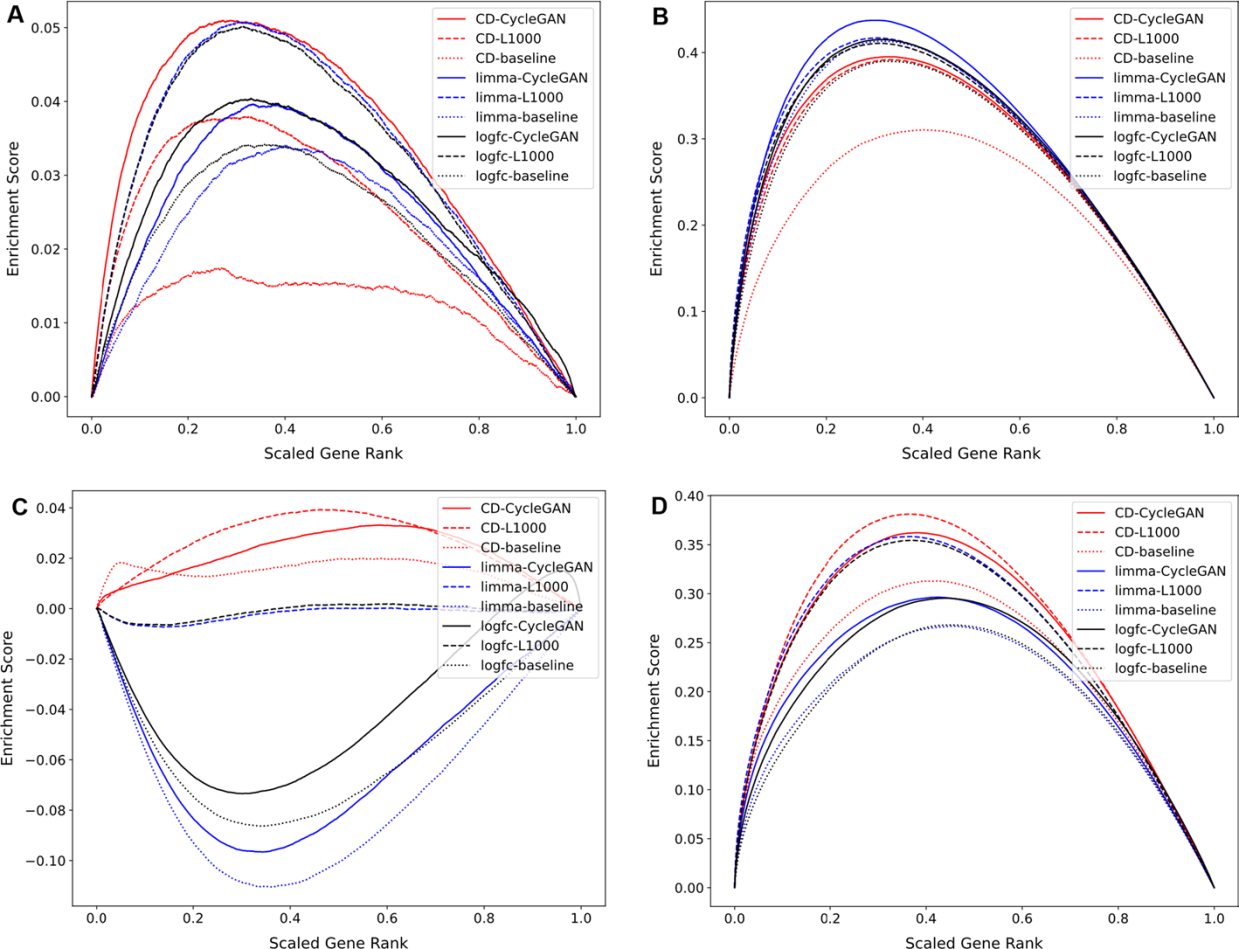
Benchmarking dexamethasone gene expression signatures with bridge plots that visualize the recovery of targets of NR3C1 as determined by ChIP-seq, given dexamethasone signatures. Signatures created from the predicted RNA-seq-like profiles are compared to the originally published L1000 signatures using only the common 11,780 genes. Unweighted walk plots comparing ranked genes from the signatures with NR3C1 target genes assembled from ChEA (**A**) and ENCODE (**C**). The same signatures are compared with weighted walks by the absolute differential expression value for each gene, normalized to fit in the range between 0 to 1 for ChIP-seq targets from (**B**) ChEA and (**D**) ENCODE

**Fig. 6:**
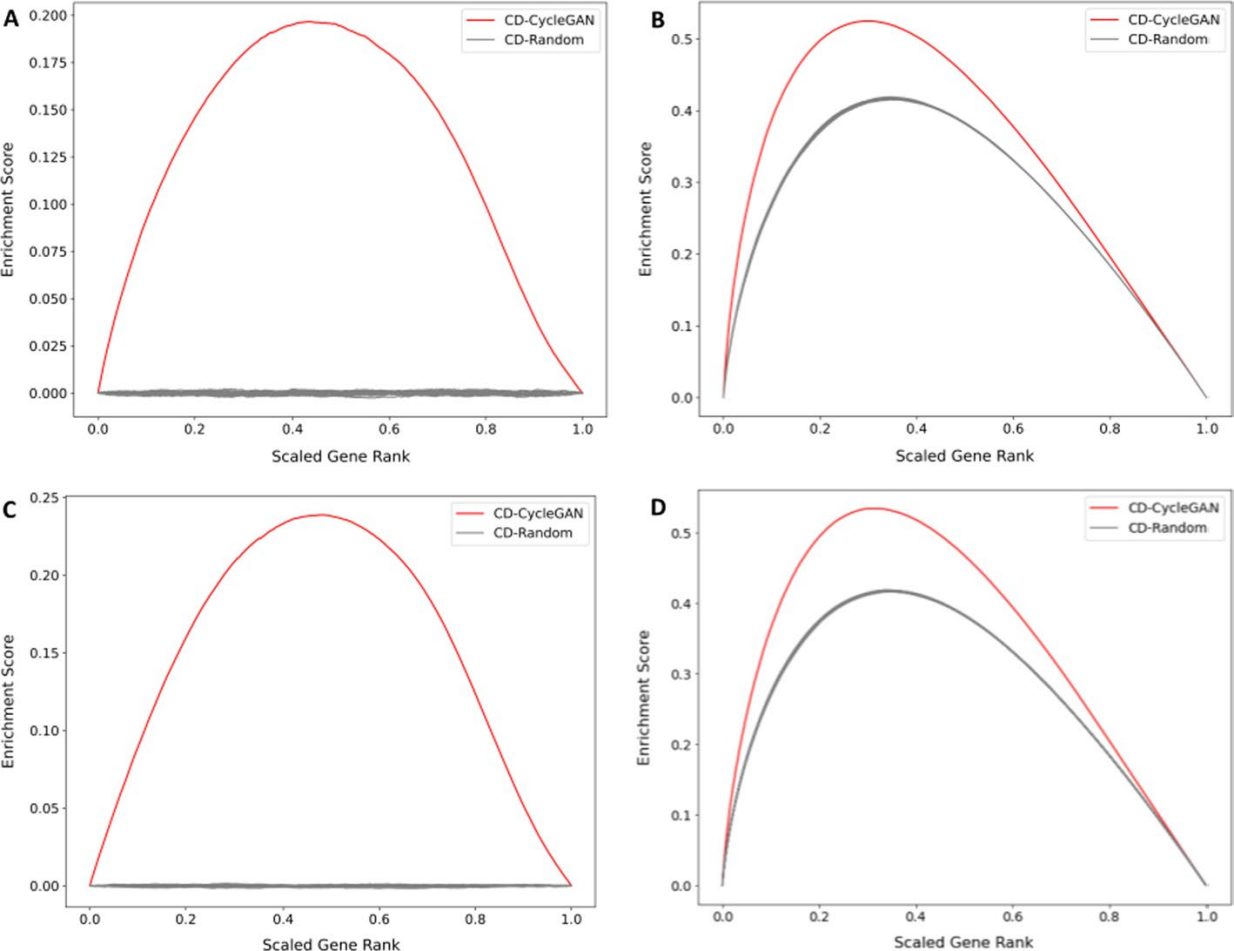
Bridge plot of dexamethasone signatures created from the predicted RNA-seq like profiles with all 23,614 genes (red) and 20 random signatures (gray) using the CD method. **A–C** Unweighted walk comparing ranked genes from the signatures with NR3C1 targets downloaded from ChEA (**A**) and ENCODE (**C**). **B–D** Weighted walk comparing the signatures weighted by the absolute value of the differential expression score for each gene, normalized to a value between 0 and 1 from NR3C1 target genes downloaded from ChEA (**B**) and ENCODE (**D**)

The most likely utility of transforming L1000 profiles into RNA-seq-like profiles is for the purpose of computing gene expression signatures and making these signatures available for search. Hence, it is critical to evaluate if gene expression signatures computed using the predicted full genome RNA-seq-like profiles can be effective for knowledge extraction. To evaluate this concept, we computed gene expression signatures of the same drug treatment, namely dexamethasone, across cell lines, concentrations, and time-points using the predicted RNA-seq-like profiles produced by the two-step model, and the predicted RNA-seq-like profiles produced by the baseline model, and the original L1000 profiles using different differential expression analysis algorithms including the characteristic direction (CD) [10], limma [11], and fold change. For each batch in the original L1000 profiles and the predicted RNA-seq-like profiles with the common 11,780 genes, samples treated with dexamethasone were used as the treatment samples and the other samples in the same batch were used as the control samples. A total of 468 signatures for dexamethasone were computed and the differentially expressed genes were ranked by the level of differential expression by each method. Then, the ranked genes from these signatures were compared to gene sets identified in independent ChIP-seq experiments as targets of the glucocorticoid receptor NR3C1. NR3C1 is the known drug target of dexamethasone. The targets of NR3C1 were collected from ChEA [12] and ENCODE [13] which are resources that contain transcription factor targets determined by ChIP-seq experiments. Bridge plots that visualize the ranks of NR3C1 ChIP-seq targets in dexamethasone signatures show that overall, the CD method outperforms the other methods by producing higher peaks (Fig. 5). The bridge plots also indicate that the predicted RNA-seq-like profiles produce comparable quality signatures to the signatures computed with the original L1000 profiles. Based on these benchmark results, we tested whether dexamethasone signatures prioritize NR3C1 ChIP-seq targets when ranking genes that are only predicted by the RNA-seq-like profiles (Fig. 6). These bridge plots show that putative targets of NR3C1, as determined by ChIP-seq experiments, that are not included within the original extrapolated L1000 profiles, are ranked much higher than expected by random chance. In addition, we generated bridge plots by computing a signature from several published dexamethasone RNA-seq studies that compared the effect of treating human cell-lines with dexamethasone. The differentially expressed signatures from these studies were compared with the RNA-seq-like signatures for dexamethasone under similar conditions (Additional file 1: Fig. S69–72). To perform this analysis, we downloaded the RNA-seq data from three previously published studies (GSE94408, GSE193988, and GSE186104) available from the Gene Expression Omnibus (GEO), and computed signatures from each study using the limma method. The bridge plots show that the signatures from the RNA-seq-like profiles produce higher peaks than other baseline models. In summary, these plots and analyses provide a silver-standard benchmark that suggest that the signatures from the predicted RNA-seq-like profiles are useful for identifying knowledge about dexamethasone targets. Although this benchmark is specific for dexamethasone, it provides confidence that the signatures created from the RNA-seq-like profiles are of good quality overall, and the predictions of the expression of the additional genes are reliable.

### Appyter to perform single gene reverse search

**Fig. 7:**
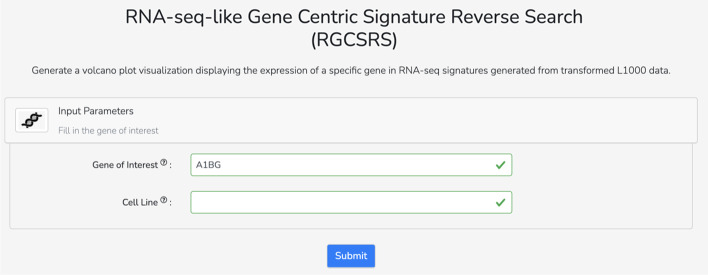
The RNA-seq-like Gene Centric Signature Reverse Search (RGCSRS) Appyter input form. The Appyter takes a query gene and a cell line and returns visualizations of the top RNA-seq signatures that up- or down-regulate the query gene

The RNA-seq-like L1000 signatures provide a rich resource for drug and target discovery. To demonstrate how this dataset can facilitate the development of tools and search engines for hypothesis generation, we developed the L1000 to RNA-seq-like Gene Centric Signature Reverse Search (RGCSRS) Appyter, available from: https://appyters.maayanlab.cloud/#/L1000_RNAseq_Gene_Search (Fig. 7). Appyters are light-weight web-based bioinformatics tools that can be generated from workflows coded as Jupyter Notebooks [14]. The RGCSRS Appyter provides single gene search against the RNA-seq-like L1000 signatures induced by CRISPR knockouts and chemical perturbations. The user submits a human gene symbol, and the results are matching signatures that maximally up- or down-regulate the expression of the queried gene. Users have the option of filtering the search by cell line. The signatures are computed from the predicted RNA-seq-like profiles by the CD method. For performance responsiveness, and to reduce computational burden, only a subset of the RNA-seq-like signatures are included for search by the RGCSRS Appyter. To generate this subset, each gene was queried against all signatures ahead of time to identify the top 100 signatures that mostly up-regulate or down-regulate the gene based on fold change, CD coefficient, and rank.

To visualize the search results, the RGCSRS Appyter generates volcano plots that display the differential expression of the query gene across the top signatures that up- or down-regulate the gene. The position of each signature on the plot is determined by the fold change (x-axis) and the CD coefficient (y-axis). The Appyter also generates downloadable tables listing the top signatures which are predicted to up- or down-regulate the query gene, along with the corresponding expression values and signature metadata. It should be noted that in rare cases, the fold change and CD coefficient of a gene are not necessarily in the same direction. This is because there are cases where a gene is both up- and down-regulated across replicate samples. The CD method prioritizes the more consistent direction of movement rather than the overall average of the movement, and thus it may not be consistent with the fold change sign.

### Case study: using the RGCSRS Appyter to prioritize small molecules that may influence the expression of novel aging targets

To demonstrate how the RGCSRS Appyter can be used for hypothesis generation, we first generated aging gene expression signatures from the GTEx v8 dataset [9]. To achieve this, we first divided the GTEx samples by primary tissue site and filtered out lowly expressed genes for each tissue type using a count per million (CPM) threshold of 10 million, divided by the median library size of the tissue samples, and a total count threshold of 15. We next grouped the samples for each tissue in the GTEx data into the provided age groups: 20–29, 30–39, 40–49, 50–59, 60–69, and 70–79. Next, we computed differentially expressed gene signatures for each age group comparing older groups to the 20–29 group for each tissue. Hence, we used the 20–29 group as the “control” group, and the other older groups as the “perturbation” groups. A total of 135 aging signatures were created. These signatures are made available for download from SigCom LINCS [15]. To minimize sample size differences, control and case samples were randomly selected for each comparison such that the number of samples representing both groups was equal. The top 250 up- and down-regulated genes from each age group comparison for each tissue were organized into a gene set library. Subsequently, we counted the most frequent 100 up- and down-regulated genes across all 135 signatures (Additional file 1: Tables S1–S2). These tables contain the number of publication (PubMed) that mentioned each gene in the title or abstract, the number of publications that also contains the terms ‘aging’ or ‘ageing’ together with the gene in the title or abstract, whether the gene is a landmark L1000 measured gene, an inferred gene by the original L1000 data, or inferred by our model. Finally, we also note whether the gene gives rise to a secreted extracellular protein. This information can assist in prioritizing the novelty of potential new targets for aging-related research, and as potential therapic targets.

**Fig. 8:**
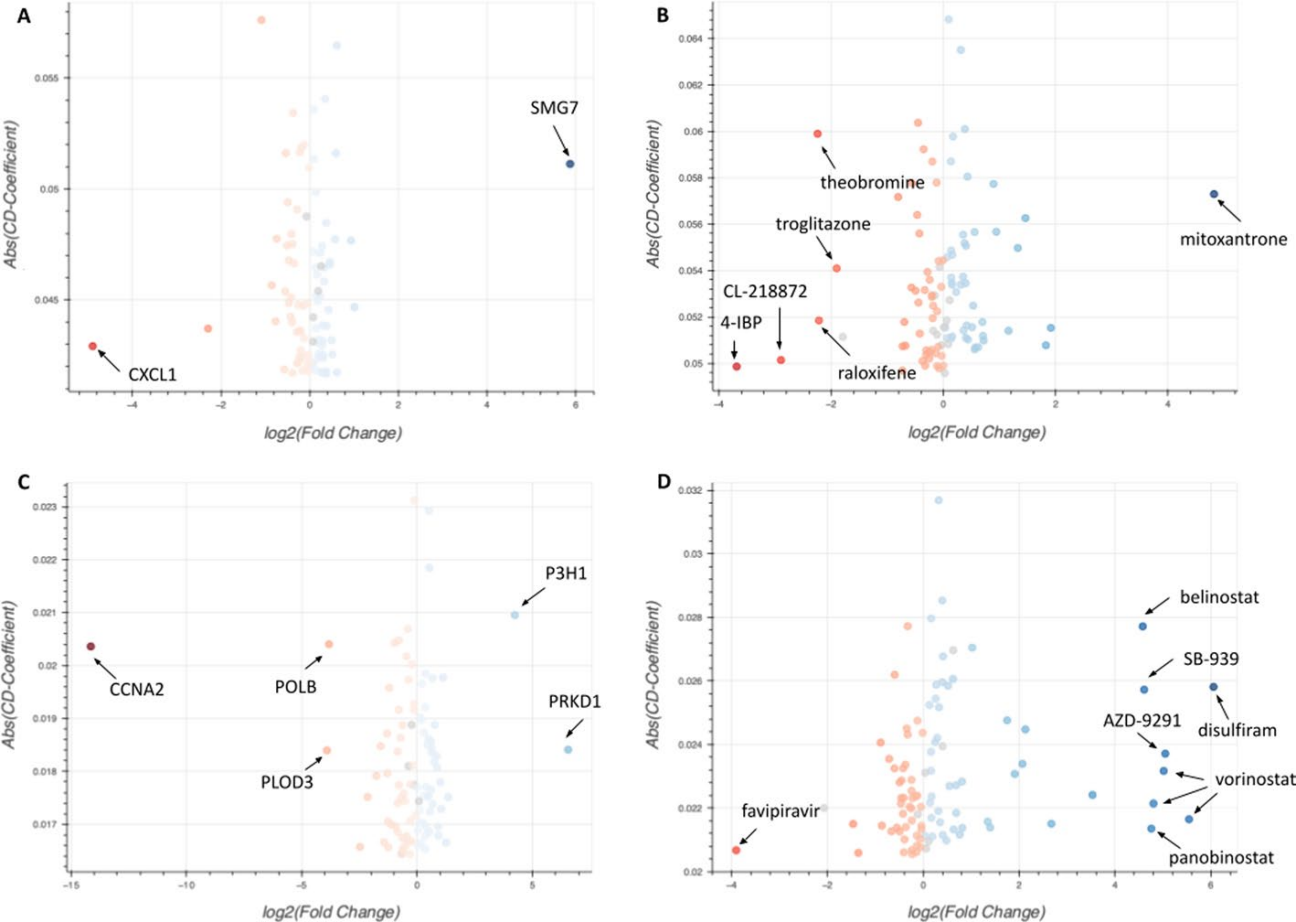
Volcano plots of signatures of CRISPR knockouts and chemical perturbagens that are predicted to up- or down-regulate SFRP2 (**A**, **B**) and LGI3 (**C**, **D**). The x-position indicates the log2(fold change) of the expression of the query gene in the CRISPR knockouts and chemical perturbagen signatures, while the y-position indicates the absolute CD coefficient of the gene. The plots highlight points with the same-direction fold change and CD coefficient values by coloring them blue (up-regulated) or red (down-regulated). Darker colored points indicate signatures where the query gene is differentially expressed with a larger absolute value of the CD-coefficient and fold change

PTCHD4 and ADIPOQ are the top up- and down-regulated genes across all aging signatures, respectively. PTCHD4 is known as a repressor of the canonical hedgehog signaling and hedgehog pathway activity is known to be decreasing with age [16]. ADIPOQ is a precursor hormone that is known to play a crucial role in protecting against insulin resistance and atherosclerosis. The decrease in ADIPOQ in aging tissues results in decrease in insulin sensitivity [17]. These genes are already known to be associated with aging and confirm that the aging signature extracted from the GTEx data is producing relevant targets. However, there are other genes that are extracellular, not inferred or measured by the original L1000 data, not previously studied in the context of aging, and not previously widely studied in general. For example, SFRP2, which is ranked 55^th^ in the list of top 100 up-regulated genes, is an under-studied secreted frizzled-related protein with little evidence about its association with aging. The RGCSRS Appyter returns CRISPR knockouts and chemical perturbations that up- or down-regulate SFRP2 (Fig. 8A, B) (https://appyters.maayanlab.cloud/L1000_RNAseq_Gene_Search/ed5ccaae36613802a251a0cf60ddd8ba32c10066/).

The top CRISPR knockouts and chemical perturbations that are identified to regulate the expression of SFRP2 include SMG7, which is known to play a role in nonsense-mediated mRNA decay (NMD). NMD is important in dietary restriction (DR)-induced longevity and SMG6 and SMG7 are required for increased life span under DR [18]. This result suggests that SMG7 knockout may down-regulate SFRP2, and this effect may promote the aging process. The CRISPR knockout that is ranked first to down-regulate the expression of SFRP2 is CXCL1. CXCL1 is a chemokine (CXC motif) ligand that plays a role in inflammation by recruiting and activating neutrophils [19]. CXCL1 is also known to play a role in senescence-associated secretory phenotypes (SASP) [20]. It is well established that senescent cells increase with aging and secrete SASP inducing chronic inflammation and age-related diseases. CXCL1 is also associated with specific aging-related diseases. For example, CXCL1 is upregulated in the aging prostate [21], it is known to inhibit muscle repair and regeneration [22], and it is implicated in Alzheimer’s disease [23, 24]. Regarding compounds predicted to maximally affect the expression of SFRP2, 4-IBP is the top-ranked chemical compound that is predicted to down-regulate SFRP2. 4-IBP is a sigma-1 receptor agonist. Sigma-1 receptor has functions in learning and memory [25] while sigma-1 receptor agonists are suggested as potential therapeutic agents against age-related cognitive deficits [25]. The second ranked compound, CL-218872 is a benzodiazepine that is a known agonist to the alpha1 subunit of GABA receptor. The alpha1 subunit of the GABA receptor is one of the most abundantly expressed proteins in the hippocampus and its mRNA expression level in the hippocampus has been reported to be significantly increased in aging [26].

Theobromine, which is ranked 3rd among the top SFRP2 down regulators, is found in the cacao plant and in chocolate. It is also a product of the metabolism of caffeine. There is increasing evidence that suggests that theobromine can promote brain health and reduce age-related neurodegenerative disorders [27]. Raloxifene which is ranked 4th as an SFRP2-down-regulator, is a selective estrogen receptor modulator used to treat osteoporosis in women who are at an increased risk of this disorder with age. In addition, there are clinical studies that suggest that raloxifene may provide benefits to treat invasive breast cancer [28], cardiovascular aging [29], and Alzheimer’s disease [30]. There is some additional independent evidence implicating SFRP2 as an up-regulated extracellular signaling component that increases in aging tissues [31], while a recent study showed that targeting SFRP2 can alleviate aging-dependent resistance to bevacizumab when treating patients against angiogenesis in melanoma [32, 33].

As another example, we investigated the leucine-rich repeat LGI family member 3 (LGI3), which is ranked 47th in the down-regulated aging genes. We found that LGI3 is significantly down-regulated in the hypothalamus and lung of aging mice across many aging related signatures extracted from GEO [34]. The RGCSRS Appyter returns CRISPR knockouts and chemical perturbations that maximally up- and down-regulate LGI3 (Fig. 8C, D). The top CRISPR knockouts and chemical perturbations for modulating LGI3 include POLB, which is ranked 3rd among the top LGI3 down-regulating CRISPR knockouts. POLB is a DNA polymerase that is involves in base excision and repair. Polymorphisms in POLB are known to be associated with longevity [35, 36]. The top ranked chemical compounds that are predicted to upregulate LGI3 include vorinostat, panobinostat, SB-939, and belinostat which are all HDAC inhibitors. HDAC inhibitors have been proposed as promising aging therapeutics by modulating epigenetic alterations observed in aging. HDAC inhibitors have demonstrated improvement in a wide range of age-related diseases such as neurodegeneration diseases, heart diseases, diabetes, and sarcopenia [37]. The top CRISPR knockout that is suggested to down-regulate LGI3 is CCNA2. It was recently reported that CCNA2 overexpression delays cellular senescence [38]. On the other hand, PRKD1 is the top CRISPR knockout that is suggested to up-regulate LGI3. PRKD1 is a protein kinase and a component of the trans-Golgi network involved in the protein secretory pathway. The level of PRKD1 is increased during cellular senescence and inhibiting this pathway decreases IL-6 and IL-8 secretion resulting in reduced ras-oncogene-induced senescence [39]. Overall, by combining aging signatures created from GTEx with the RNA-seq-like signatures we computed with the two-step model, we can obtain novel data-driven hypotheses for pre-clinical drug and target discovery.

## Discussion and conclusions

The two-step Deep Learning model presented here effectively transforms L1000 profiles to RNA-seq-like profiles. The first step of the model transforms L1000 profiles into RNA-seq-like profiles at the landmark gene space using a modified CycleGAN model. The predicted RNA-seq-like profiles from the first step are extrapolated to the full genome space using a fully connected artificial neural network model. We show that even though we do not have paired transcriptomic samples that are measured by both L1000 and RNA-seq for training, the model can transform L1000 profiles to RNA-seq-like profiles with unpaired data which are abundant with millions of samples available by either technology, namely L1000 and bulk RNA-seq. Moreover, signatures computed from RNA-seq-like profiles can be used to obtain new knowledge about currently missing protein-coding genes from the available L1000 profiles. The same approach can be extended to predict the expression of non-coding genes.

The L1000 assay was designed to measure the expression of 978 landmark genes while the expression of the rest of the genes is inferred. Currently, all published results from inference methods only provide the expression for an additional 11,350 protein-coding genes. Hence, single gene search only works on half of the human coding genes and none of the non-coding genes. Although the cost of performing RNA-seq is dropping, producing over 3M gene expression profiles with bulk RNA-seq in a uniform setting is still very costly. In addition, making L1000 into RNA-seq-like is likely better for integrating these data across these two platforms. Most individual investigators that perform gene expression analyses, use bulk or single-cell RNA-seq. Hence, it is expected that searching across all L1000 data with transformed RNA-seq-like data will produce more accurate search results.

To demonstrate the potential utility of the transformed L1000 signatures, we developed an Appyter that can predict drugs and single gene perturbations that are likely to up- or down-regulate the expression of a target gene. By querying the RGCSRS Appyter with the genes SFRP2 and LGI3, two genes that are consistently differentially expressed across aging tissues, we noticed that drugs that are already known to impact aging processes are highly prioritized. Other less studied small molecules and genetic perturbations are also highly ranked and could be tested for their effects on aging processes. Alternatively and more directly, the protein products of SFRP2 and LGI3 could be targeted by antibodies, or introduced as recombinant proteins or mRNA vectors, to examine their effect on aging processes. This case study and the selected highlighted genes are provided as an example, opening the door to many other applications for illuminating other biological and pharmacological contexts.

However, users of the RNA-seq-like transformed data should be aware of the limitations of such data. For example, the predicted RNA-seq-like profiles poorly predict the expression of the targeted single genes from the shRNA and CRISPR knockdown profiles. Expression of the perturbed knocked-down or knocked-out gene in the shRNA or CRISPR profiles is expected to be lower than its expression in control profiles. Our analysis suggest that the two-step Deep Learning model can predict well how knockdown- or knockout-genes may affect the global overall expression profile while incapable of predicting well the expression of a particular single perturbed gene.

The overall approach of transforming one dataset into another domain can be expanded to other applications. For example, predicting the expression and function of long non-coding RNAs (lncRNAs) or converting expression profiles collected with microarrays to become RNA-seq-like. Particularly, prioritizing small molecules and single gene perturbations that are likely to modulate the expression of lncRNAs can illuminate the role of these understudied genes. Moreover, the translation from one omics profiling technology to another can also motivate translation between other multi-omics datasets. Transforming microarrays to RNA-seq, transcriptomics to proteomics, genomics to transcriptomics, single cell RNA-seq to bulk RNA-seq and vice versa, and microscopy imaging to omics, or omics to microscopy images even when there are no matching paired samples.

## Methods

### Data preparation

To train the model, we first prepared unpaired L1000 profiles and bulk RNA-seq profiles for training the CycleGAN component of the two-step model. L1000 profiles were downloaded from the Gene Expression Omnibus (GEO) [40] (GSE92742) and 50,000 samples were randomly selected for further processing. At the same time, 50,000 randomly selected human RNA-seq samples were obtained from ARCHS4 [41], a resource that provides over 1 million uniformly aligned RNA-seq samples from GEO. To filter out lowly expressed genes from the RNA-seq samples, only genes with read counts of at least 10 in 2% of the samples were retained. The gene counts were then log2 transformed and quantile normalized.

### First step model

The first step of the translator model utilizes a modified CycleGAN [3] to convert gene expression values in L1000 978 landmark space to RNA-seq-like profiles in the same space. The model contains two generators. One for generating RNA-seq-like profiles from L1000 profiles (G), and one for generating L1000-like profiles from RNA-seq profiles (F). The first generator takes L1000 profiles as input, and outputs RNA-seq-like profiles. The RNA-seq output by the first generator is used as input to the second generator, and the output of the second generator is expected to match the original L1000 profiles. Discriminators (${D}_{X}$, ${D}_{Y}$) are implemented to determine how plausible are the generated RNA-seq profiles and L1000 profiles to update the generator models. We apply adversarial losses, cycle consistency loss, and identity loss based on the Mean Squared Error (MSE) to evaluate the level of training over time. For the L1000 to RNA-seq mapping function G: X → Y and its discriminator ${D}_{Y}$ there are two domains: X (L1000) and Y (RNA-seq); Hence, the adversarial loss function is:

$${\mathcal{L}}_{\mathcal{G}\mathcal{A}\mathcal{N}}\left(G,{D}_{Y},X,Y\right)={E}_{y\sim {p}_{dat{a}_{\left(y\right)}}}{\left[D\left(y\right)\right]}^{2}+{E}_{x\sim {p}_{data}\left(x\right)}{\left[1-D\left(G\left(x\right)\right)\right]}^{2}$$

where G is a generator that generates RNA-seq profiles given L1000 profiles, while D_y_ aims to distinguish between real RNA-seq profiles and generated RNA-seq profiles.

For the RNA-seq to L1000 mapping function F: Y → X and its discriminator ${D}_{X}$, we apply a similar loss function: ${\mathcal{L}}_{\mathcal{G}\mathcal{A}\mathcal{N}}\left(F,{D}_{X},Y,X\right)$. We also use the cyclic consistency loss to convert transformed L1000 data back into the original L1000 and vice versa.

$${\mathcal{L}}_{\mathcalligra{cyc}}\left(G,F\right)={E}_{x\sim {p}_{dat{a}_{\left(X\right)}}}{\left[F\left(G\left(x\right)\right)-x\right]}^{2}+{E}_{y\sim {p}_{dat{a}_{\left(Y\right)}}}{\left[G\left(F\left(y\right)\right)-y\right]}^{2}$$

The original CycleGAN implementation introduces an identity loss function [42] for transforming paintings to photos to preserve color composition between the input and output. We also apply an identity loss function to preserve the common gene expression patterns in profiles regardless of assay platform. The identity loss function is expressed as follows:

$${\mathcal{L}}_{\mathcalligra{identity}}\left(G,F\right)={E}_{x\sim {p}_{dat{a}_{\left(X\right)}}}{\left[F\left(x\right)-x\right]}^{2}+{E}_{y\sim {p}_{dat{a}_{\left(Y\right)}}}{\left[G\left(y\right)-y\right]}^{2}$$

The final objective is then:

$$\mathcal{L}\left(G,F,{D}_{X},{D}_{Y}\right)={L}_{GAN}\left(G,{D}_{Y},X,Y\right)+{L}_{GAN}\left(F,{D}_{X},Y,X\right)+{\uplambda }_{cyc}*{L}_{cyc}\left(G,F\right)+{\uplambda }_{identity}*{L}_{identity}\left(G,F\right)$$

where ${\lambda }_{cyc}$ and ${\lambda }_{identity}$ give relative importance to the cycle consistency loss and the identity loss. Here, ${\lambda }_{cyc}$ and ${\lambda }_{identity}$ are set to 10. Finally, the model is optimized to satisfy:

$$mi{n}_{G,F}ma{x}_{{D}_{X},{D}_{Y}\mathcal{L}}\left(G,F,{D}_{X},{D}_{Y}\right).$$

G, F, ${D}_{X}$ and ${D}_{Y}$ are two-layered fully connected neural networks. A learning rate was set to 0.0002. The optimal number of layers, a learning rate, ${\lambda }_{cyc}$ and ${\lambda }_{identity}$ are selected based on prediction results on MCF7 DMSO samples. The most critical hyper-parameters for the model performance are ${\lambda }_{cyc}$ and ${\lambda }_{identity}$. Adam optimizer [43] was adopted as the optimization method, and the activation function for each layer in the generators and discriminators is a Rectified Linear Unit (ReLU). The model is trained for 100 epochs. To avoid generating negative values in the output, we use ReLU for the last layer of G and F.

### Second step model

For the second step of the translator model, we trained a fully connected neural network that takes the output profiles from the first step to predict the expression levels of 23,614 genes. The model was trained with 50,000 randomly selected RNA-seq profiles from ARCHS4. Among these 23,614 genes, profiles of the landmark genes were used as input, and the rest of the genes were used as the target values. The model architecture has 3 layers with each layer having: 962, 2048, 8162, 23,614 units. The model uses the ReLU activation function. The Adam optimizer was used to optimize the model with a learning rate of 2e-4 and batch size of 100. A validation set is used for hyper-parameter optimization and early stopping with patience of 3 epochs. To avoid outputting negative values, ReLU is applied to the output of the model. The two-step model is implemented in Python 3 using PyTorch [44]. Training the whole pipeline takes about two hours to complete on a state-of-the-art desktop computer. Using the trained model, it takes approximately 2 min to predict the full expression profiles for 10,000 L1000 samples using a standard state-of-the-art desktop computer.

## Supplementary Information

**Additional file 1. **The file contains Tables S1–S2 and Figures S1–S72.

## Data Availability

All transformed L1000 data in RNA-seq-like format is available for download from https://maayanlab.cloud/sigcom-lincs/#/Download under the subheadings “CycleGAN Predicted RNA-Seq-Like Profiles of L1000 Samples (Level 3)” and “Computed Signatures for CycleGAN Predicted RNA-Seq-Like Profiles of L1000 Samples (Level 5)”. All the code written for the project is available from a GitHub repository here: https://github.com/MaayanLab/L1000toRNAseq.
