# Supplementary material for: Transforming L1000 profiles to RNA-seq-like profiles with deep learning
Source: BMC Bioinformatics. 2022 Sep 13;23:374. doi: 10.1186/s12859-022-04895-5 (PMC9472394; doi:10.1186/s12859-022-04895-5)
Supplement: Supplementary file 1 — Additional file 1. The file contains Tables S1–S2 and Figures S1–S72. [file 12859_2022_4895_MOESM1_ESM.pdf]

Rank	Gene	Publication with aging/ageing	Publication	Landmark	Inferred by L1000	Inferred by CycleGAN	Secreted	Counts in GTEx Up Signatures	Counts in GTEx Down signatures
1	PTCHD4	0	2	FALSE	FALSE	TRUE	FALSE	88	0
2	PURPL	0	5	FALSE	FALSE	FALSE	FALSE	80	0
3	MTHFD2P1	0	0	FALSE	FALSE	TRUE	FALSE	57	0
4	ABCB5	1	216	FALSE	FALSE	TRUE	FALSE	54	3
5	HBA1	8	1855	FALSE	FALSE	TRUE	FALSE	52	3
6	HBB	10	1705	FALSE	TRUE	TRUE	FALSE	52	1
7	HBD	12	2480	FALSE	TRUE	TRUE	FALSE	52	8
8	HBA2	0	829	FALSE	FALSE	TRUE	FALSE	51	1
9	EDA2R	2	39	FALSE	TRUE	TRUE	FALSE	48	0
10	LINC01291	0	3	FALSE	FALSE	FALSE	FALSE	47	9
11	DUXAP8	0	46	FALSE	FALSE	TRUE	FALSE	47	0
12	GDF15	51	828	FALSE	TRUE	TRUE	TRUE	47	1
13	LMO3	0	64	FALSE	TRUE	TRUE	FALSE	46	0
14	CDKN2A	123	4980	TRUE	FALSE	TRUE	FALSE	43	0
15	SLC6A15	1	39	FALSE	TRUE	TRUE	FALSE	42	5
16	COMP	40	4169	FALSE	TRUE	TRUE	TRUE	41	4
17	GPR15	0	134	FALSE	TRUE	TRUE	FALSE	41	1
18	JCHAIN	0	18	FALSE	TRUE	TRUE	TRUE	39	5
19	PYHIN1	0	15	FALSE	TRUE	TRUE	FALSE	39	0
20	FGA	13	1428	FALSE	TRUE	TRUE	TRUE	37	5
21	EYA4	1	113	FALSE	TRUE	TRUE	FALSE	37	0
22	PNLIP	1	43	FALSE	TRUE	FALSE	TRUE	36	16
23	FGB	2	415	FALSE	TRUE	TRUE	TRUE	36	6
24	CXCL10	66	5427	FALSE	TRUE	TRUE	TRUE	35	3
25	ALAS2	2	305	FALSE	TRUE	TRUE	FALSE	34	3
26	CXCL11	6	1159	FALSE	TRUE	TRUE	TRUE	33	5
27	PRSS1	0	384	FALSE	TRUE	TRUE	TRUE	32	16
28	FGG	5	555	FALSE	TRUE	TRUE	TRUE	32	8
29	RPL13AP12	0	0	FALSE	FALSE	FALSE	FALSE	32	0
30	CELA3A	0	7	FALSE	TRUE	TRUE	FALSE	31	15
31	SHOX2	2	193	FALSE	TRUE	TRUE	FALSE	31	3
32	LNCTAM34A	0	1	FALSE	FALSE	FALSE	FALSE	31	0
33	IFNG	11	1685	FALSE	TRUE	TRUE	TRUE	31	4
34	UNC5B-AS1	0	9	FALSE	FALSE	FALSE	FALSE	30	1
35	CACNA2D3-AS1	0	1	FALSE	FALSE	FALSE	FALSE	30	0
36	LOC105375166	0	0	FALSE	FALSE	FALSE	FALSE	30	0
37	SNORD14E	0	2	FALSE	FALSE	FALSE	FALSE	30	1
38	CLPS	1	583	FALSE	TRUE	TRUE	TRUE	29	15
39	SULT1C2	0	39	FALSE	TRUE	TRUE	FALSE	29	1

40	FMO3	8	473	FALSE	TRUE	TRUE	FALSE	29	0
41	CCL19	7	1192	FALSE	TRUE	TRUE	TRUE	29	4
42	SLC4A1	0	275	FALSE	TRUE	TRUE	FALSE	29	3
43	PRSS2	0	68	FALSE	FALSE	TRUE	TRUE	28	21
44	SLN	19	6525	FALSE	TRUE	TRUE	FALSE	28	5
45	CRP	767	55151	FALSE	TRUE	TRUE	TRUE	27	6
46	FAM133A	0	7	FALSE	FALSE	TRUE	FALSE	27	0
47	MUC7	1	225	FALSE	TRUE	TRUE	TRUE	27	20
48	HBM	26	1953	FALSE	FALSE	TRUE	FALSE	27	3
49	CD70	7	722	FALSE	TRUE	TRUE	FALSE	27	2
50	CTRB2	0	15	FALSE	TRUE	TRUE	TRUE	26	13
51	POU3F4	1	121	FALSE	TRUE	TRUE	FALSE	26	1
52	H2BC18	0	0	FALSE	FALSE	FALSE	FALSE	26	1
53	ADCYAP1	1	141	FALSE	TRUE	TRUE	TRUE	26	3
54	SLC14A1	0	75	FALSE	TRUE	TRUE	FALSE	26	2
55	SFRP2	13	571	FALSE	FALSE	TRUE	TRUE	26	1
56	SAA2	0	276	FALSE	FALSE	TRUE	TRUE	25	2
57	CP	355	61173	FALSE	TRUE	TRUE	TRUE	25	0
58	TRDN	0	46	FALSE	TRUE	TRUE	FALSE	25	2
59	CYP4F60P	0	0	FALSE	FALSE	TRUE	FALSE	25	5
60	MAS1L	0	3	FALSE	FALSE	FALSE	FALSE	25	2
61	VN1R85P	0	0	FALSE	FALSE	TRUE	FALSE	25	0
62	MUC19	1	61	FALSE	FALSE	FALSE	TRUE	25	0
63	GP2	4	767	FALSE	TRUE	TRUE	TRUE	24	20
64	CNGA3	0	232	FALSE	TRUE	TRUE	FALSE	24	1
65	NXF3	1	22	FALSE	TRUE	TRUE	FALSE	24	0
66	ANGPTL5	0	17	FALSE	FALSE	TRUE	TRUE	24	0
67	CXCL9	25	2327	FALSE	TRUE	TRUE	TRUE	24	3
68	TRAT1	0	8	FALSE	TRUE	TRUE	FALSE	24	1
69	TBC1D3L	0	0	FALSE	FALSE	TRUE	FALSE	24	3
70	CHI3L1	17	597	FALSE	TRUE	TRUE	TRUE	24	13
71	GZMH	1	29	FALSE	TRUE	TRUE	FALSE	24	0
72	EPHA6	0	63	FALSE	FALSE	TRUE	FALSE	24	1
73	GPR174	0	31	FALSE	FALSE	TRUE	FALSE	24	1
74	CELA3B	0	15	FALSE	TRUE	TRUE	FALSE	23	11
75	SUPT20HL1	0	1	FALSE	FALSE	FALSE	FALSE	23	1
76	ADGRG4	0	3	FALSE	FALSE	TRUE	FALSE	23	4
77	CTRB1	0	32	FALSE	FALSE	TRUE	TRUE	23	14
78	MUC16	0	460	FALSE	TRUE	TRUE	TRUE	23	3
79	CTD-3080P12.3	0	1	FALSE	FALSE	FALSE	FALSE	23	5
80	PCDHA2	0	2	FALSE	TRUE	TRUE	FALSE	23	0
81	AHSP	2	126	FALSE	TRUE	TRUE	FALSE	23	1

82	SERTM2	0	0	FALSE	FALSE	FALSE	FALSE	23	7
83	LOC339260	0	1	FALSE	FALSE	FALSE	FALSE	23	0
84	CD69	52	5292	FALSE	TRUE	TRUE	FALSE	23	3
85	PTPRQ	0	45	FALSE	FALSE	TRUE	FALSE	22	0
86	MTND1P23	0	0	FALSE	FALSE	TRUE	FALSE	22	8
87	REG1A	0	56	FALSE	TRUE	TRUE	TRUE	22	18
88	LOC100419679	0	0	FALSE	FALSE	FALSE	FALSE	22	0
89	FCRL5	2	51	FALSE	FALSE	TRUE	FALSE	22	8
90	H2BU1	0	0	FALSE	FALSE	FALSE	FALSE	22	1
91	ADH4	1	188	FALSE	FALSE	TRUE	FALSE	22	3
92	ALX1	0	91	FALSE	TRUE	TRUE	FALSE	22	0
93	CDH10	0	35	FALSE	TRUE	TRUE	FALSE	22	1
94	SH2D1A	1	260	FALSE	TRUE	TRUE	FALSE	22	3
95	PRKY	0	23	FALSE	TRUE	TRUE	FALSE	22	12
96	MS4A2	1	54	FALSE	TRUE	TRUE	FALSE	22	1
97	RPSAP36	0	0	FALSE	FALSE	TRUE	FALSE	22	0
98	DTHD1	0	6	FALSE	FALSE	TRUE	FALSE	22	4
99	KDM5D	0	50	FALSE	TRUE	TRUE	FALSE	22	15
100	APOBEC3H	0	98	FALSE	FALSE	TRUE	FALSE	22	0

Table S1. Top 100 consensus up-regulated genes in the GTEx aging library. The genes are sorted by frequency across the GTEx down signatures. The table contains the number of publications for each gene in the title or abstract, the number of publications that contains the gene and the term ‘aging’ or ‘ageing’ in the title or abstract, whether the gene is a landmark gene, an inferred gene by L1000, or an inferred gene by our model, and whether it is extracellular.

Rank	Gene	Publication with aging/ageing	Publication	Landmark	Inferred by L1000	Inferred by CycleGAN	Secreted	Counts in GTEx Up Signatures	Counts in GTEx Down signatures
1	ADIPOQ	10	1131	FALSE	TRUE	TRUE	TRUE	6	44
2	ECEL1	0	43	FALSE	TRUE	TRUE	FALSE	0	39
3	MMP3	38	1700	FALSE	TRUE	TRUE	TRUE	5	37
4	GSTM1	36	3998	FALSE	TRUE	TRUE	FALSE	16	35
5	RNF17	0	21	FALSE	TRUE	TRUE	FALSE	1	33
6	CIDEC	6	175	FALSE	TRUE	TRUE	FALSE	5	30
7	NWD2	0	9	FALSE	FALSE	TRUE	FALSE	2	30
8	PI15	0	24	FALSE	TRUE	TRUE	TRUE	2	30
9	CD177	2	169	FALSE	TRUE	TRUE	TRUE	2	30
10	XIST	7	1685	FALSE	TRUE	FALSE	FALSE	15	29
11	CCL22	4	1125	FALSE	TRUE	TRUE	TRUE	6	28
12	PRL	206	17073	FALSE	TRUE	TRUE	TRUE	17	28
13	REG1B	1	25	FALSE	TRUE	TRUE	TRUE	15	28
14	CCL7	11	597	FALSE	TRUE	TRUE	TRUE	1	28
15	RNASE2	0	35	FALSE	TRUE	TRUE	FALSE	7	27
16	CCL20	10	1904	FALSE	TRUE	TRUE	TRUE	11	27
17	IL1R2	4	222	FALSE	TRUE	TRUE	TRUE	1	26
18	SIK1	0	163	FALSE	TRUE	TRUE	FALSE	7	26
19	CIDEA	4	324	FALSE	TRUE	TRUE	FALSE	3	25
20	CALCA	3	169	FALSE	TRUE	TRUE	TRUE	9	25
21	PTX3	13	1354	FALSE	TRUE	TRUE	TRUE	2	25
22	ZFP57	3	112	FALSE	FALSE	TRUE	FALSE	11	25
23	MT1A	8	156	FALSE	FALSE	TRUE	FALSE	5	25
24	GPS2P1	0	1	FALSE	FALSE	TRUE	FALSE	5	25
25	NLRP2	3	96	FALSE	TRUE	TRUE	FALSE	0	24
26	LUCAT1	0	86	FALSE	FALSE	FALSE	FALSE	1	24
27	KCNS1	1	23	FALSE	TRUE	TRUE	FALSE	1	24
28	LOC102724594	0	0	FALSE	FALSE	FALSE	FALSE	4	24
29	PCK1	8	517	FALSE	TRUE	TRUE	FALSE	10	23
30	KLK3	0	310	FALSE	TRUE	TRUE	TRUE	4	23
31	CEACAM5	0	191	FALSE	TRUE	TRUE	FALSE	5	23
32	EDN3	1	211	FALSE	TRUE	TRUE	TRUE	0	22
33	FOSB	11	1168	FALSE	TRUE	TRUE	FALSE	16	22
34	MMP1	71	1864	TRUE	FALSE	TRUE	TRUE	4	22
35	TRARG1	0	1	FALSE	FALSE	FALSE	FALSE	1	22
36	HTR3A	1	168	FALSE	TRUE	TRUE	FALSE	0	22
37	UNC5D	0	42	FALSE	FALSE	TRUE	FALSE	8	22
38	DMBT1	2	268	FALSE	TRUE	TRUE	TRUE	13	22

39	CSF3	3	246	FALSE	TRUE	TRUE	TRUE	15	22
40	SERPINA3	7	185	FALSE	TRUE	TRUE	TRUE	11	21
41	TMPRSS2	18	2090	FALSE	TRUE	TRUE	TRUE	4	21
42	PRSS2	0	68	FALSE	FALSE	TRUE	TRUE	28	21
43	REG3A	0	83	FALSE	TRUE	TRUE	TRUE	8	21
44	OLAH	0	57	FALSE	TRUE	TRUE	FALSE	3	21
45	SOHLH2	0	59	FALSE	TRUE	TRUE	FALSE	2	21
46	SLC26A3	0	239	FALSE	TRUE	TRUE	FALSE	3	21
47	LGI3	0	31	FALSE	FALSE	TRUE	TRUE	0	21
48	LGALS12	0	11	FALSE	FALSE	TRUE	FALSE	5	21
49	ADGRG7	0	5	FALSE	FALSE	TRUE	FALSE	8	21
50	MUC7	1	225	FALSE	TRUE	TRUE	TRUE	27	20
51	CRNN	1	66	FALSE	TRUE	TRUE	FALSE	1	20
52	NPPC	1	165	FALSE	TRUE	TRUE	TRUE	1	20
53	CWH43	0	17	FALSE	TRUE	TRUE	FALSE	3	20
54	LAD1	0	95	FALSE	TRUE	TRUE	TRUE	3	20
55	GP2	4	767	FALSE	TRUE	TRUE	TRUE	24	20
56	MOGAT2	0	20	FALSE	TRUE	TRUE	FALSE	8	20
57	NEFH	1	107	FALSE	TRUE	TRUE	FALSE	0	20
58	CAMP	658	88758	FALSE	TRUE	TRUE	TRUE	7	20
59	SELE	3	226	FALSE	TRUE	TRUE	FALSE	5	20
60	SULT1B1	0	83	FALSE	TRUE	TRUE	FALSE	3	20
61	SERPINB2	5	177	FALSE	TRUE	TRUE	TRUE	3	20
62	PGC	439	8352	FALSE	TRUE	TRUE	TRUE	13	20
63	DGAT2	3	513	FALSE	FALSE	TRUE	FALSE	0	20
64	DLGAP5	1	83	FALSE	TRUE	TRUE	FALSE	2	20
65	CCK	139	23177	FALSE	TRUE	TRUE	TRUE	1	20
66	HMGCS2	5	211	FALSE	TRUE	TRUE	FALSE	10	19
67	USP32P1	0	1	FALSE	FALSE	TRUE	FALSE	7	19
68	ZG16	0	28	FALSE	TRUE	TRUE	TRUE	3	19
69	DHRS9	1	40	FALSE	TRUE	TRUE	FALSE	0	19
70	SLC9A2	0	23	FALSE	TRUE	TRUE	FALSE	3	19
71	CHP2	0	55	FALSE	TRUE	TRUE	FALSE	4	19
72	SLC7A10	0	26	FALSE	TRUE	TRUE	FALSE	4	19
73	WIF1	6	357	FALSE	TRUE	TRUE	TRUE	6	19
74	ESRP1	0	156	FALSE	TRUE	TRUE	FALSE	5	18
75	SLC27A2	0	51	FALSE	TRUE	TRUE	FALSE	0	18
76	KRT78	0	7	FALSE	FALSE	TRUE	FALSE	3	18
77	CCDC144A	0	0	FALSE	TRUE	TRUE	FALSE	1	18
78	PRM1	2	320	FALSE	TRUE	FALSE	FALSE	9	18
79	KRT20	0	75	FALSE	TRUE	TRUE	FALSE	2	18
80	REG1A	0	56	FALSE	TRUE	TRUE	TRUE	22	18

81	CR2	5	2010	FALSE	TRUE	TRUE	FALSE	4	18
82	S100A12	3	655	FALSE	TRUE	TRUE	TRUE	6	18
83	RXFP2	4	189	FALSE	FALSE	TRUE	FALSE	9	18
84	IGF2BP3	2	225	FALSE	TRUE	TRUE	FALSE	0	18
85	METTL7B	0	22	FALSE	FALSE	TRUE	FALSE	4	18
86	S100A9	17	1609	FALSE	TRUE	TRUE	TRUE	1	18
87	OLFM4	3	219	FALSE	TRUE	TRUE	TRUE	5	18
88	SLC6A14	0	100	FALSE	TRUE	TRUE	FALSE	3	18
89	SOWAHB	0	2	FALSE	FALSE	TRUE	FALSE	4	18
90	MCEMP1	0	11	FALSE	FALSE	TRUE	FALSE	5	18
91	NTSR2	0	50	FALSE	TRUE	TRUE	FALSE	1	18
92	PIGR	9	601	FALSE	TRUE	TRUE	TRUE	11	18
93	AGR2	4	341	FALSE	TRUE	TRUE	TRUE	7	18
94	CA4	62	1637	FALSE	TRUE	TRUE	FALSE	1	18
95	FOSL1	4	282	TRUE	FALSE	TRUE	FALSE	1	18
96	SLC38A8	0	17	FALSE	FALSE	TRUE	FALSE	3	18
97	GJB1	0	305	FALSE	TRUE	TRUE	FALSE	7	17
98	CD1E	0	68	FALSE	TRUE	TRUE	FALSE	14	17
99	CYP4F22	0	38	FALSE	FALSE	TRUE	FALSE	4	17
100	IHH	12	1534	FALSE	TRUE	TRUE	TRUE	2	17

Table S2. Top 100 consensus down-regulated genes in the GTEx aging library. The genes are sorted by frequency across the GTEx down signatures. The table contains the number of publications for each gene in the title or abstract, the number of publications that contains the gene and the term ‘aging’ or ‘ageing’ in the title or abstract, whether the gene is a landmark gene, an inferred gene by L1000, or an inferred gene by our model, and whether it is extracellular.

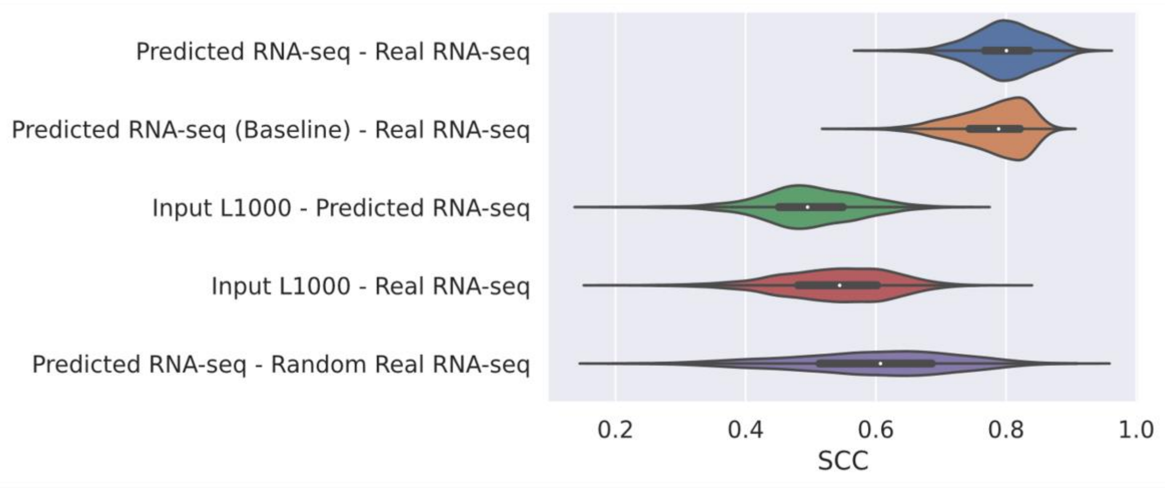

Comparing similarity in SCC between predicted and real profiles at the 978-landmark space

Fig. S1

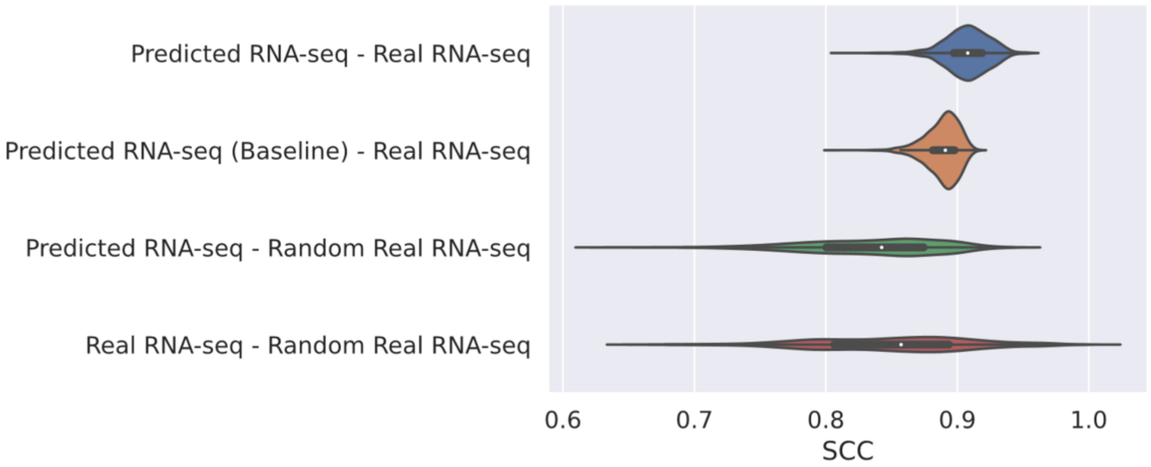

Comparing similarity in SCC between predicted and real profiles at the full genome space

Fig. S2

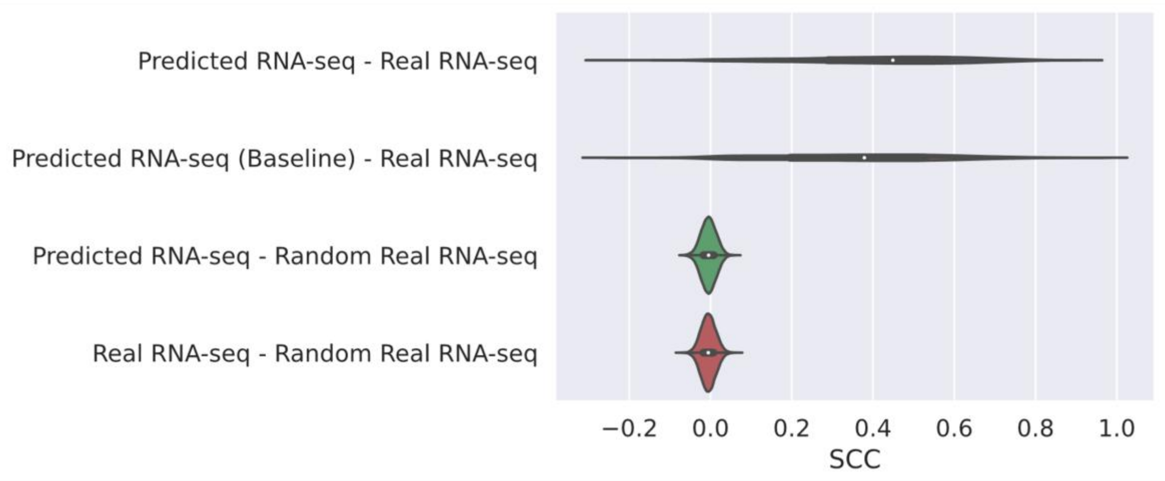

Comparing similarity in SCC between predicted and real profiles at the gene level at the full genome space

Fig. S3

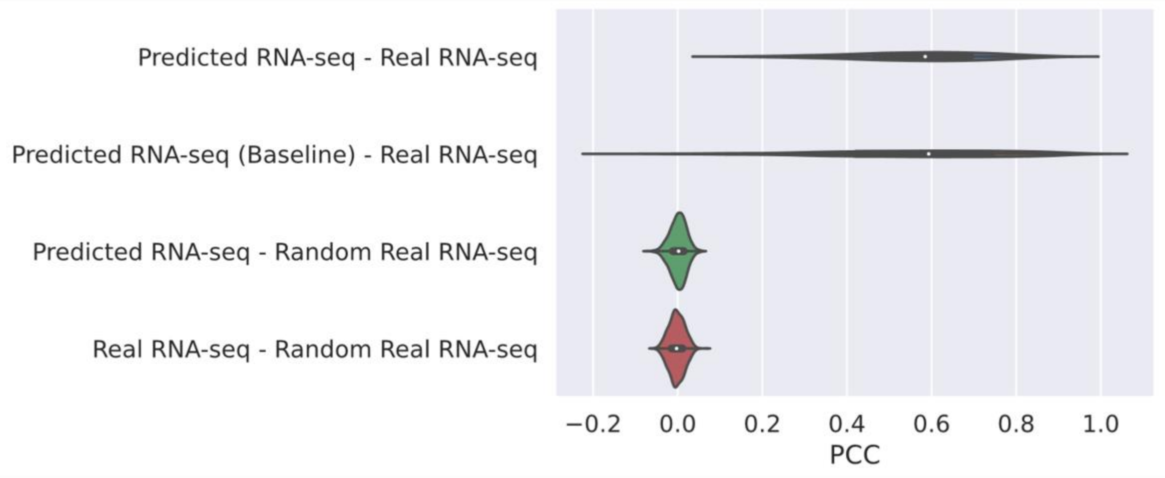

Comparing similarity in PCC between predicted and real profiles at the gene level at the 978-landmark space

Fig. S4

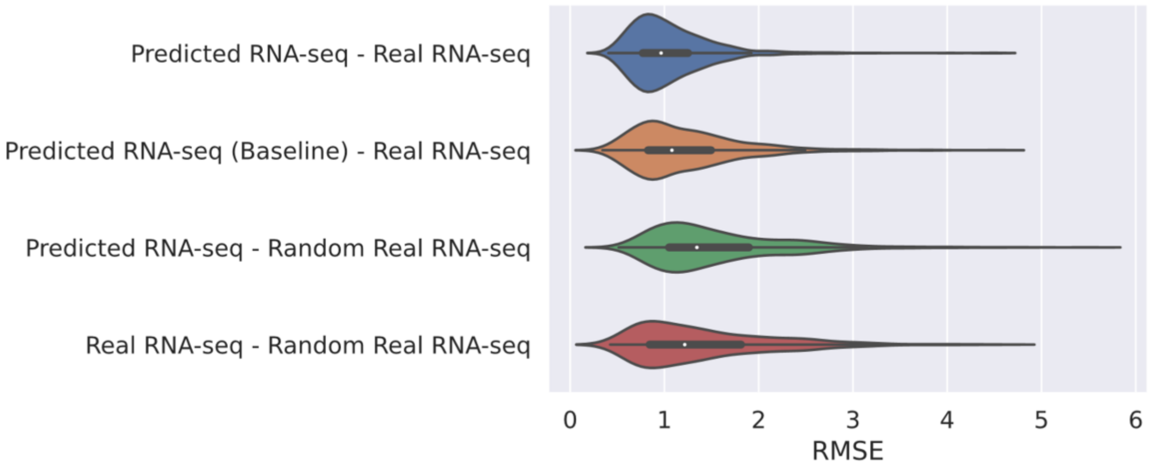

Comparing similarity in RMSE between predicted and real profiles at the gene level at the 978-landmark space

Fig. S5

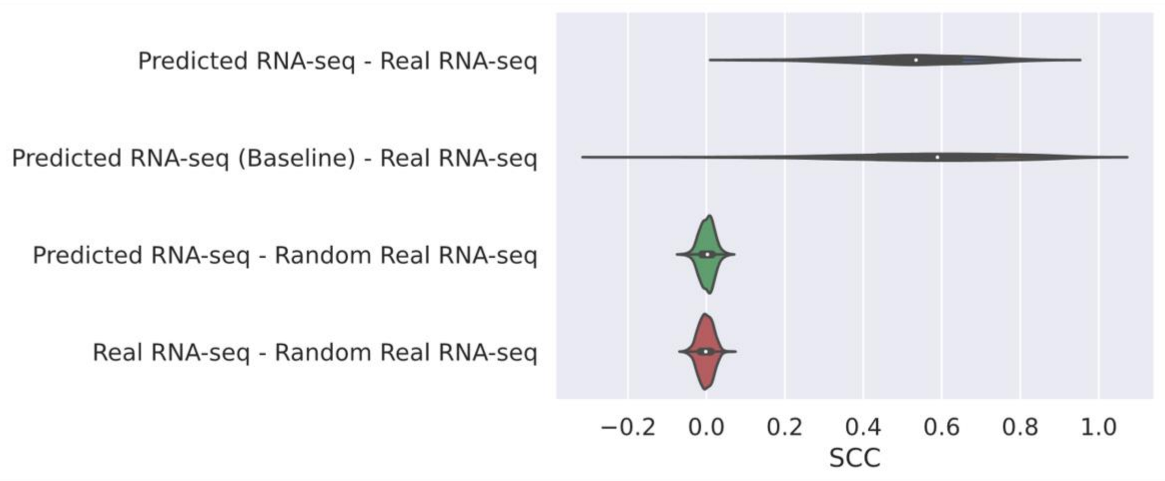

Comparing similarity in SCC between predicted and real profiles at the gene level at the 978-landmark space

Fig. S6

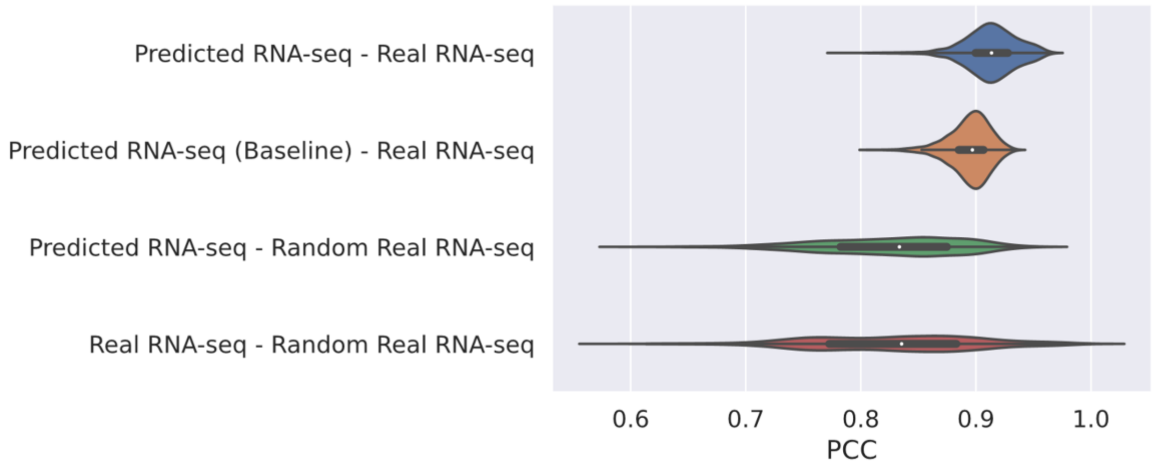

Comparing similarity in PCC between predicted and real profiles at the newly inferred space

Fig. S7

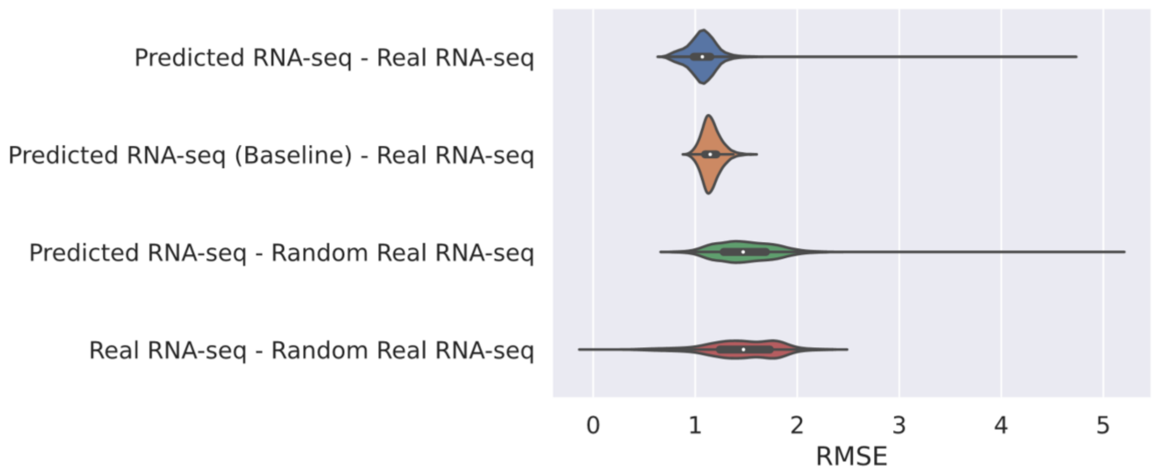

Comparing similarity in RMSE between predicted and real profiles at the newly inferred space

Fig. S8

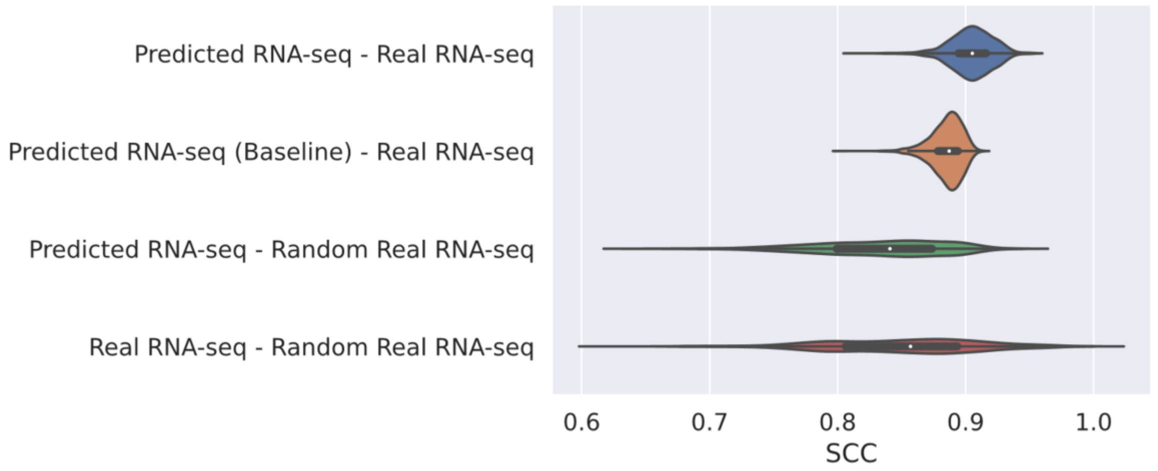

Comparing similarity in SCC between predicted and real profiles at the newly inferred space

Fig. S9

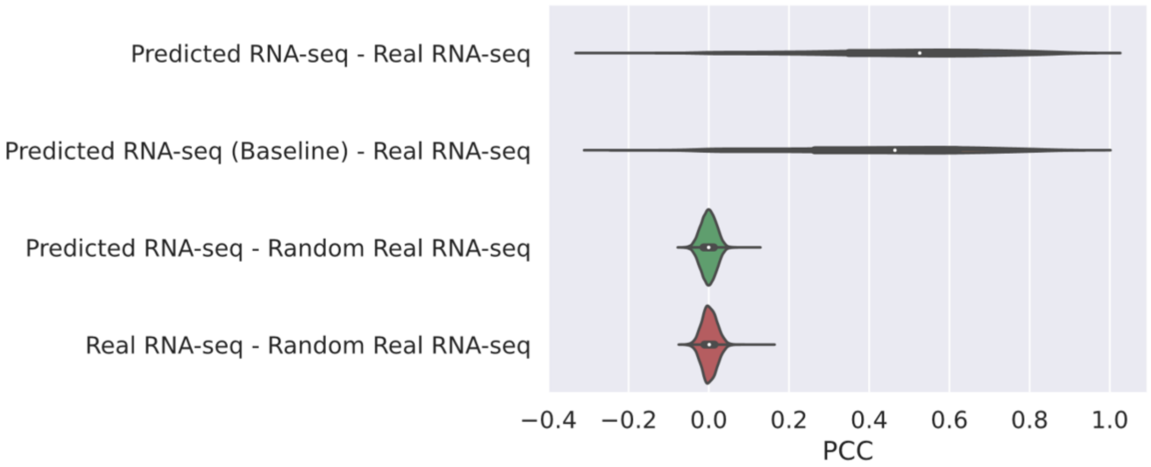

Comparing similarity in PCC between predicted and real profiles at the gene level at the newly inferred space

Fig. S10

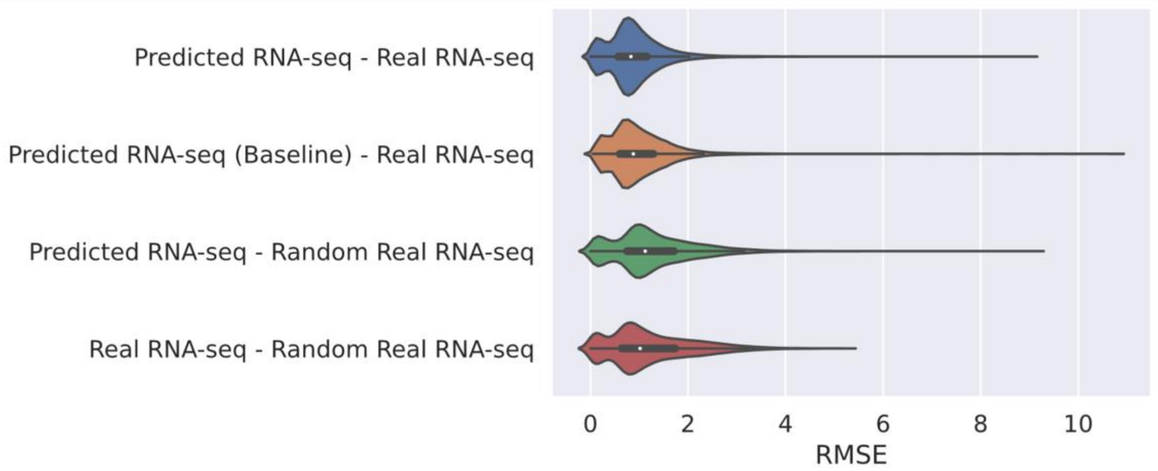

Comparing similarity in RMSE between predicted and real profiles at the gene level at the newly inferred space

Fig. S11

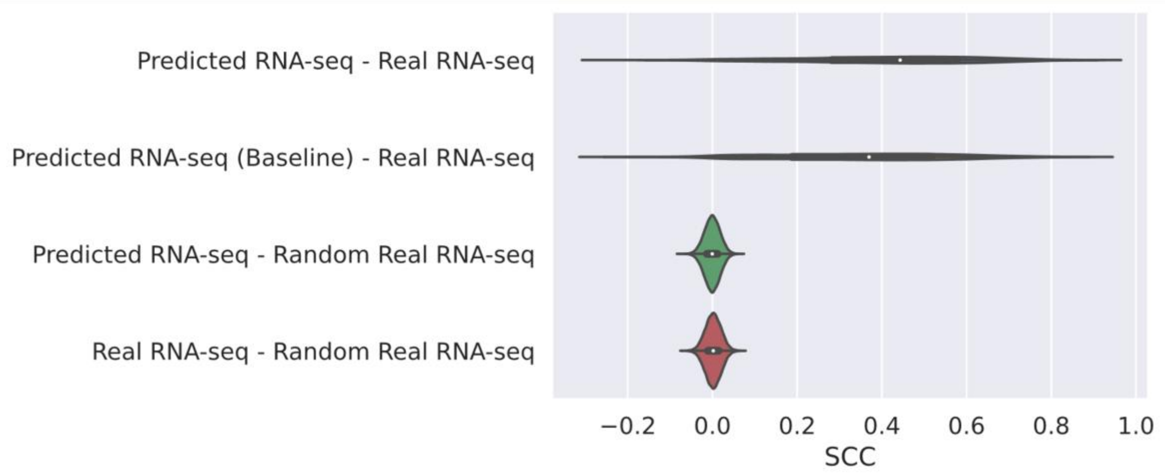

Comparing similarity in SCC between predicted and real profiles at the gene level at the newly inferred space

Fig. S12

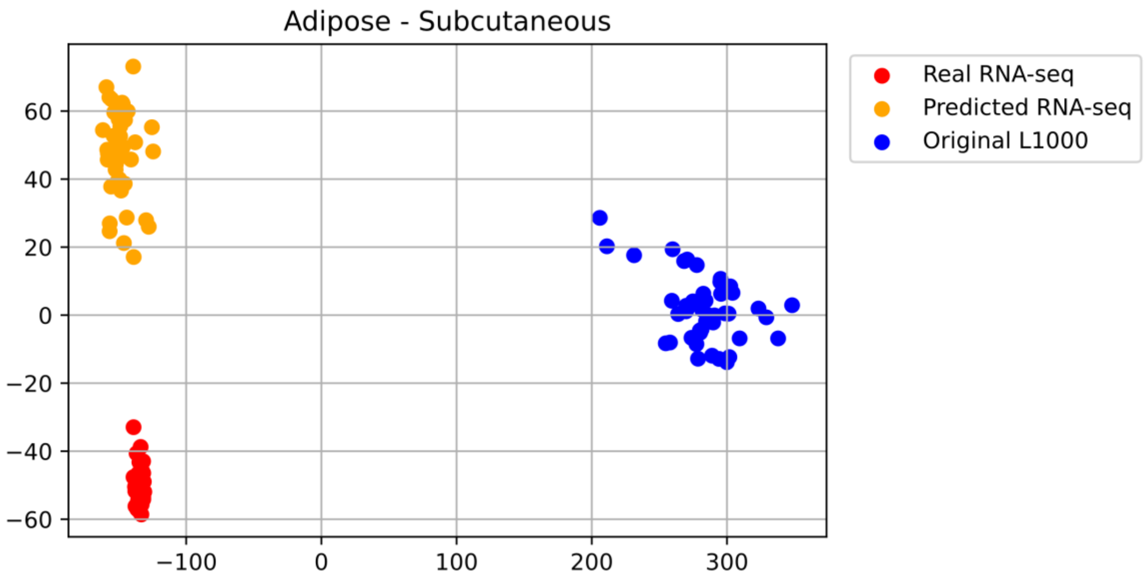
Adipose - Subcutaneous
60
40
20
0
−20
−40
−60
−100
0
100
200
300
Real RNA-seq
Predicted RNA-seq
Original L1000

Fig. S13

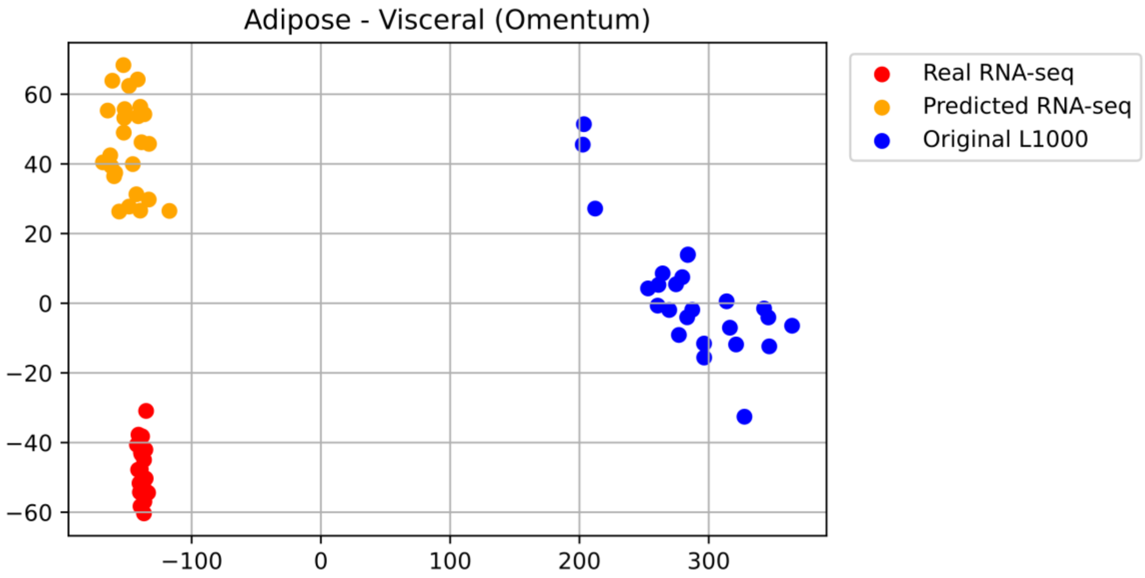
Adipose - Visceral (Omentum)
60
40
20
0
−20
−40
−60
−100
0
100
200
300
Real RNA-seq
Predicted RNA-seq
Original L1000

Fig. S14

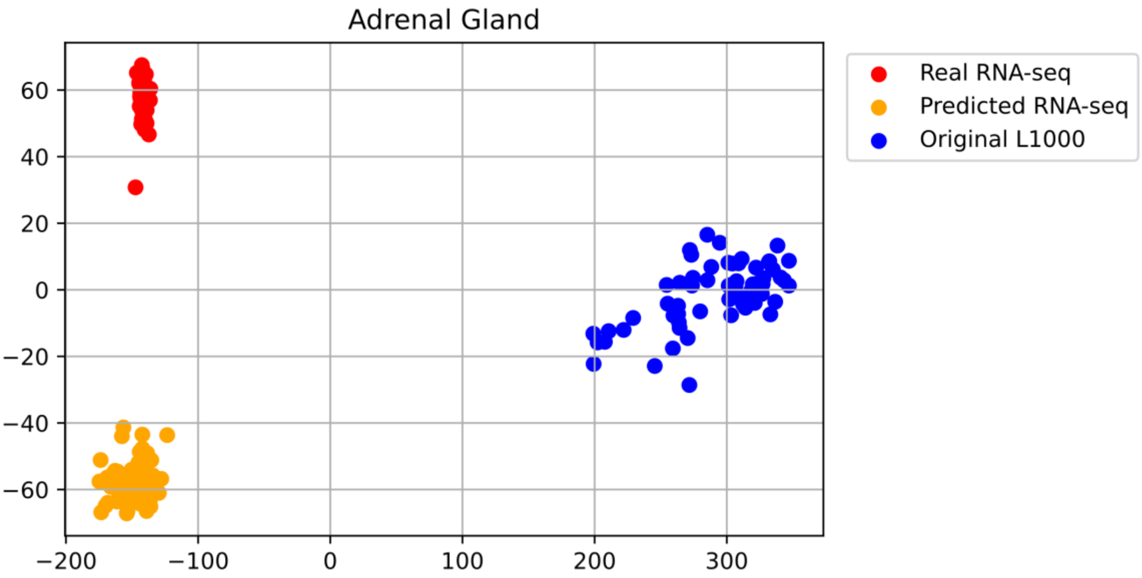
Adrenal Gland
60
40
20
0
−20
−40
−60
−200
−100
0
100
200
300
Real RNA-seq
Predicted RNA-seq
Original L1000

Fig. S15

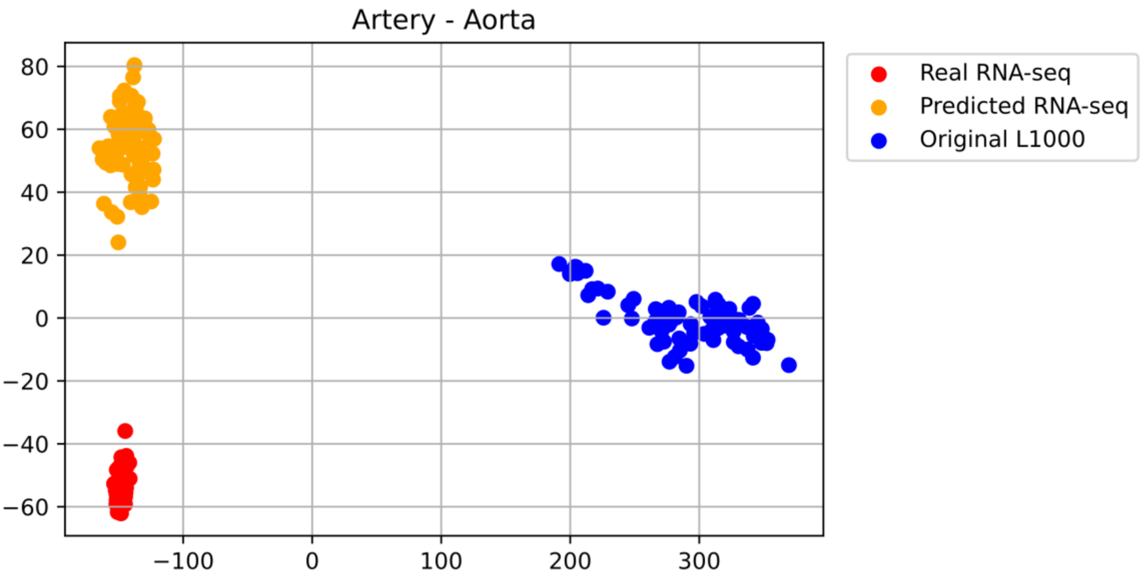
Artery - Aorta
80
60
40
20
0
−20
−40
−60
−100
0
100
200
300
Real RNA-seq
Predicted RNA-seq
Original L1000

Fig. S16

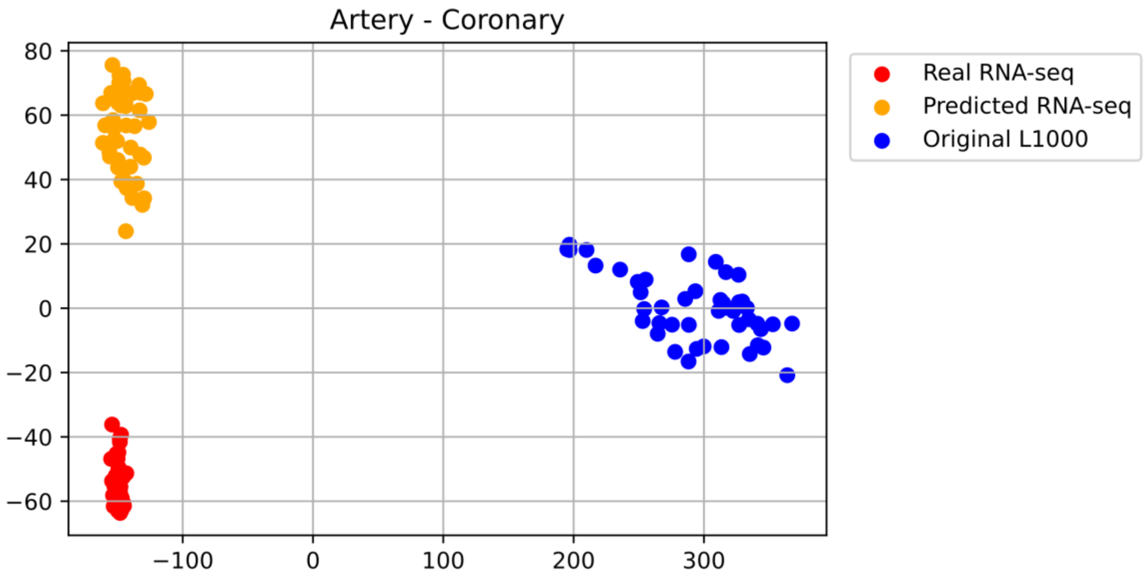
Artery - Coronary
80
60
40
20
0
−20
−40
−60
−100
0
100
200
300
Real RNA-seq
Predicted RNA-seq
Original L1000

Fig. S17

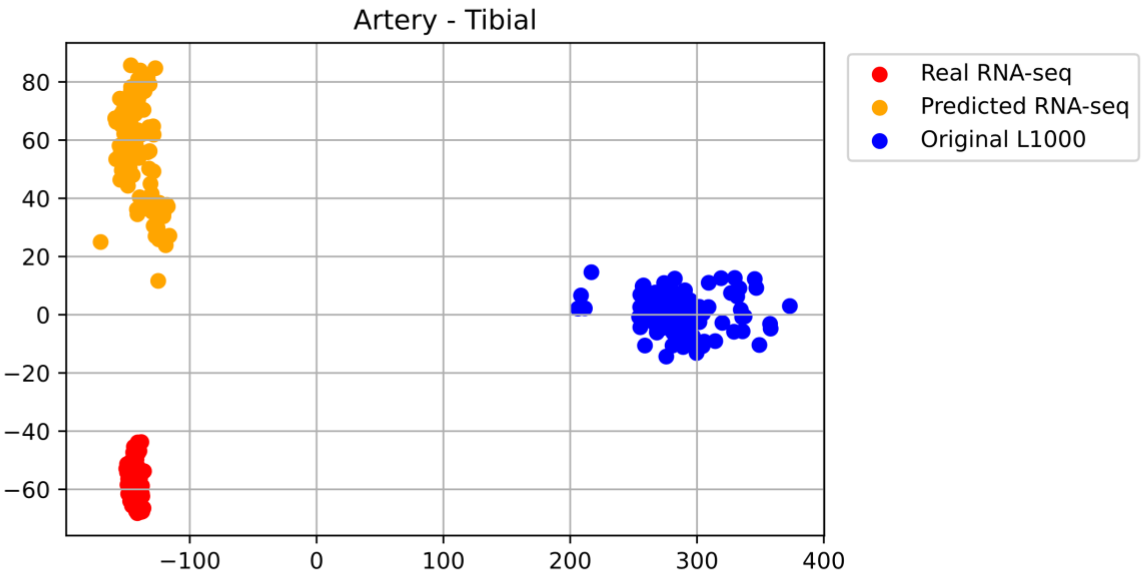
Artery - Tibial
80
60
40
20
0
−20
−40
−60
−100
0
100
200
300
400
Real RNA-seq
Predicted RNA-seq
Original L1000

Fig. S18

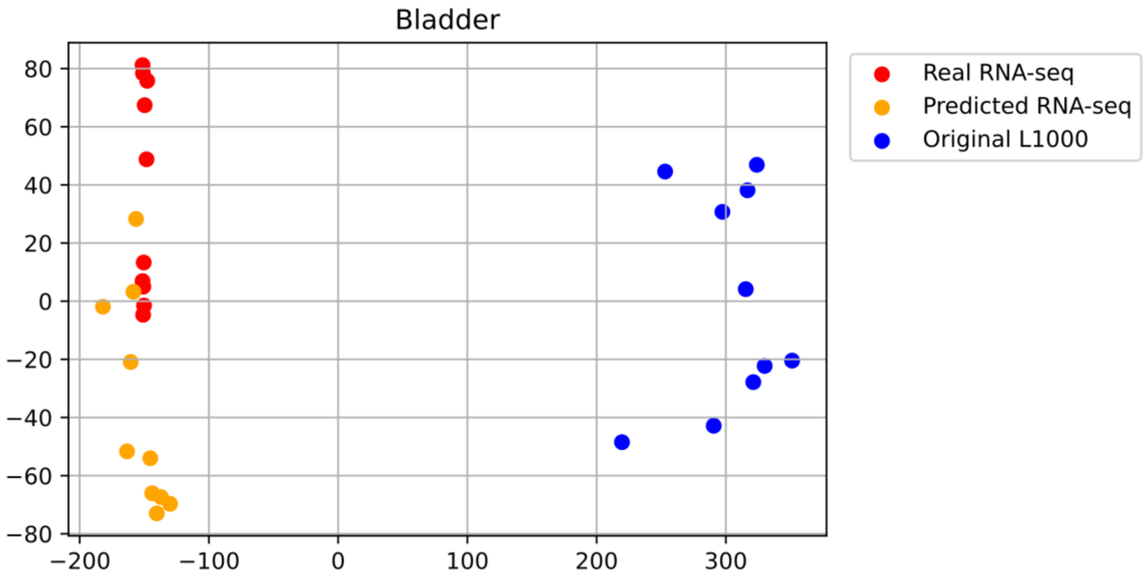
Bladder
Real RNA-seq
Predicted RNA-seq
Original L1000
80
60
40
20
0
−20
−40
−60
−80
−200
−100
0
100
200
300

Fig. S19

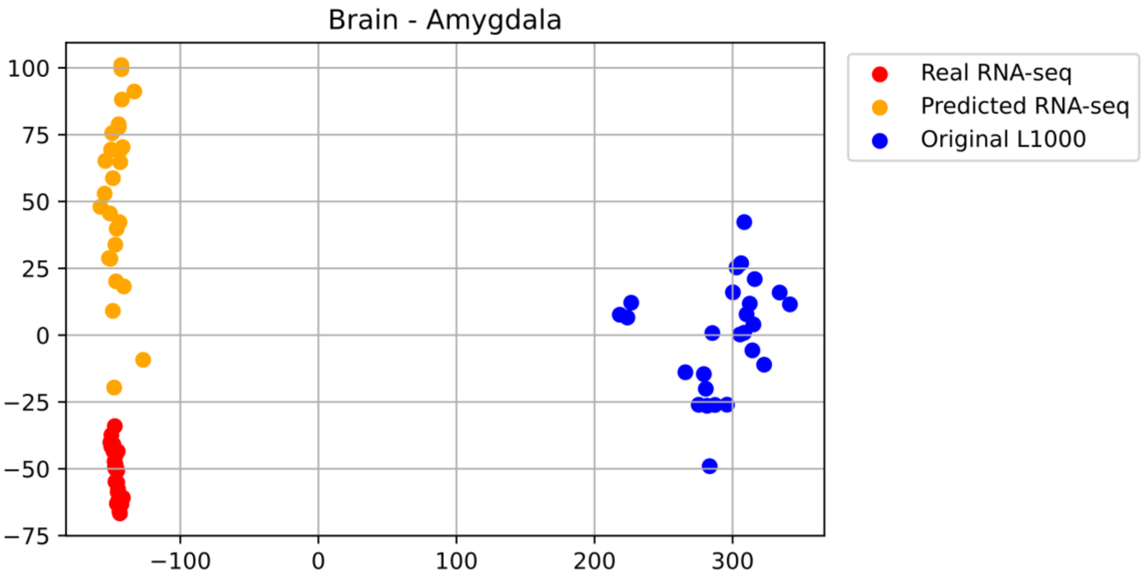
Brain - Amygdala
100
75
50
25
0
−25
−50
−75
−100
0
100
200
300
Real RNA-seq
Predicted RNA-seq
Original L1000

Fig. S20

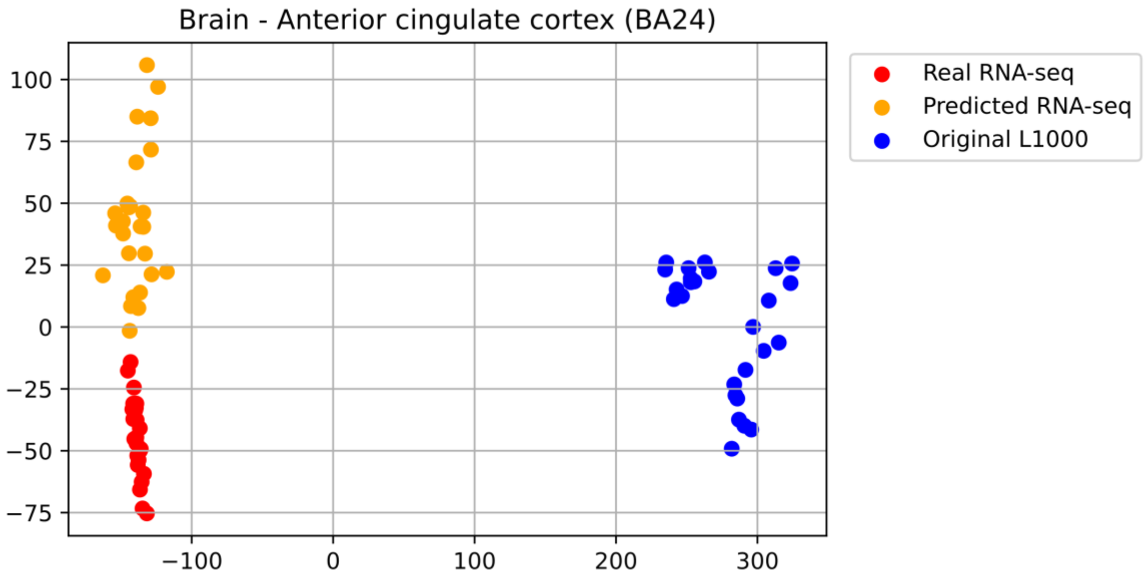
Brain - Anterior cingulate cortex (BA24)
100
75
50
25
0
−25
−50
−75
−100
0
100
200
300
Real RNA-seq
Predicted RNA-seq
Original L1000

Fig. S21

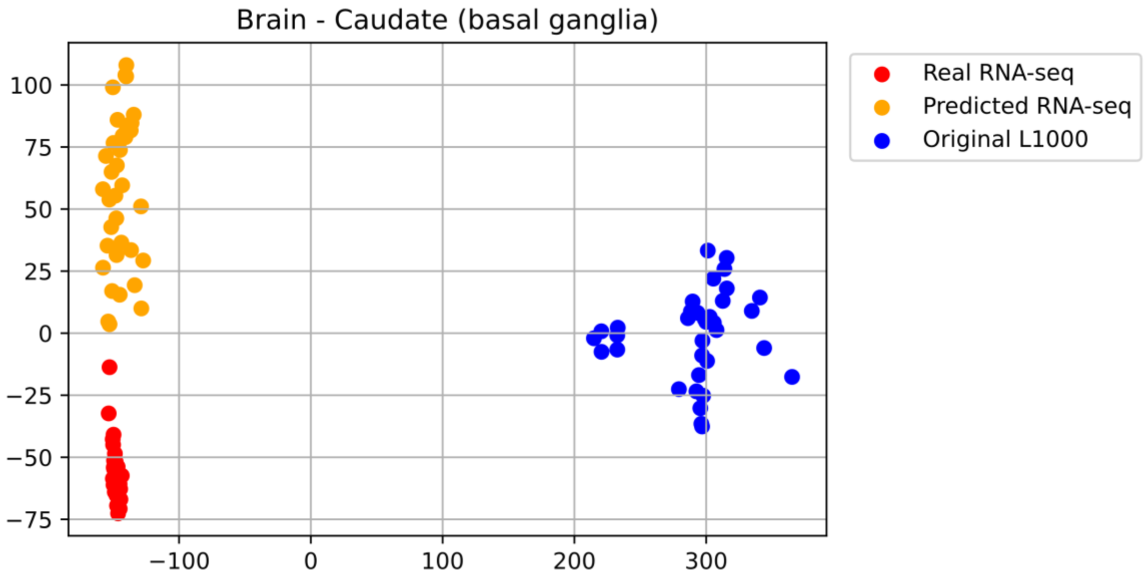
Brain - Caudate (basal ganglia)
100
75
50
25
0
−25
−50
−75
−100
0
100
200
300
Real RNA-seq
Predicted RNA-seq
Original L1000

Fig. S22

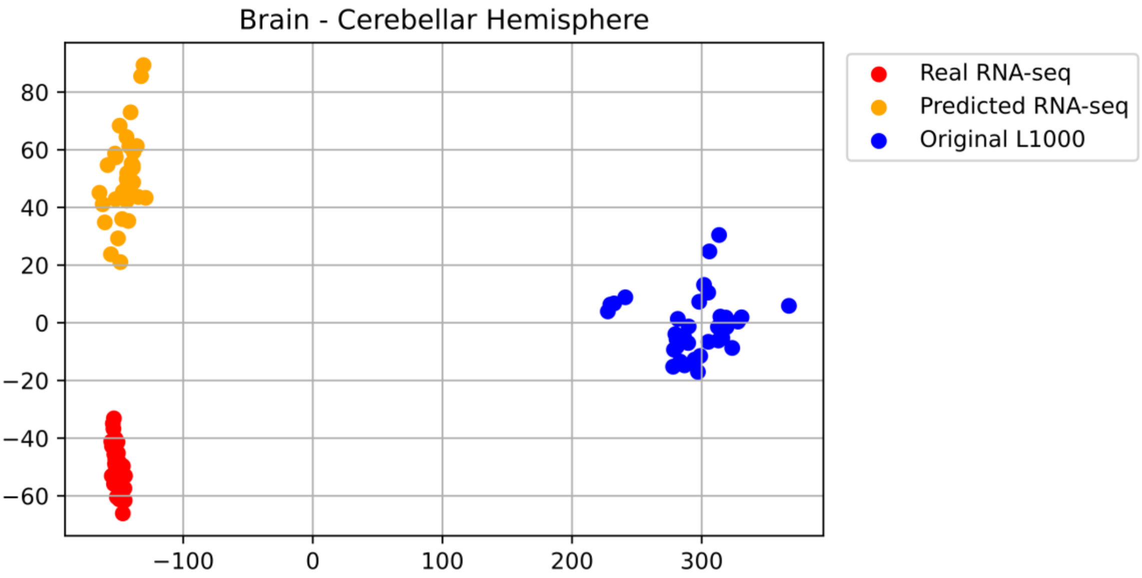

Brain - Cerebellar Hemisphere
80
60
40
20
0
−20
−40
−60
−100
0
100
200
300
Real RNA-seq
Predicted RNA-seq
Original L1000

Fig. S23

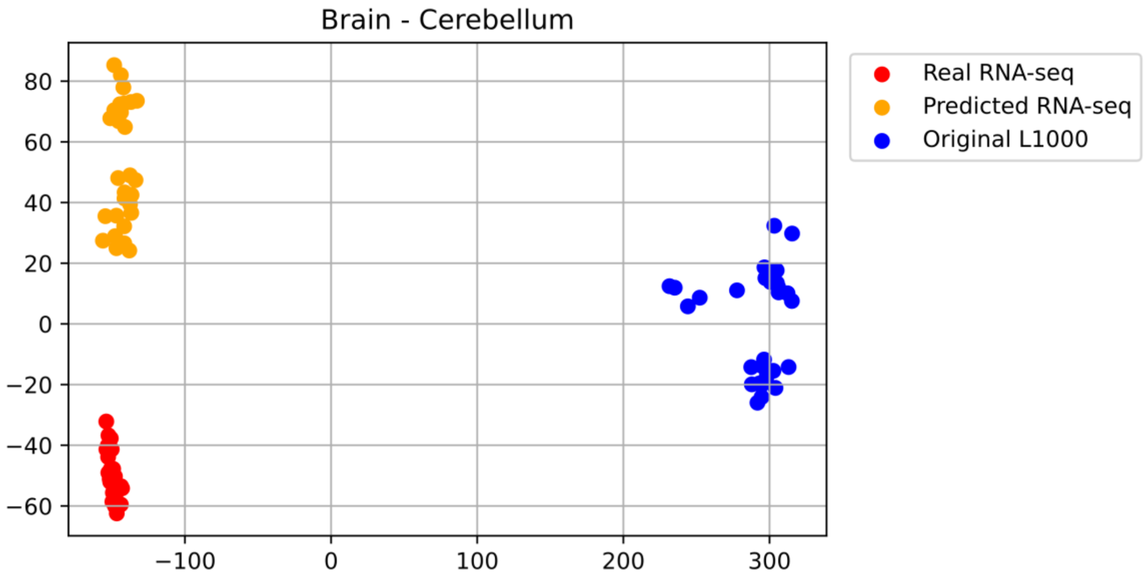
Brain - Cerebellum
Real RNA-seq
Predicted RNA-seq
Original L1000
80
60
40
20
0
−20
−40
−60
−100
0
100
200
300

Fig. S24

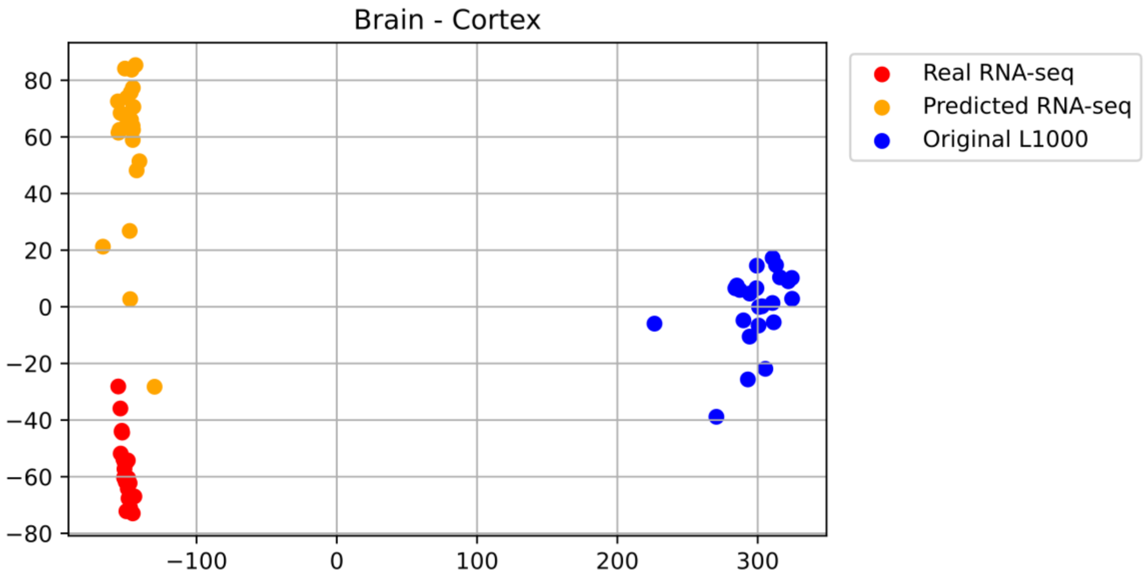
Brain - Cortex
80
60
40
20
0
−20
−40
−60
−80
−100
0
100
200
300
Real RNA-seq
Predicted RNA-seq
Original L1000

Fig. S25

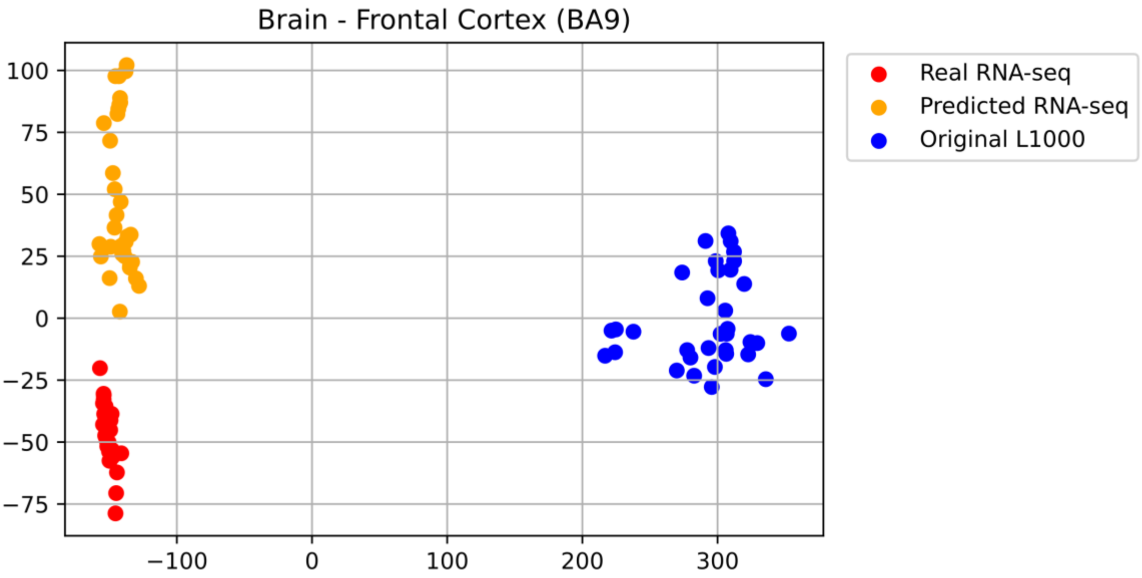
Brain - Frontal Cortex (BA9)
100
75
50
25
0
−25
−50
−75
−100
0
100
200
300
Real RNA-seq
Predicted RNA-seq
Original L1000

Fig. S26

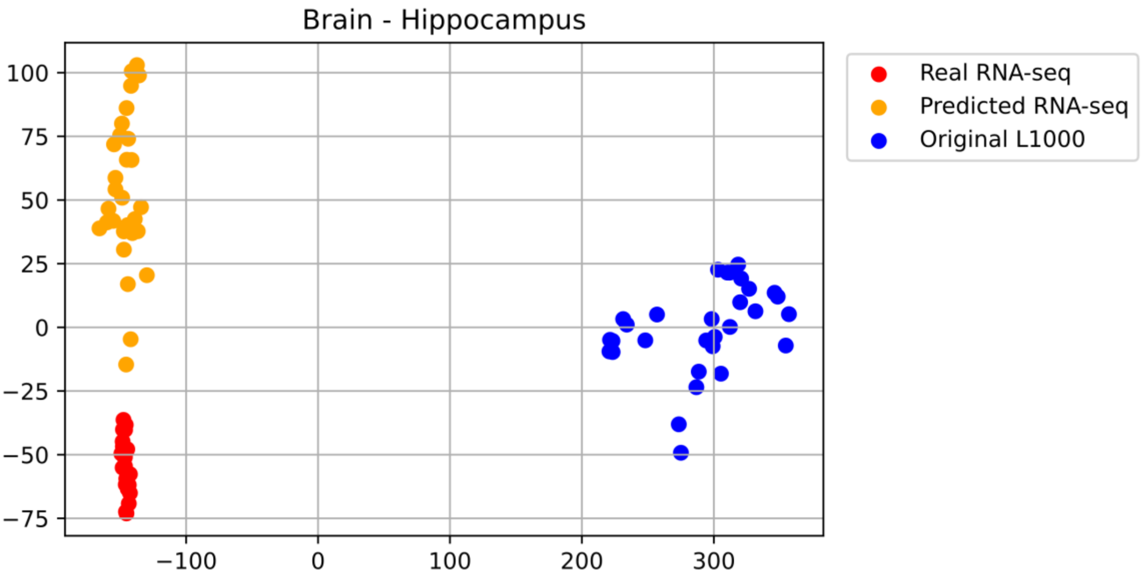

Brain - Hippocampus
100
75
50
25
0
−25
−50
−75
−100
0
100
200
300
Real RNA-seq
Predicted RNA-seq
Original L1000

Fig. S27

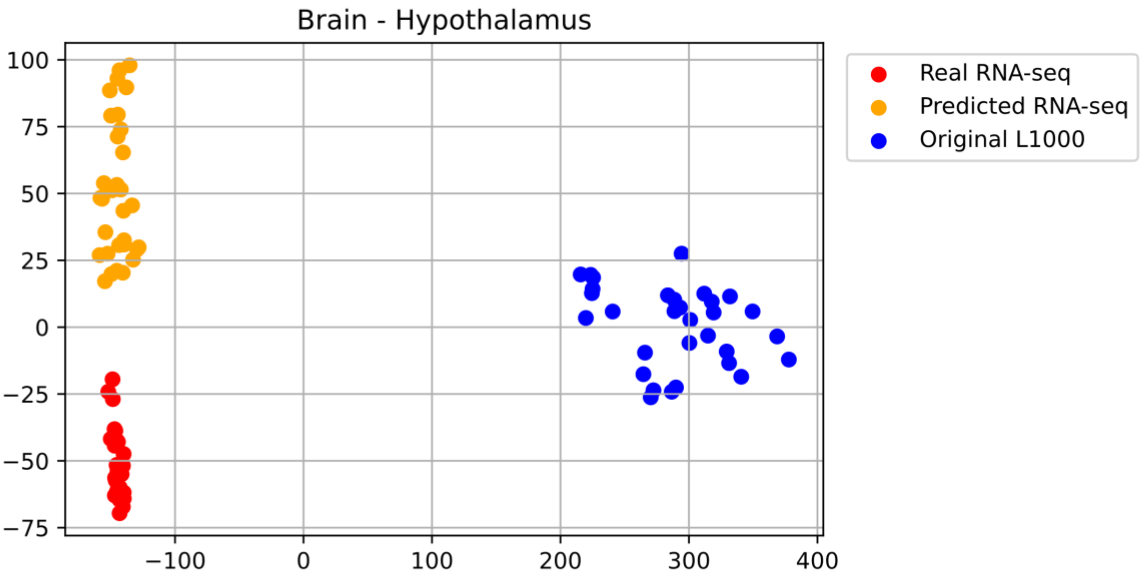
Brain - Hypothalamus
100
75
50
25
0
−25
−50
−75
−100
0
100
200
300
400
Real RNA-seq
Predicted RNA-seq
Original L1000

Fig. S28

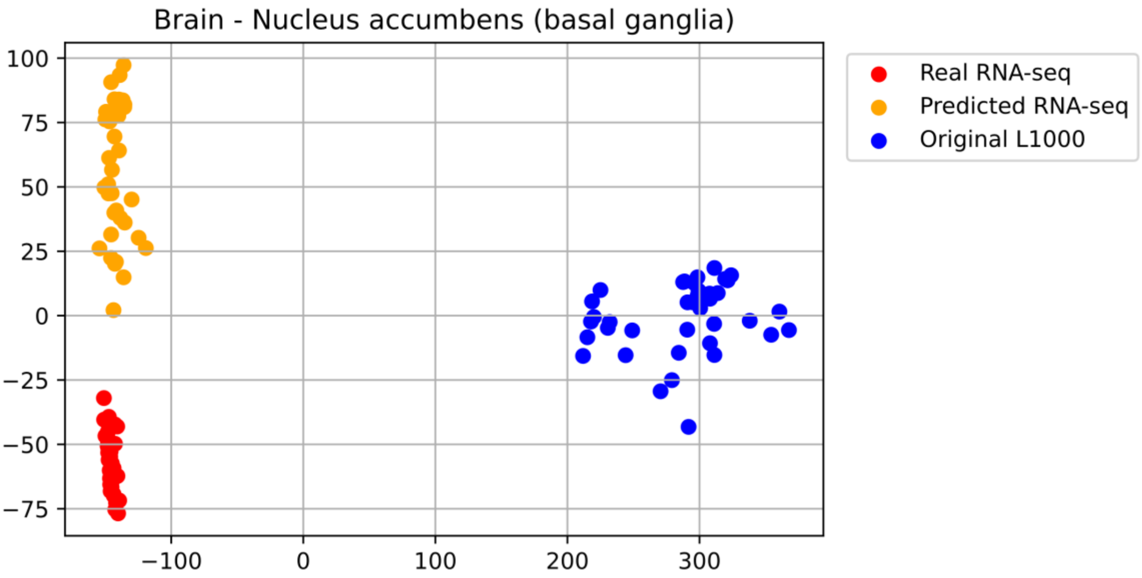
Brain - Nucleus accumbens (basal ganglia)
100
75
50
25
0
−25
−50
−75
−100
0
100
200
300
Real RNA-seq
Predicted RNA-seq
Original L1000

Fig. S29

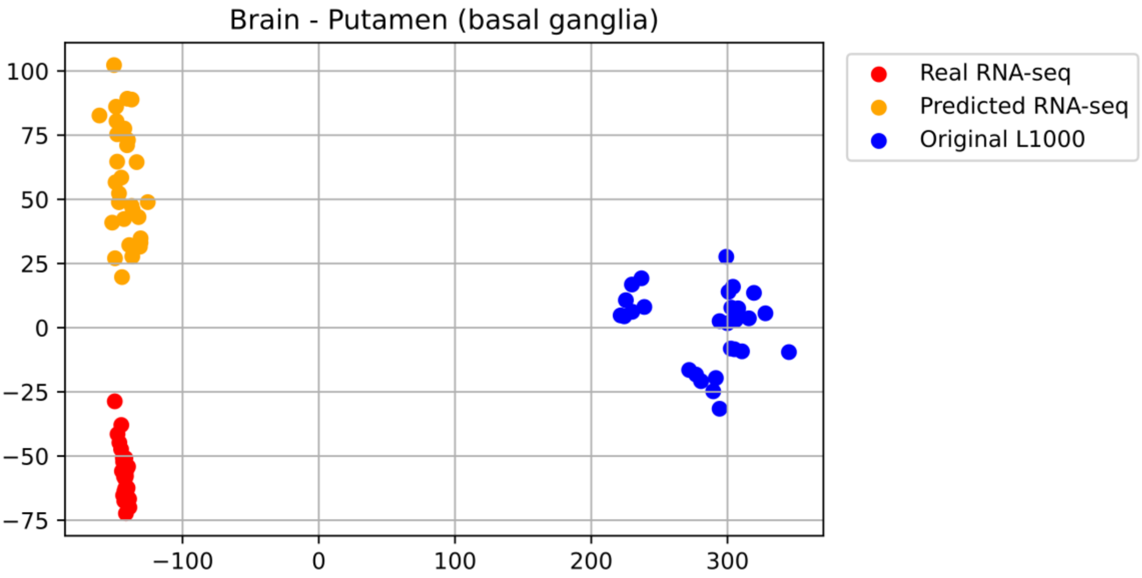
Brain - Putamen (basal ganglia)
Real RNA-seq
Predicted RNA-seq
Original L1000
100
75
50
25
0
−25
−50
−75
−100
0
100
200
300

Fig. S30

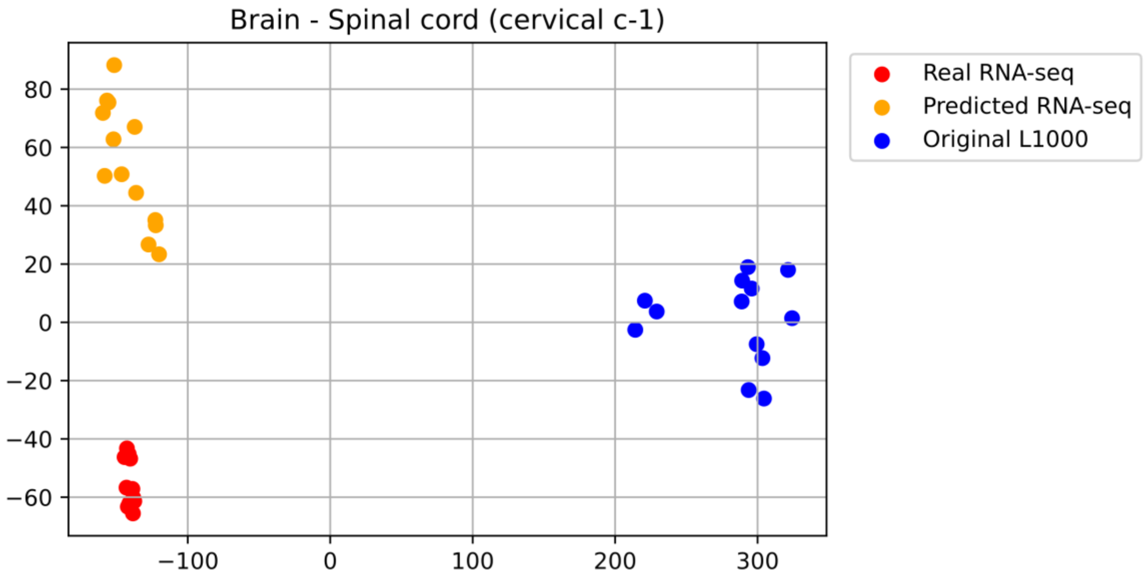
Brain - Spinal cord (cervical c-1)
80
60
40
20
0
−20
−40
−60
−100
0
100
200
300
Real RNA-seq
Predicted RNA-seq
Original L1000

Fig. S31

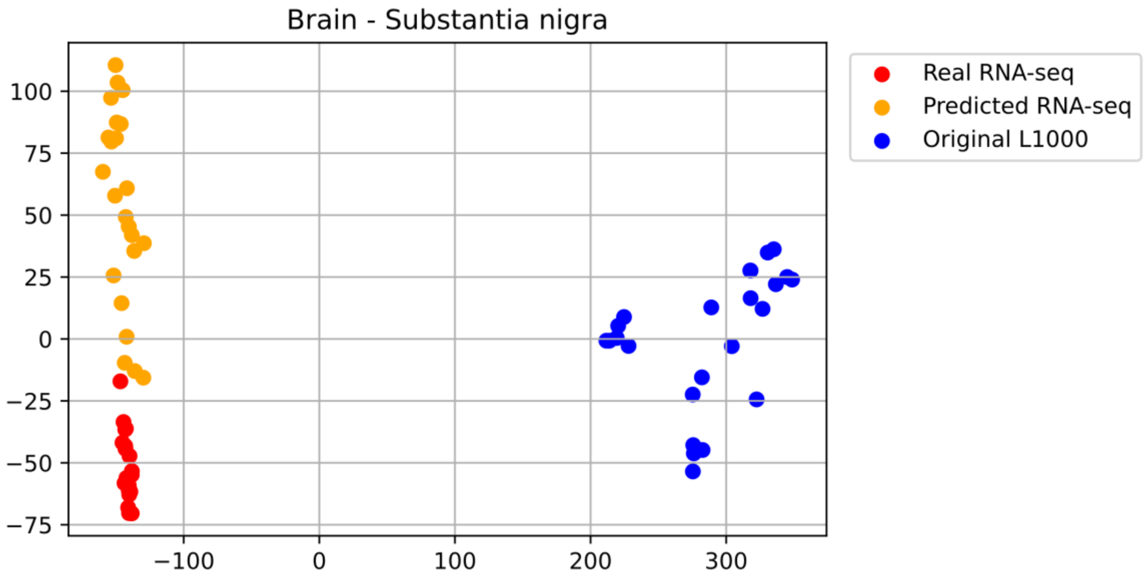
Brain - Substantia nigra
Real RNA-seq
Predicted RNA-seq
Original L1000
100
75
50
25
0
−25
−50
−75
−100
0
100
200
300

Fig. S32

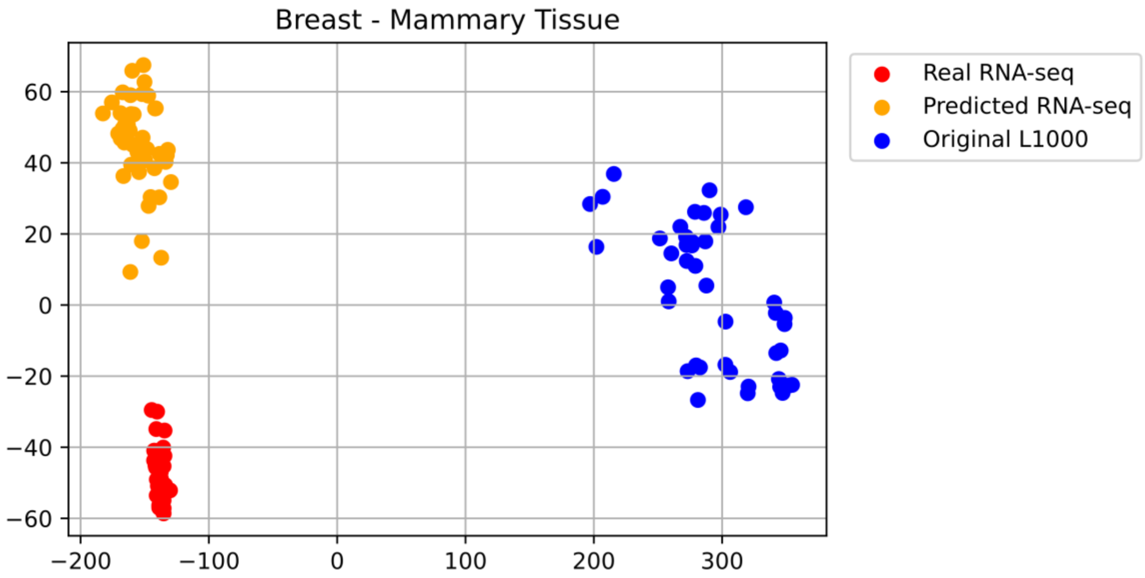
Breast - Mammary Tissue
Real RNA-seq
Predicted RNA-seq
Original L1000
60
40
20
0
−20
−40
−60
−200
−100
0
100
200
300

Fig. S33

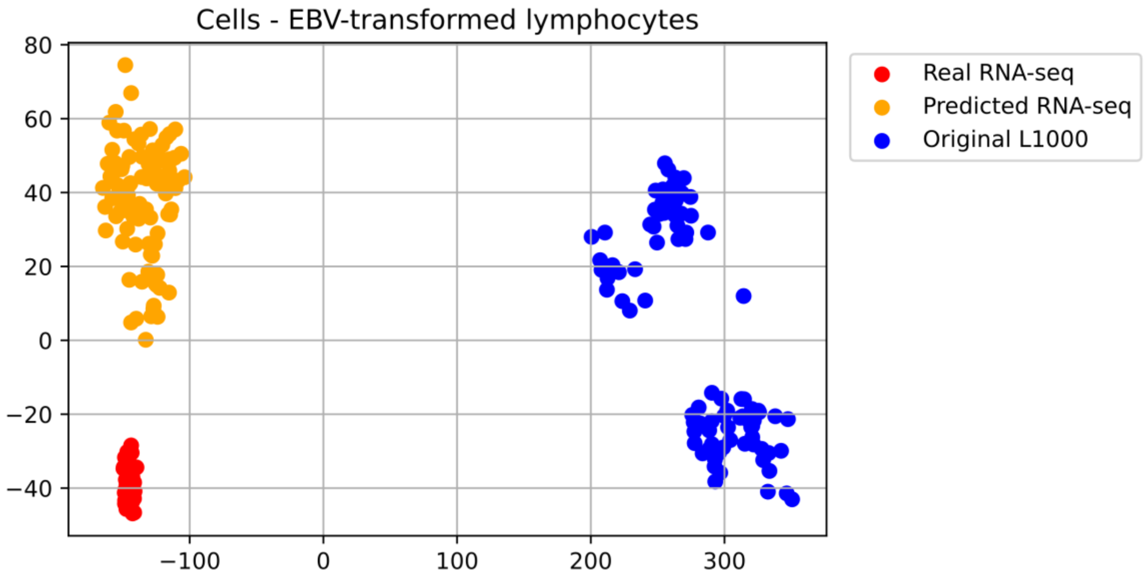
Cells - EBV-transformed lymphocytes
80
60
40
20
0
−20
−40
−100
0
100
200
300
Real RNA-seq
Predicted RNA-seq
Original L1000

Fig. S34

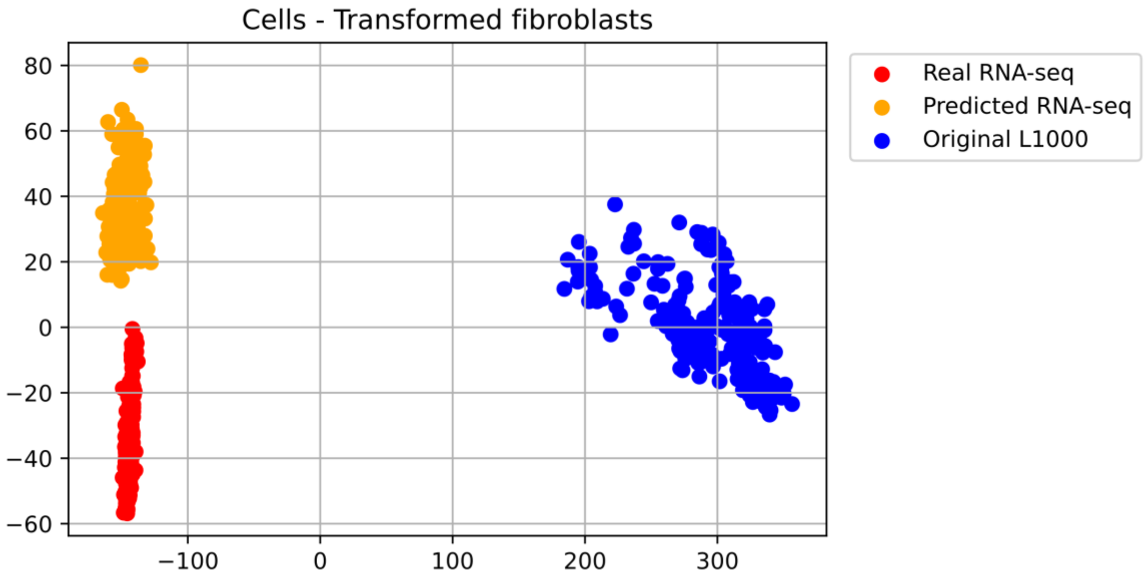
Cells - Transformed fibroblasts
80
60
40
20
0
−20
−40
−60
−100
0
100
200
300
Real RNA-seq
Predicted RNA-seq
Original L1000

Fig. S35

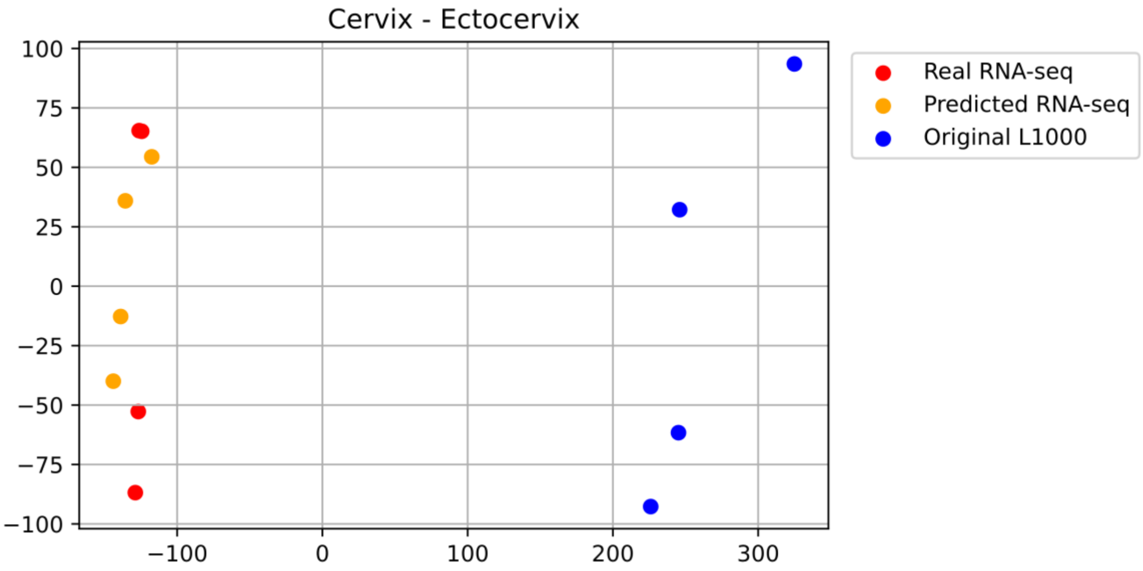
Cervix - Ectocervix
100
75
50
25
0
−25
−50
−75
−100
−100
0
100
200
300
Real RNA-seq
Predicted RNA-seq
Original L1000

Fig. S36

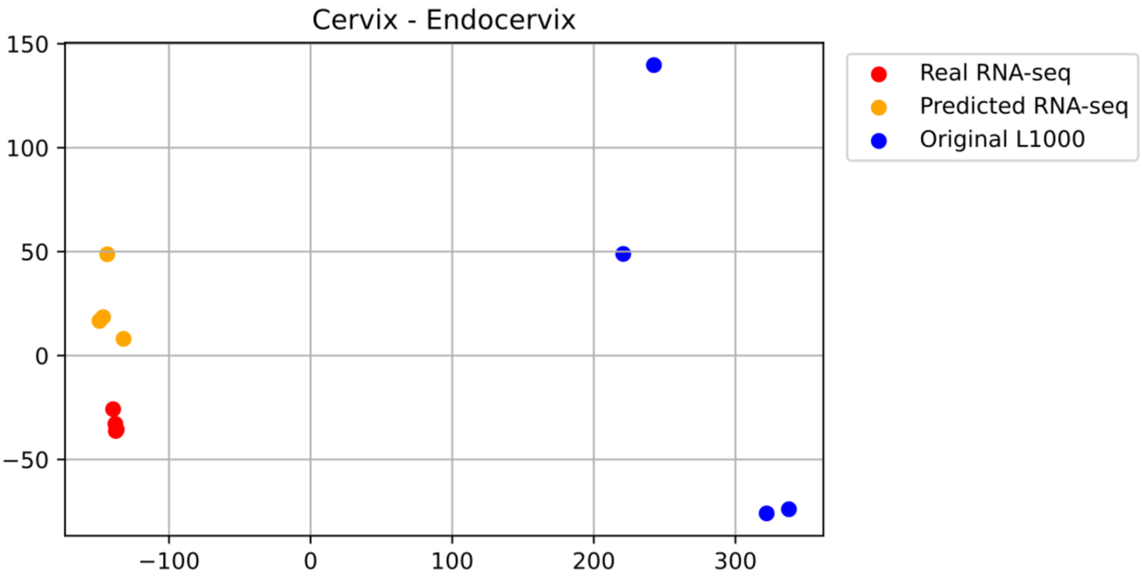

Cervix - Endocervix
150
100
50
0
−50
−100
0
100
200
300
Real RNA-seq
Predicted RNA-seq
Original L1000

Fig. S37

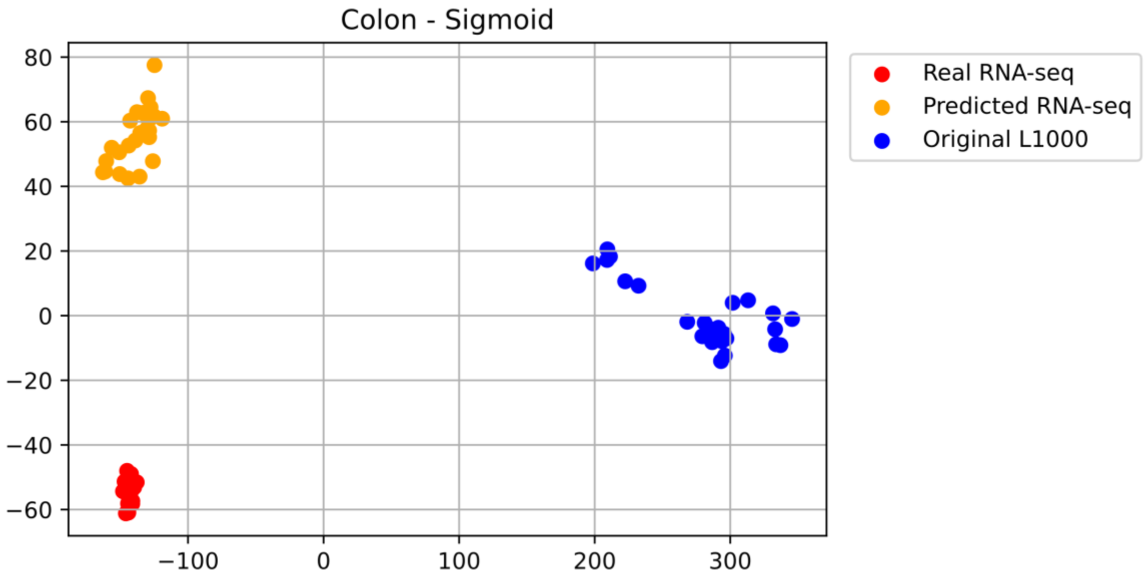
Colon - Sigmoid
80
60
40
20
0
−20
−40
−60
−100
0
100
200
300
Real RNA-seq
Predicted RNA-seq
Original L1000

Fig. S38

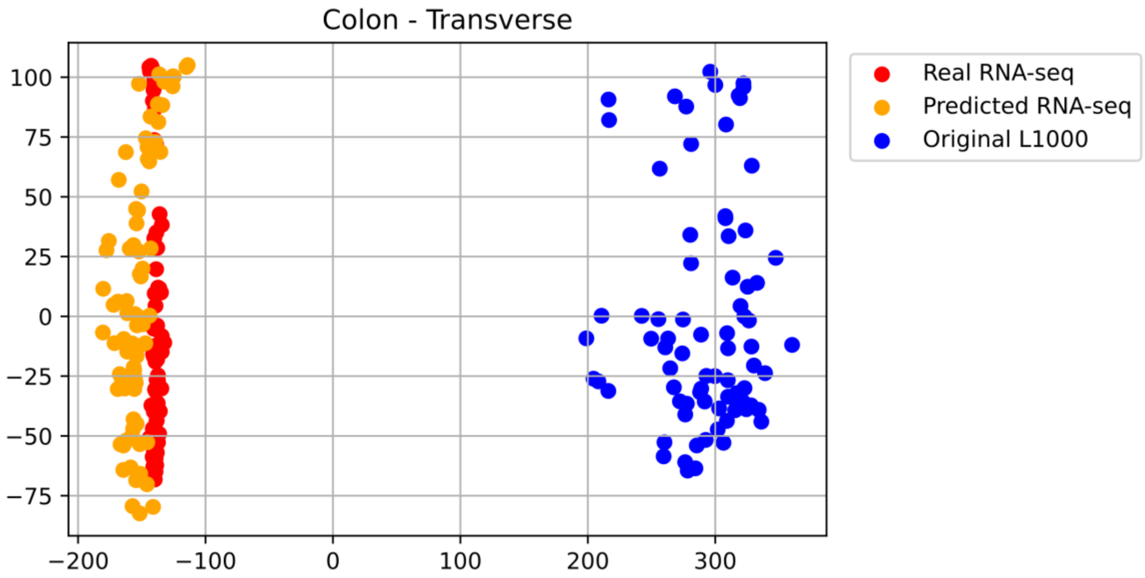
Colon - Transverse
Real RNA-seq
Predicted RNA-seq
Original L1000
100
75
50
25
0
−25
−50
−75
−200
−100
0
100
200
300

Fig. S39

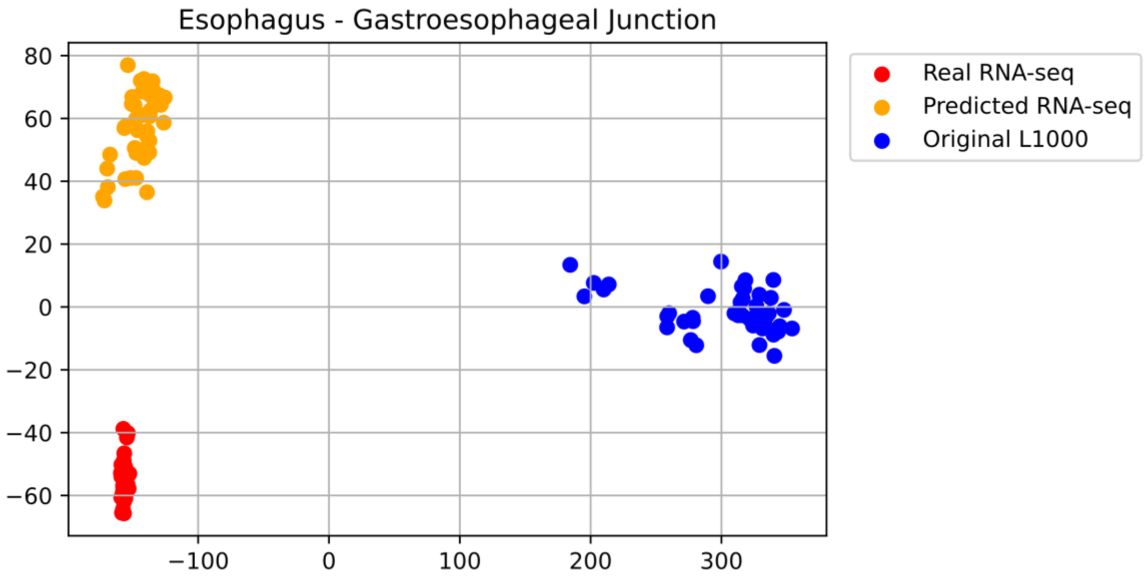

Esophagus - Gastroesophageal Junction
80
60
40
20
0
−20
−40
−60
−100
0
100
200
300
Real RNA-seq
Predicted RNA-seq
Original L1000

Fig. S40

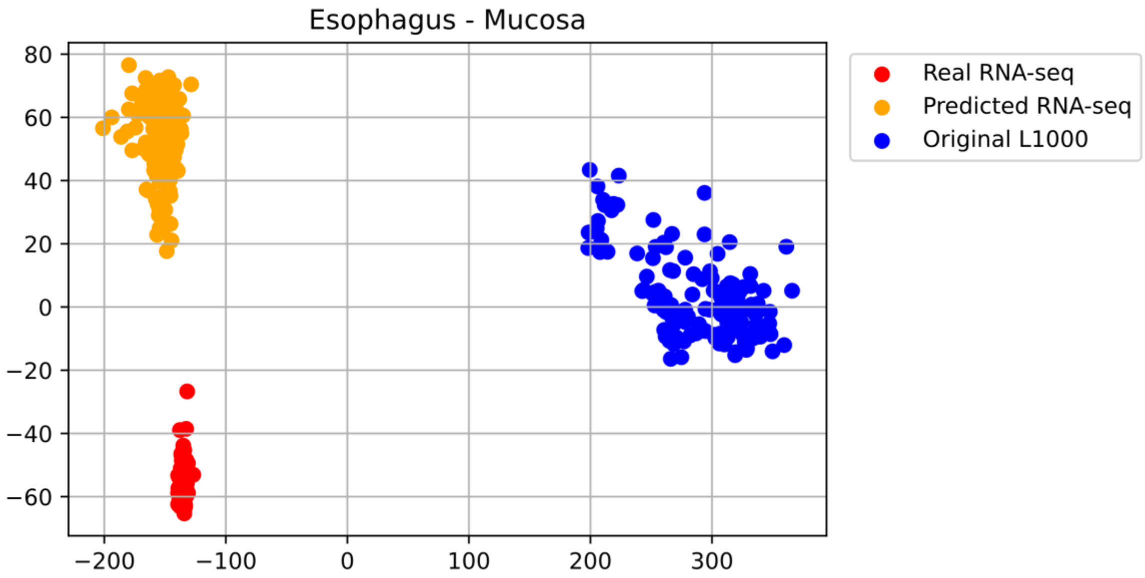
Esophagus - Mucosa
80
60
40
20
0
−20
−40
−60
−200
−100
0
100
200
300
Real RNA-seq
Predicted RNA-seq
Original L1000

Fig. S41

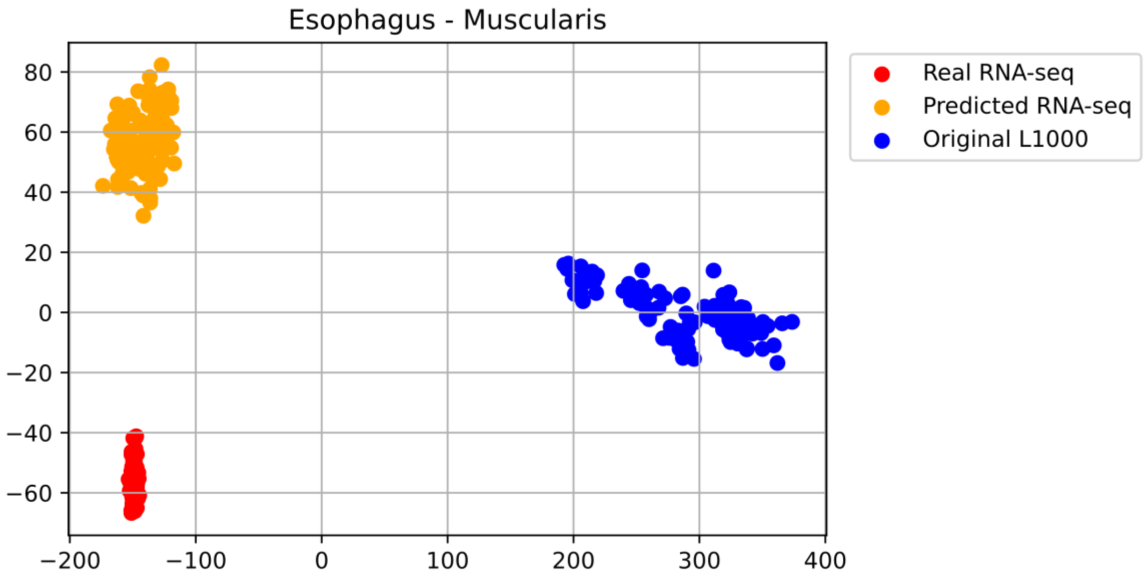

Esophagus - Muscularis
80
60
40
20
0
−20
−40
−60
−200
−100
0
100
200
300
400
Real RNA-seq
Predicted RNA-seq
Original L1000

Fig. S42

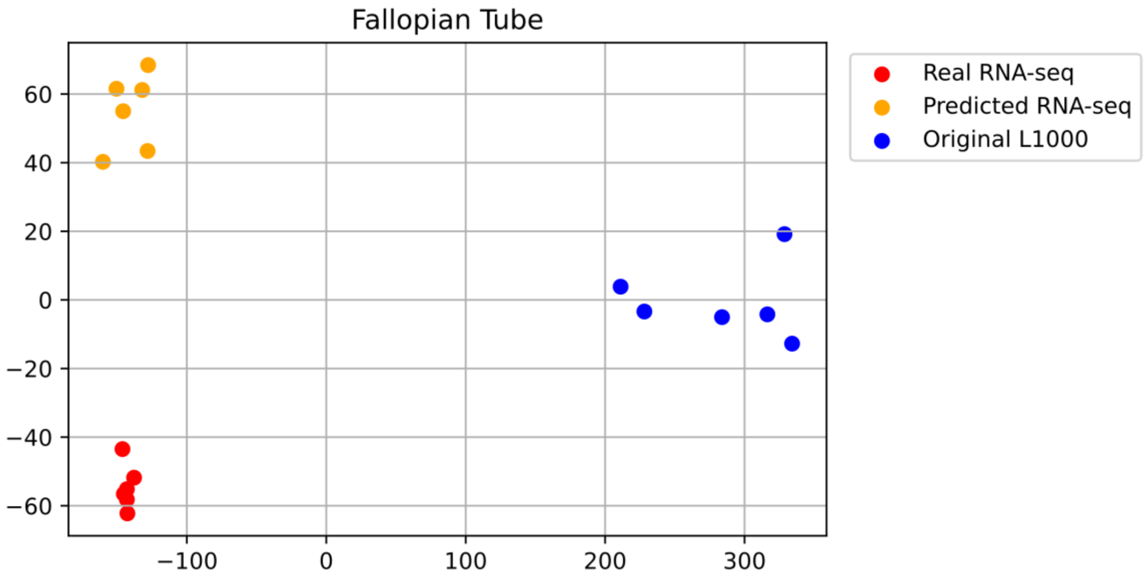
Fallopian Tube
60
40
20
0
−20
−40
−60
−100
0
100
200
300
Real RNA-seq
Predicted RNA-seq
Original L1000

Fig. S43

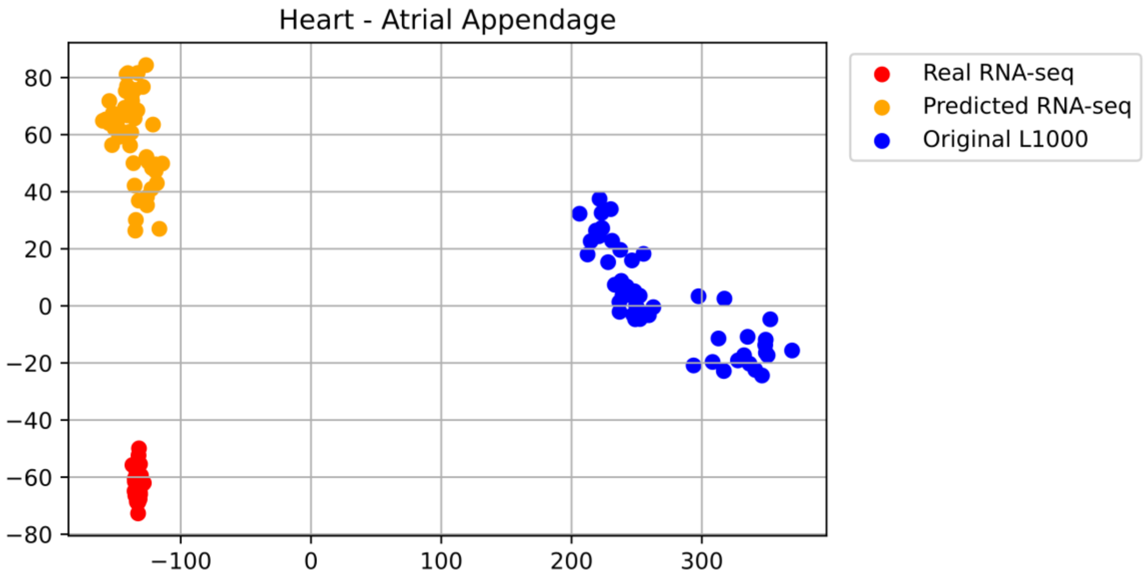

Heart - Atrial Appendage
80
60
40
20
0
−20
−40
−60
−80
−100
0
100
200
300
Real RNA-seq
Predicted RNA-seq
Original L1000

Fig. S44

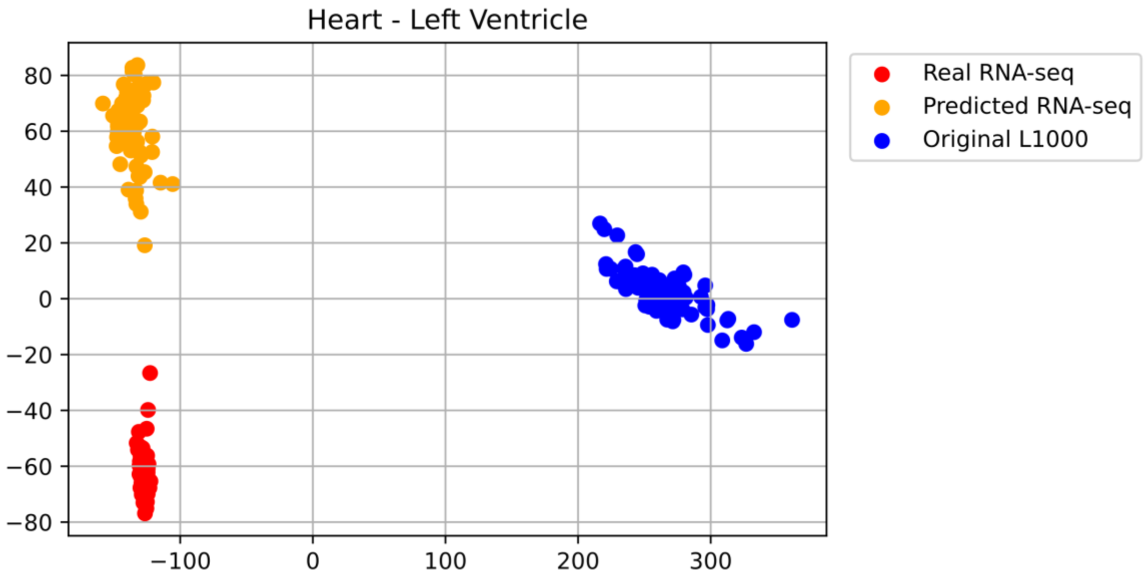
Heart - Left Ventricle
Real RNA-seq
Predicted RNA-seq
Original L1000
80
60
40
20
0
−20
−40
−60
−80
−100
0
100
200
300

Fig. S45

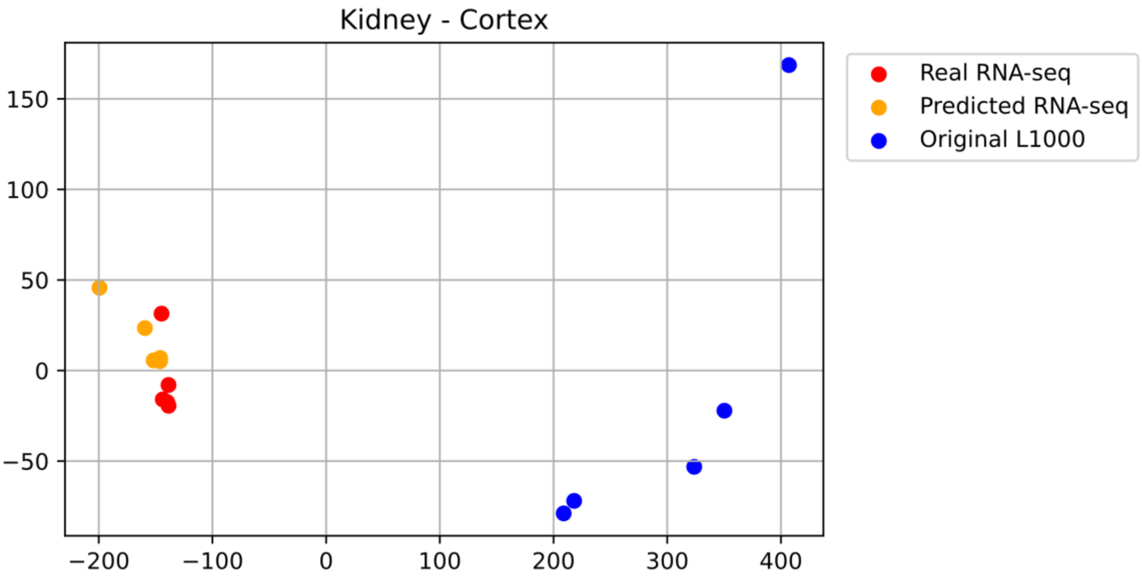
Kidney - Cortex
150
100
50
0
−50
−200
−100
0
100
200
300
400
Real RNA-seq
Predicted RNA-seq
Original L1000

Fig. S46

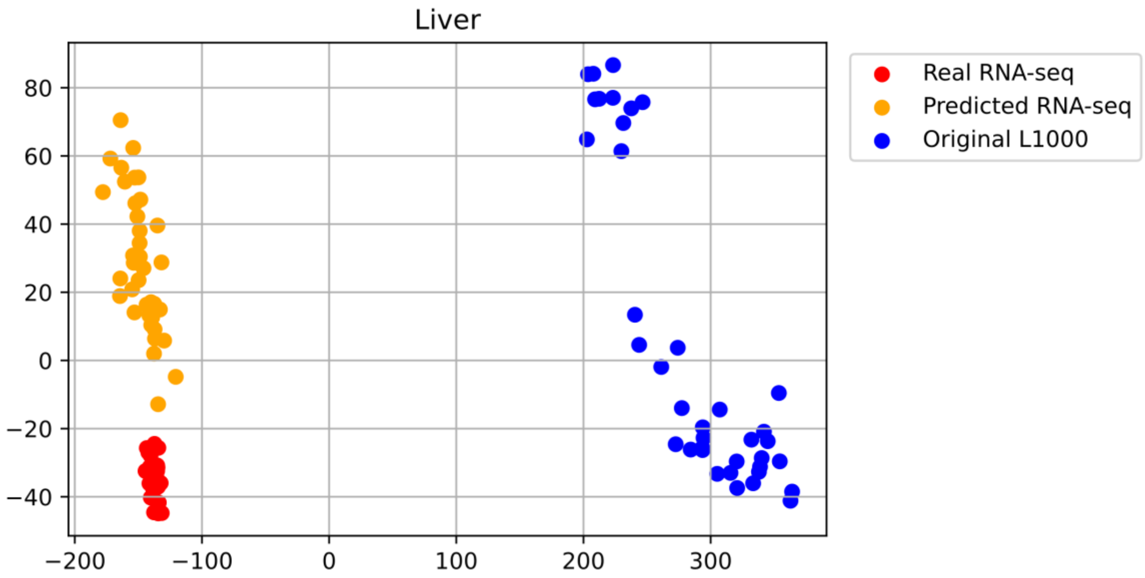
Liver
80
60
40
20
0
−20
−40
−200
−100
0
100
200
300
Real RNA-seq
Predicted RNA-seq
Original L1000

Fig. S47

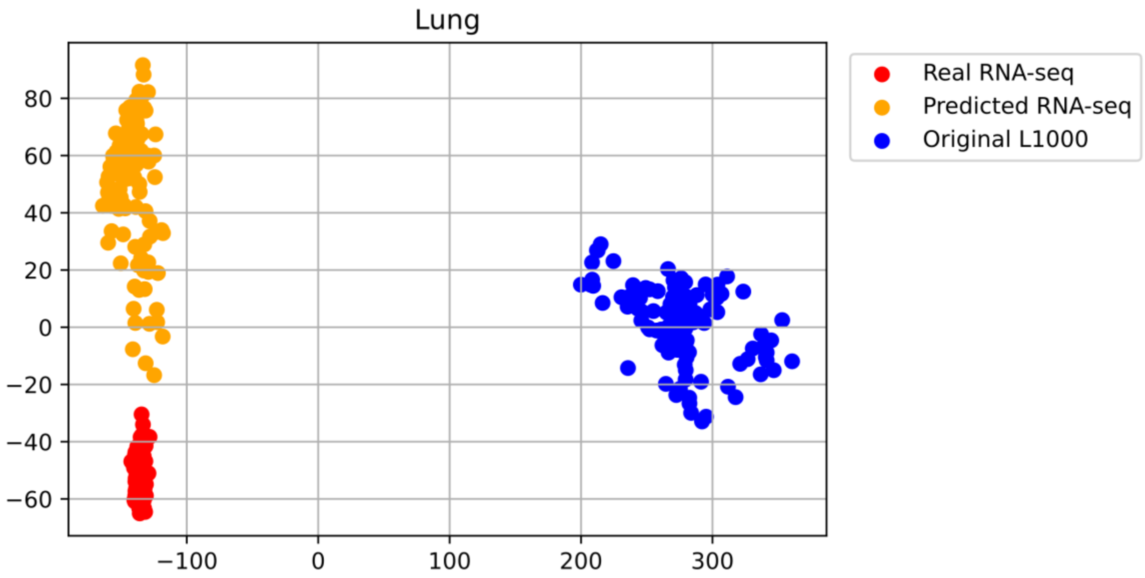
Lung
80
60
40
20
0
−20
−40
−60
−100
0
100
200
300
Real RNA-seq
Predicted RNA-seq
Original L1000

Fig. S48

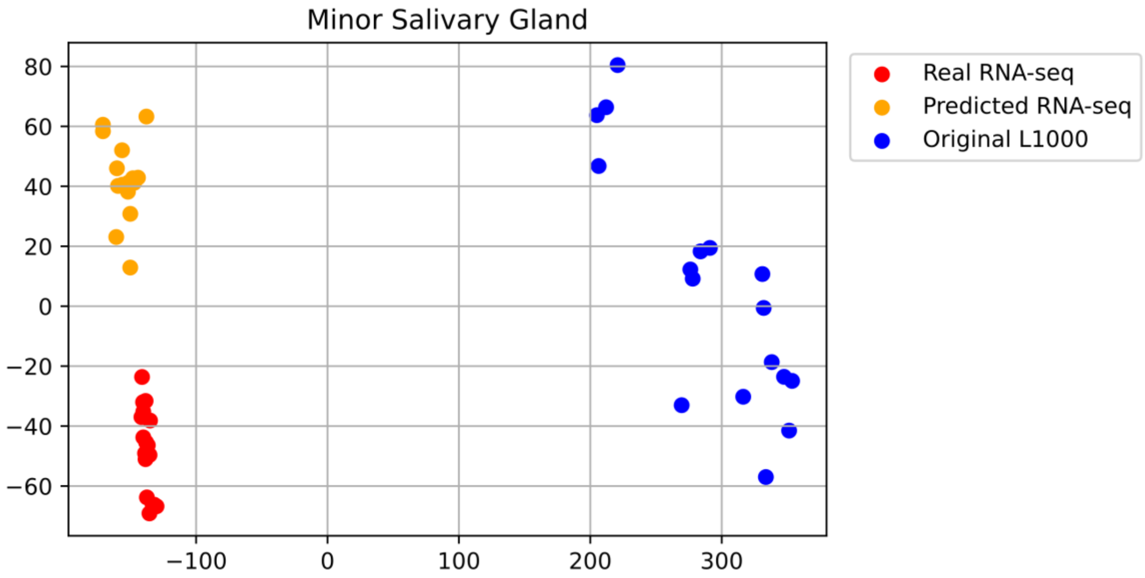

Minor Salivary Gland
80
60
40
20
0
−20
−40
−60
−100
0
100
200
300
Real RNA-seq
Predicted RNA-seq
Original L1000

Fig. S49

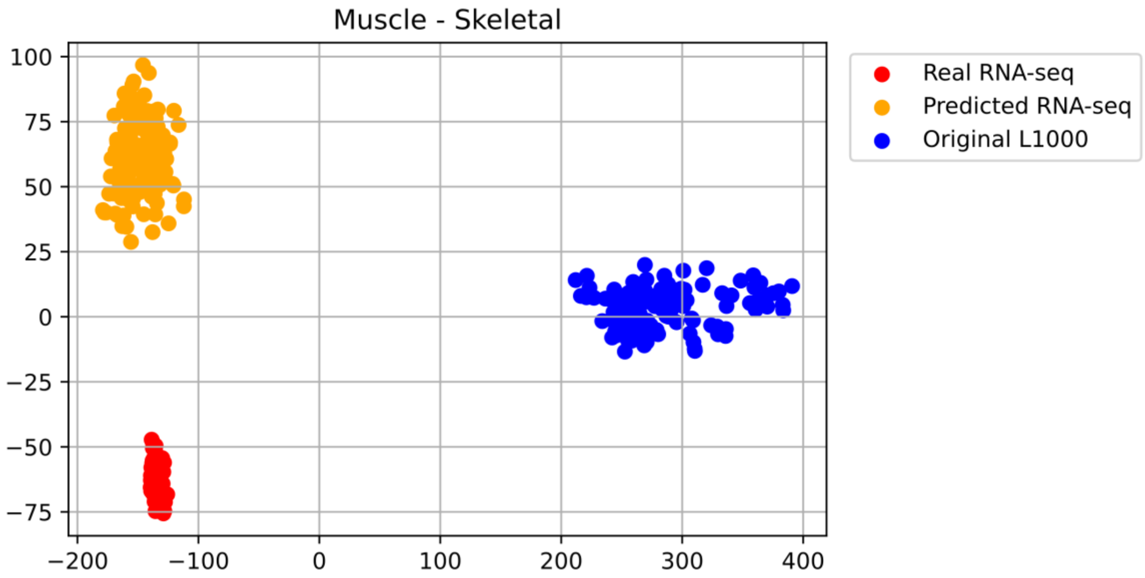
Muscle - Skeletal
100
75
50
25
0
−25
−50
−75
−200
−100
0
100
200
300
400
Real RNA-seq
Predicted RNA-seq
Original L1000

Fig. S50

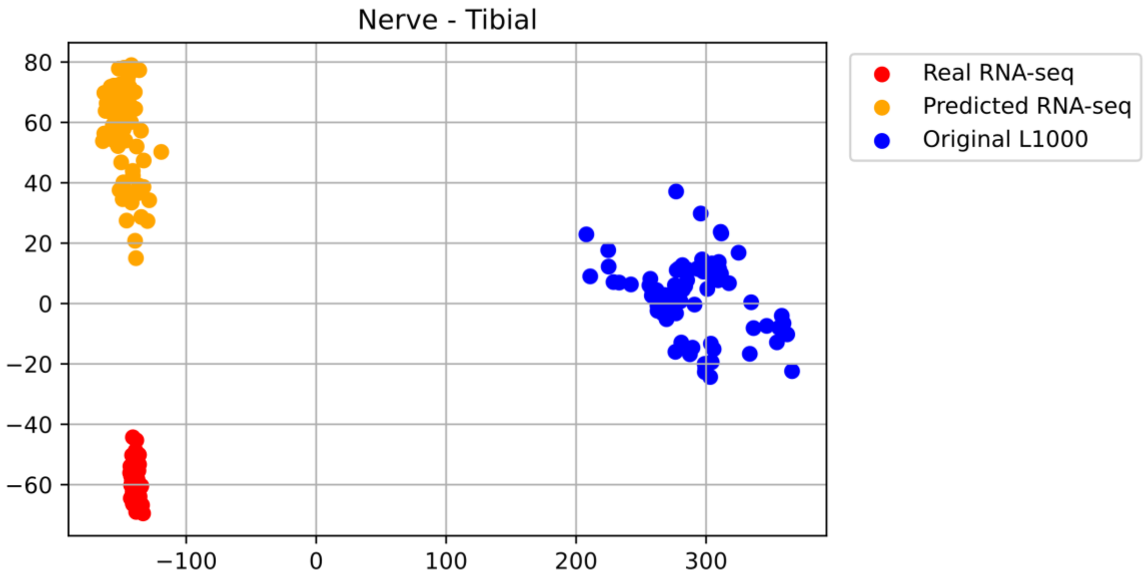
Nerve - Tibial
Real RNA-seq
Predicted RNA-seq
Original L1000
80
60
40
20
0
−20
−40
−60
−100
0
100
200
300

Fig. S51

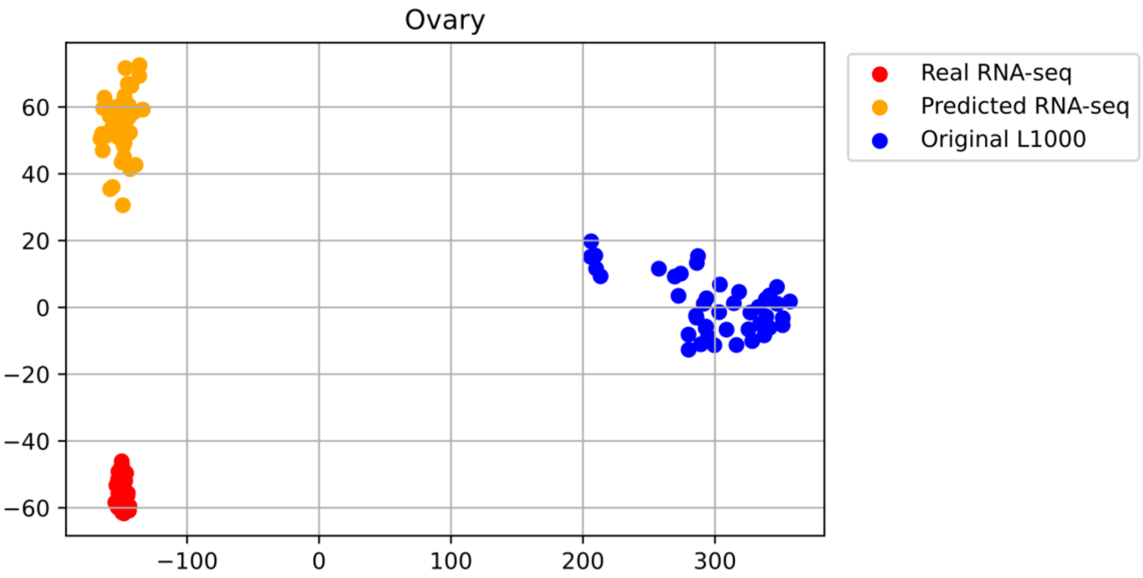

Ovary
60
40
20
0
−20
−40
−60
−100
0
100
200
300
Real RNA-seq
Predicted RNA-seq
Original L1000

Fig. S52

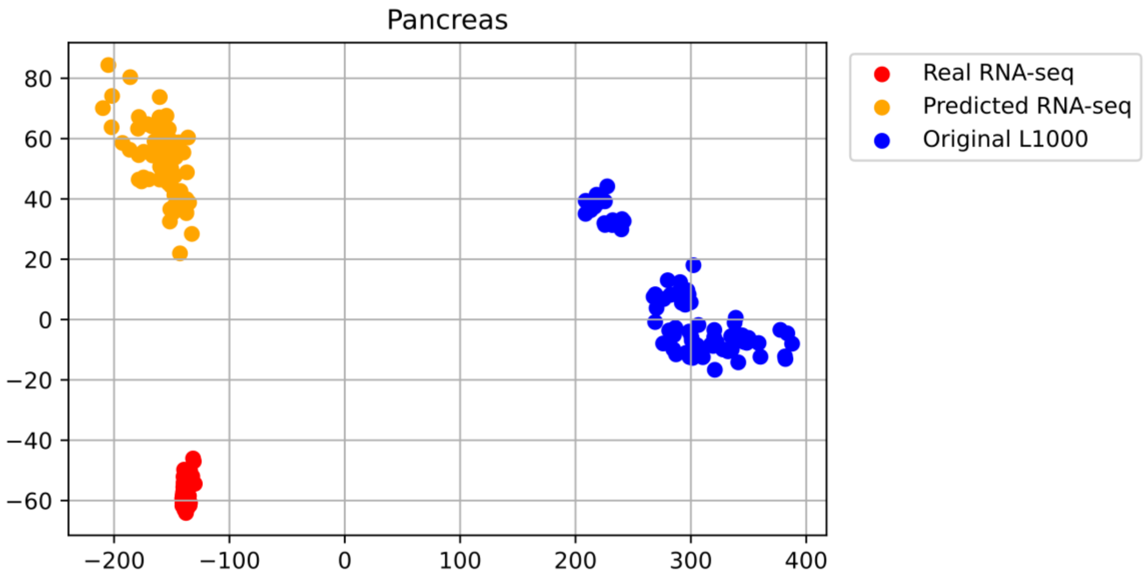
Pancreas
80
60
40
20
0
−20
−40
−60
−200
−100
0
100
200
300
400
Real RNA-seq
Predicted RNA-seq
Original L1000

Fig. S53

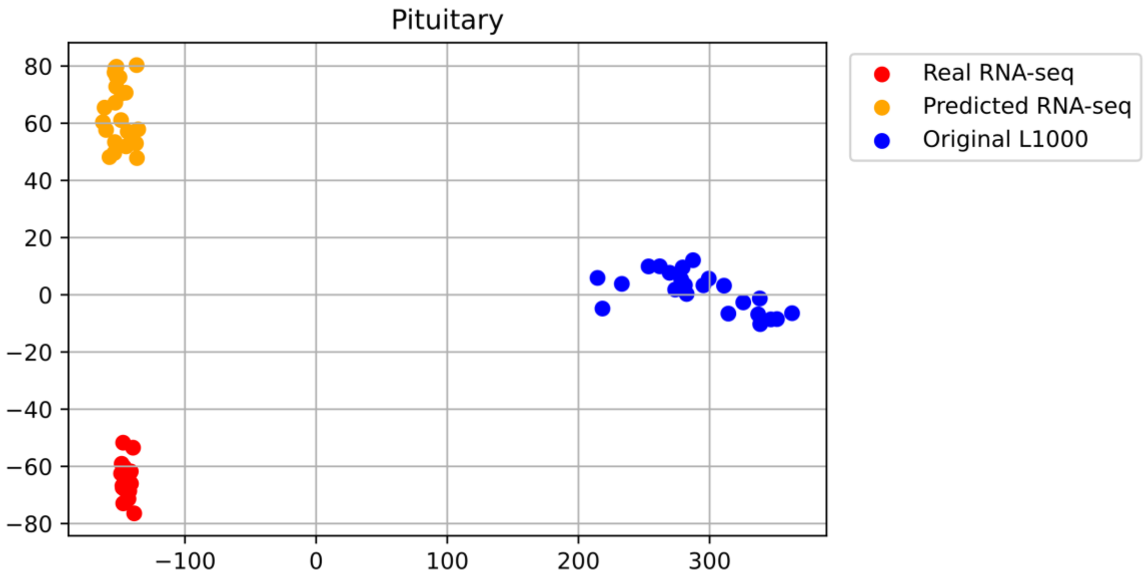
Pituitary
80
60
40
20
0
−20
−40
−60
−80
−100
0
100
200
300
Real RNA-seq
Predicted RNA-seq
Original L1000

Fig. S54

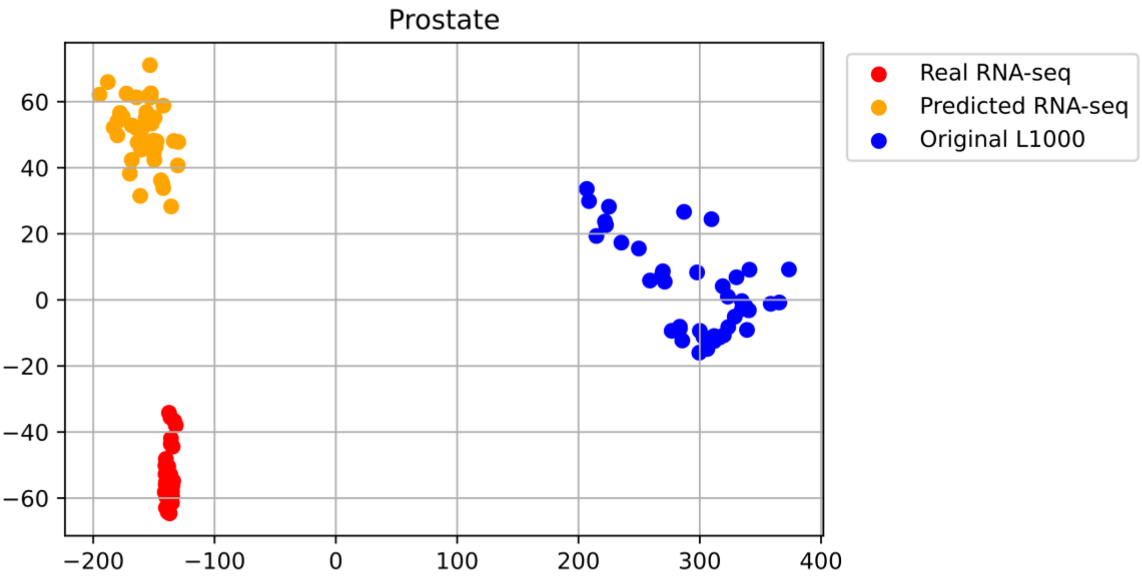
Prostate
60
40
20
0
−20
−40
−60
−200
−100
0
100
200
300
400
Real RNA-seq
Predicted RNA-seq
Original L1000

Fig. S55

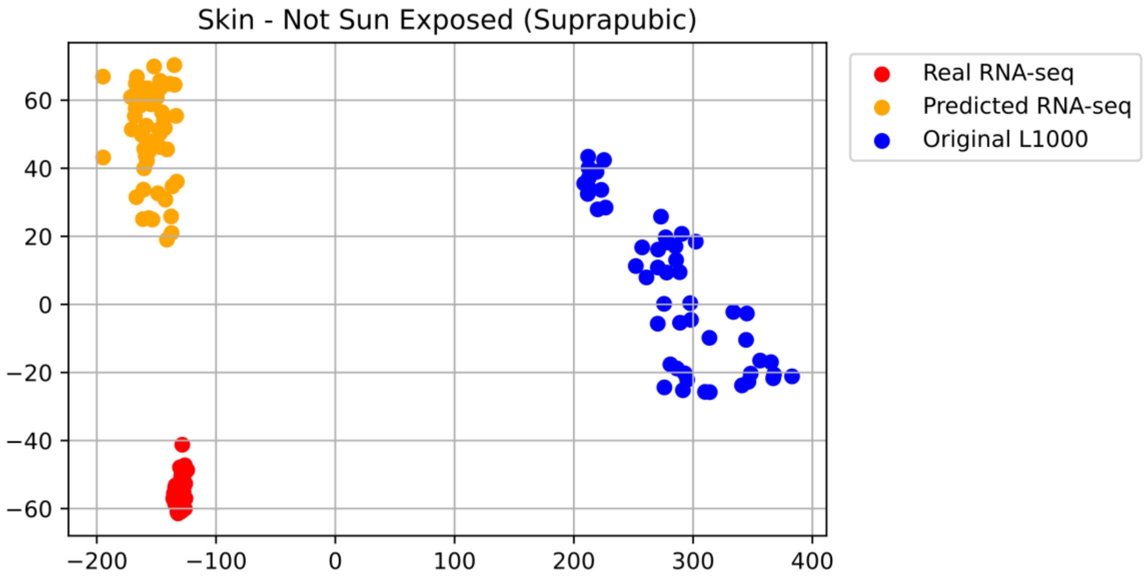
Skin - Not Sun Exposed (Suprapubic)
Real RNA-seq
Predicted RNA-seq
Original L1000
60
40
20
0
−20
−40
−60
−200
−100
0
100
200
300
400

Fig. S56

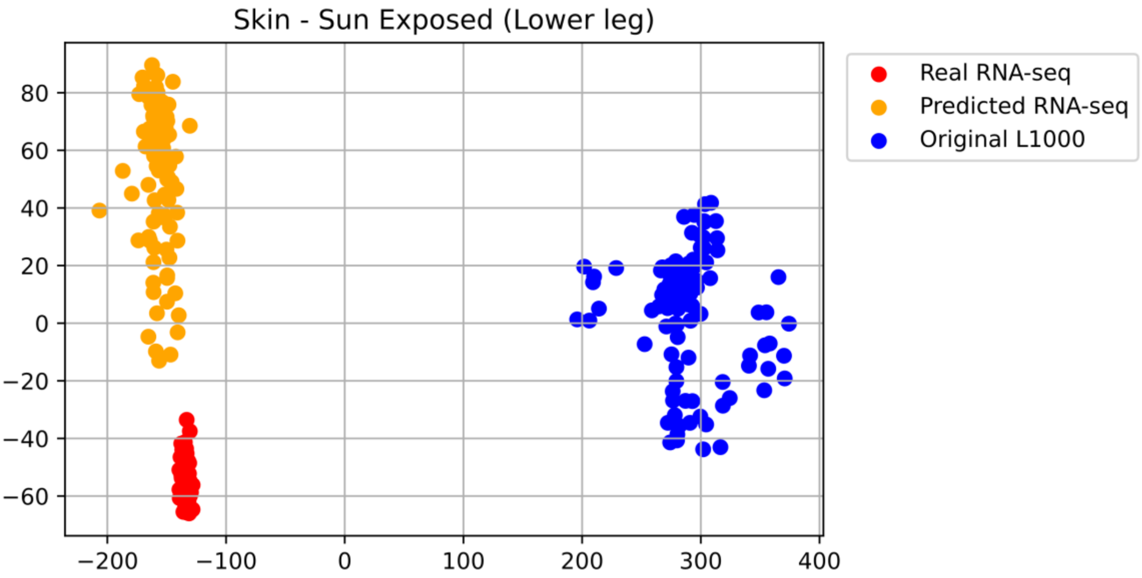
Skin - Sun Exposed (Lower leg)
80
60
40
20
0
−20
−40
−60
−200
−100
0
100
200
300
400
Real RNA-seq
Predicted RNA-seq
Original L1000

Fig. S57

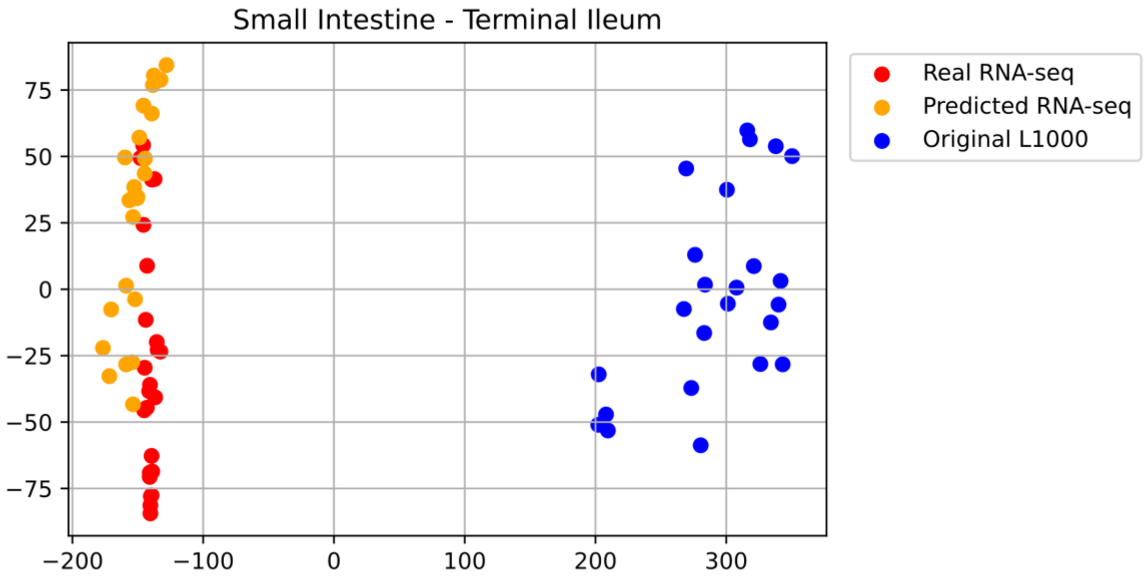
Small Intestine - Terminal Ileum
Real RNA-seq
Predicted RNA-seq
Original L1000
75
50
25
0
−25
−50
−75
−200
−100
0
100
200
300

Fig. S58

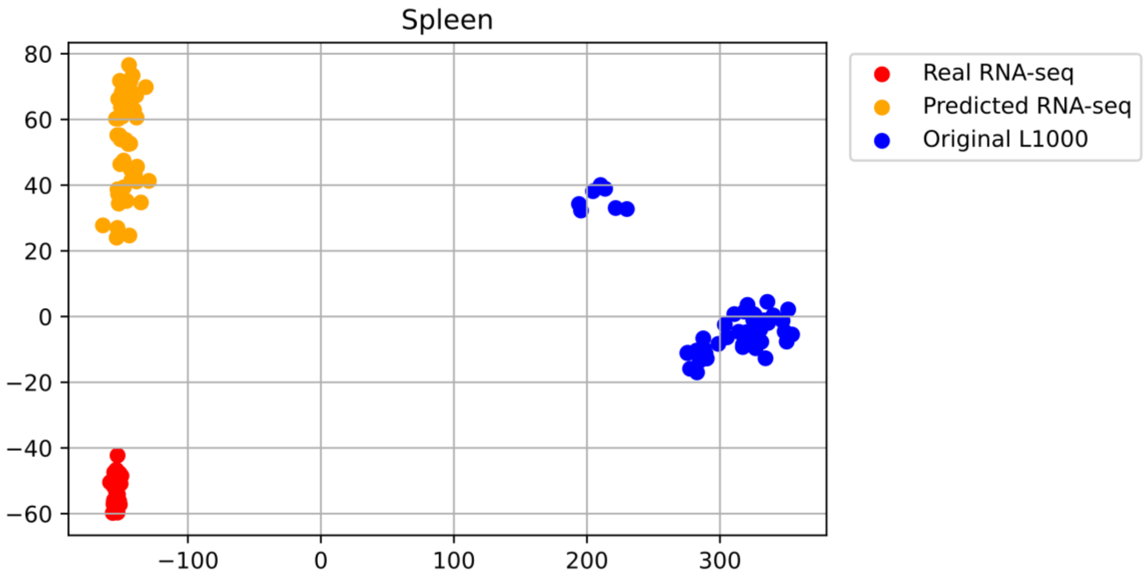
Spleen
80
60
40
20
0
−20
−40
−60
−100
0
100
200
300
Real RNA-seq
Predicted RNA-seq
Original L1000

Fig. S59

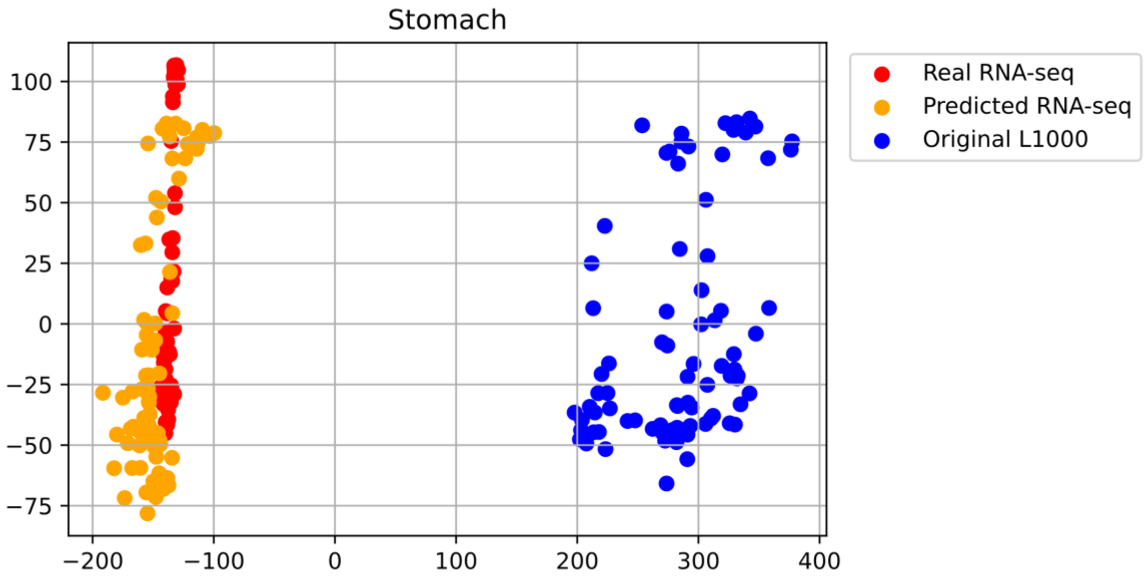
Stomach
Real RNA-seq
Predicted RNA-seq
Original L1000
100
75
50
25
0
−25
−50
−75
−200
−100
0
100
200
300
400

Fig. S60

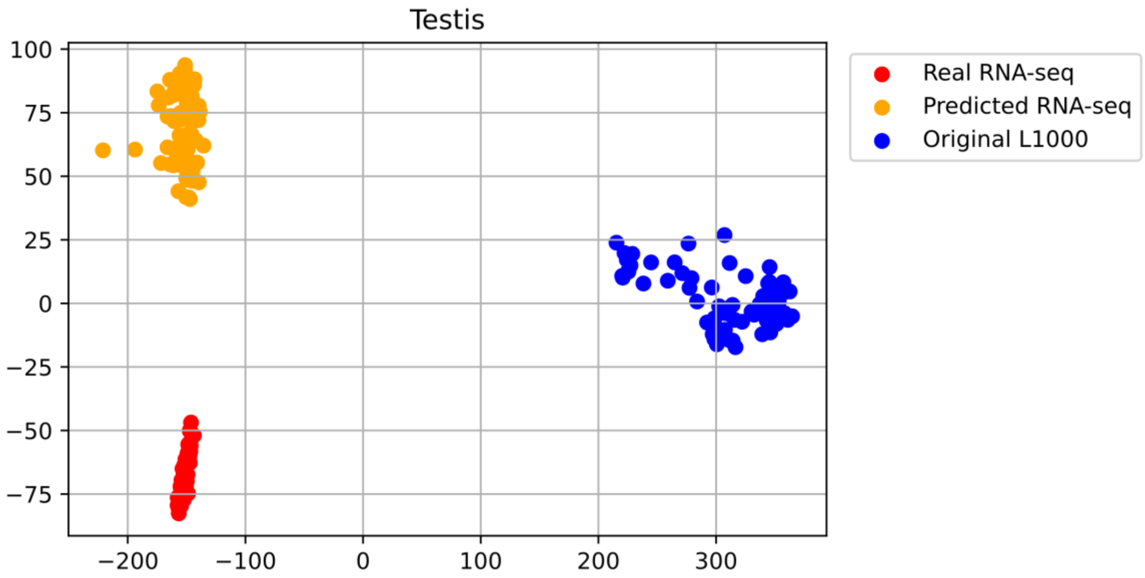
Testis
100
75
50
25
0
−25
−50
−75
−200
−100
0
100
200
300
Real RNA-seq
Predicted RNA-seq
Original L1000

Fig. S61

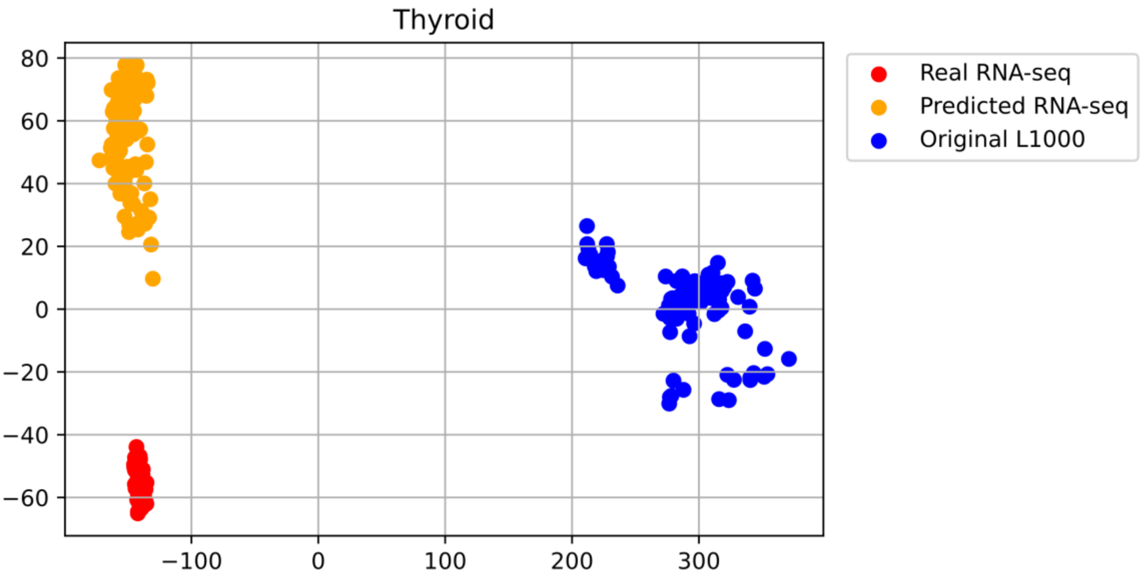
Thyroid
80
60
40
20
0
−20
−40
−60
−100
0
100
200
300
Real RNA-seq
Predicted RNA-seq
Original L1000

Fig. S62

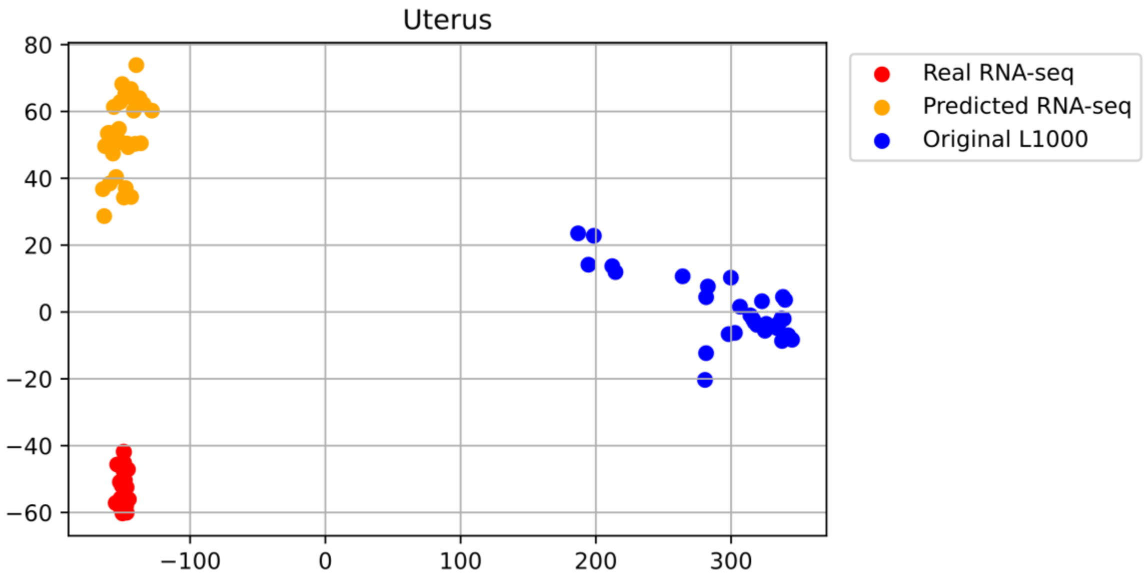
Uterus
80
60
40
20
0
−20
−40
−60
−100
0
100
200
300
Real RNA-seq
Predicted RNA-seq
Original L1000

Fig. S63

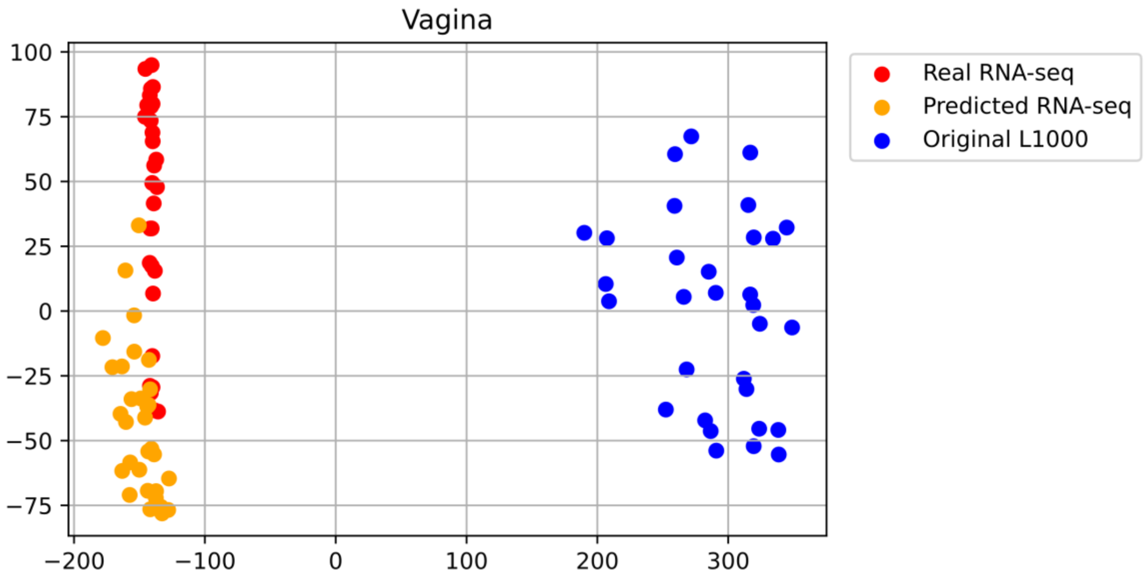
Vagina
100
75
50
25
0
−25
−50
−75
−200
−100
0
100
200
300
Real RNA-seq
Predicted RNA-seq
Original L1000

Fig. S64

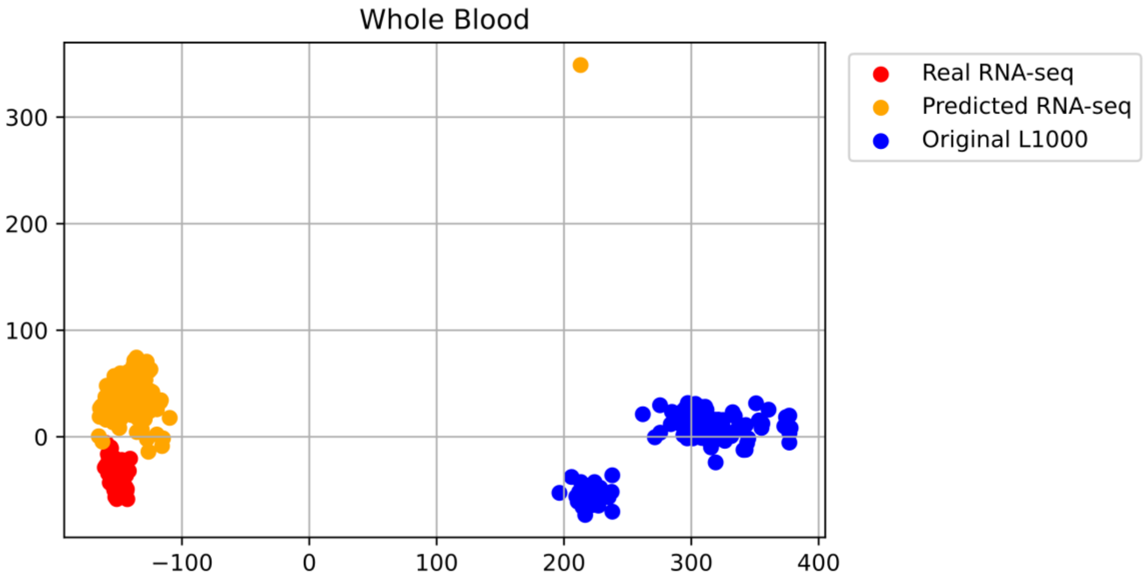
Whole Blood
300
200
100
0
−100
0
100
200
300
400
Real RNA-seq
Predicted RNA-seq
Original L1000

Fig. S65

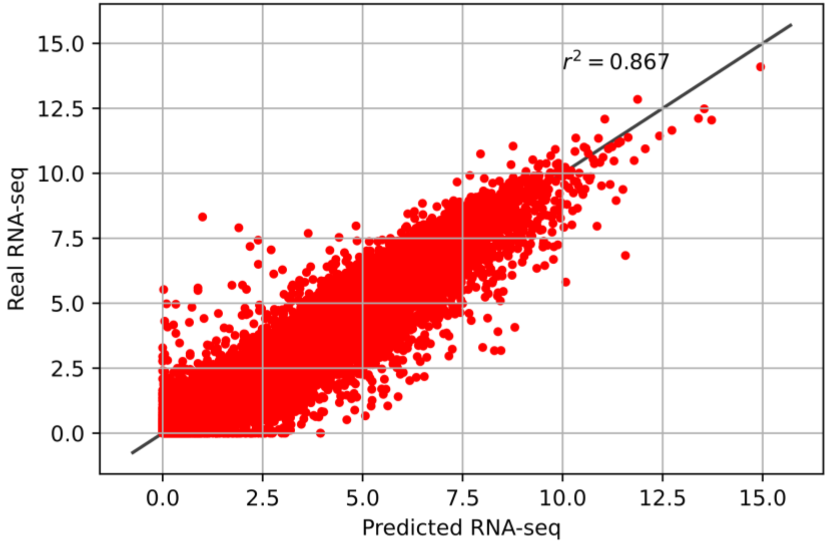

Scatter plot of Predicted RNA-seq expression values and Real RNA-seq expression values of a randomly selected sample with a high correlation

Fig. S66

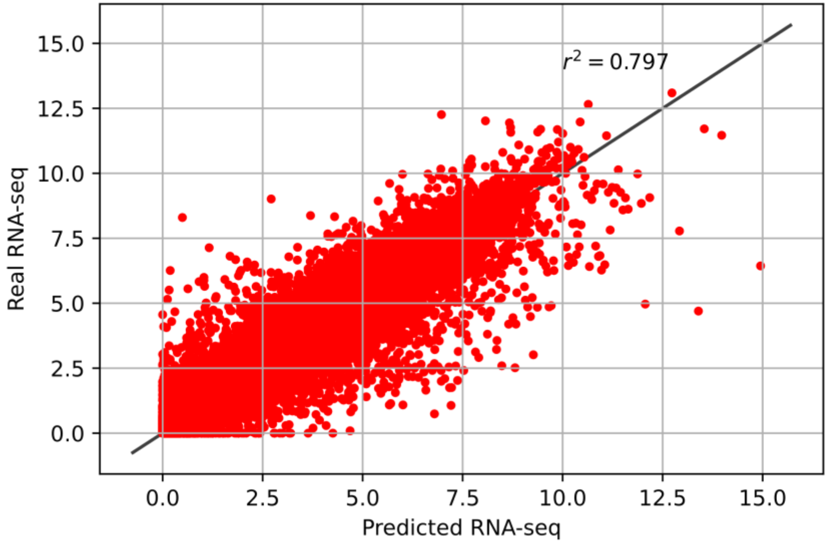

Scatter plot of Predicted RNA-seq expression values and Real RNA-seq expression values of a randomly selected sample with a medium correlation

Fig. S67

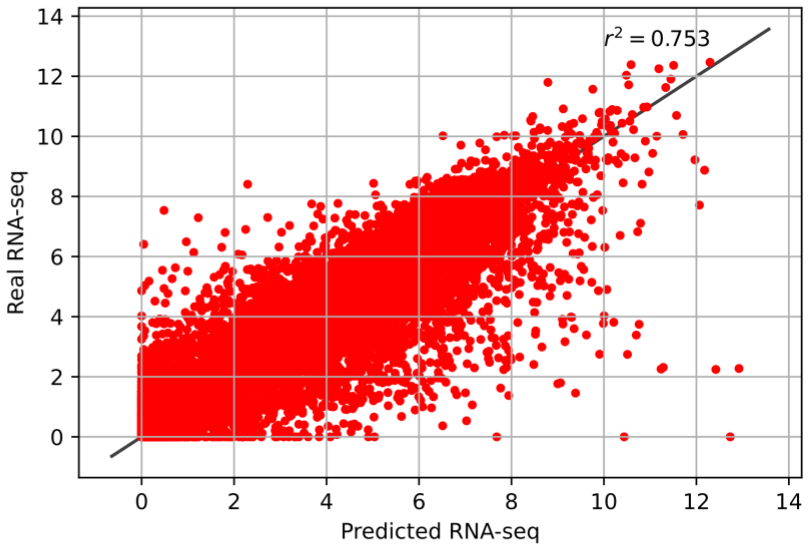

Scatter plot of Predicted RNA-seq expression values and Real RNA-seq expression values of a randomly selected sample with a low correlation

Fig. S68

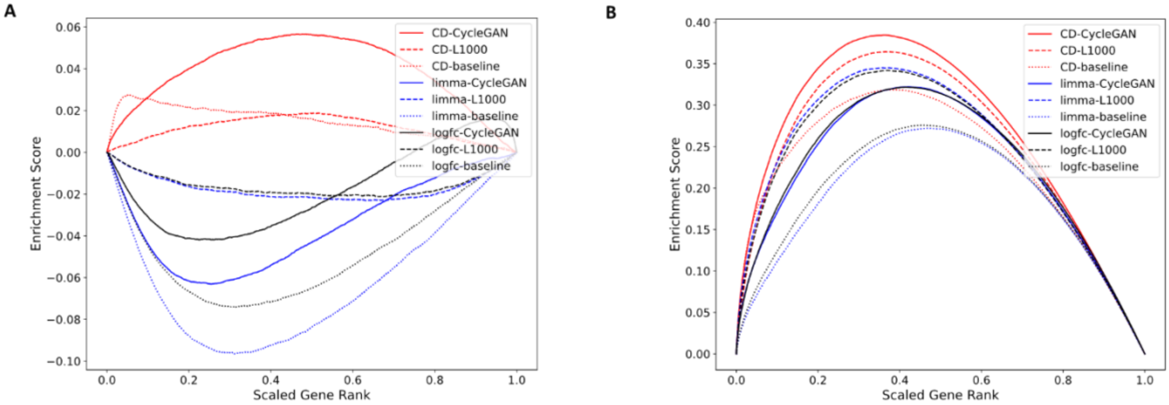

Benchmarking dexamethasone gene expression signatures from a real RNA-seq study (GSE94408)

Fig. S69

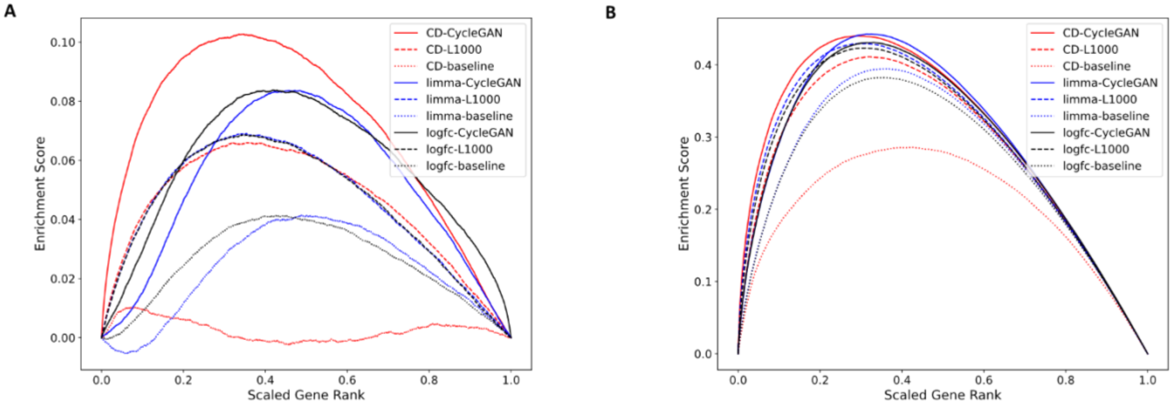

Benchmarking dexamethasone gene expression signatures from a real RNA-seq study (GSE193988; GFP overexpressed)

Fig. S70

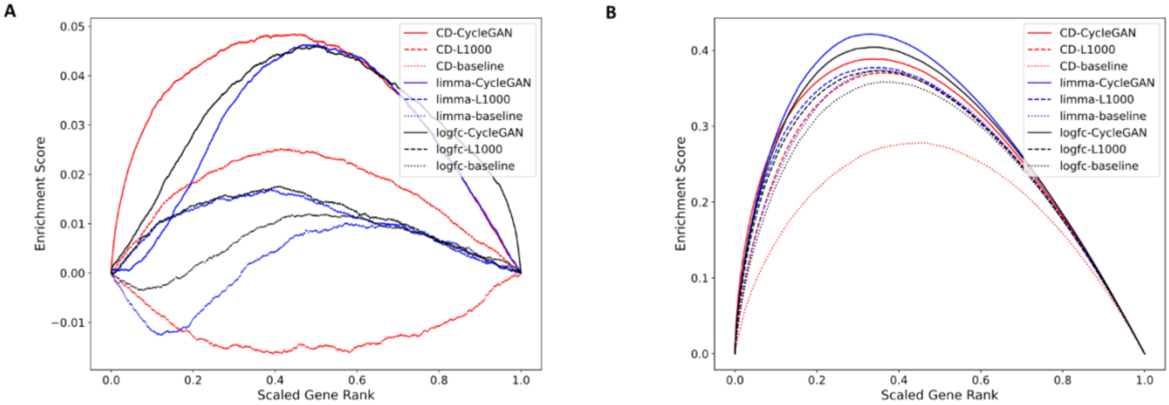

Benchmarking dexamethasone gene expression signatures from a real RNA-seq study (GSE193988; P300 overexpressed)

Fig. S71

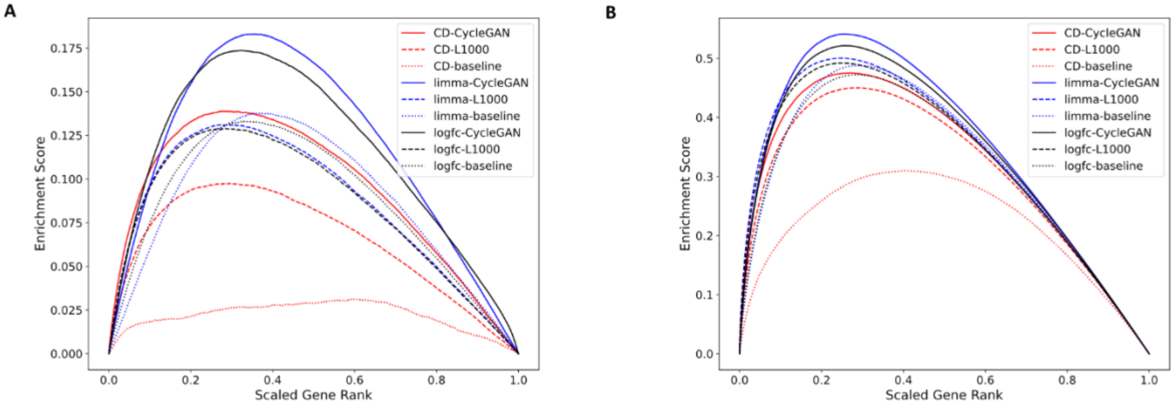

Benchmarking dexamethasone gene expression signatures from a real RNA-seq study (GSE186104)

Fig. S72
